# Hepatic factor MANF drives hepatocytes reprogramming by detaining cytosolic CK19 in intrahepatic cholangiocarcinoma

**DOI:** 10.1038/s41418-025-01460-4

**Published:** 2025-02-19

**Authors:** Qiong Mei, Yu Zhang, Hong Li, Wei Ma, Wenkai Huang, Zhengsheng Wu, Yongli Huang, Yanyan Liang, Chuansheng Wei, Jinfeng Wang, Yuefeng Ruan, Lin Yang, Yan Huang, Yujun Shen, Jun Liu, Lijie Feng, Yuxian Shen

**Affiliations:** 1 School of Basic Medical Sciences, Anhui Medical University, Hefei, Anhui China; 2 College & Hospital of Stomatology, Anhui Medical University, Hefei, Anhui China; 3 School of Pharmacy, Anhui Medical University, Hefei, Anhui China; 4 Department of General Surgery, The First Affiliated Hospital of Anhui Medical University, Hefei, Anhui China

**Keywords:** Oncogenes, Proteasome

## Abstract

Intrahepatic cholangiocarcinoma (ICC) is characterized by poor prognosis and limited treatment. Hepatocytes have been considered as one of the origins of ICC, however, the underlying mechanisms remain unclear. Here, we found mesencephalic astrocyte-derived neurotrophic factor (MANF), a hepatoprotective factor, was exceptionally upregulated in human ICC tissues and experimental mouse ICC models induced by sleeping beauty transposon (SBT) or thioacetamide (TAA) challenge. We identified MANF as a biomarker for distinguishing the primary liver cancer and verified the oncogenic role of MANF in ICC using cell lines overexpressing/knocked down MANF and mice specifically knocked in/out MANF in hepatocytes. Lineage tracing revealed that MANF promoted mature hepatocyte transformation into ICC cells. Mechanistically, MANF interacted with CK19 at Ser35 to suppress CK19 membrane recruitment. Cytosolic CK19 bound to AR domain of Notch2 intracellular domain (NICD2) to stabilize NICD2 protein level and trigger Notch signaling, which contributed to hepatocyte transformation to ICC cells. We uncover a novel profile of MANF and the original mechanism, which shed light on ICC diagnosis and intervention.

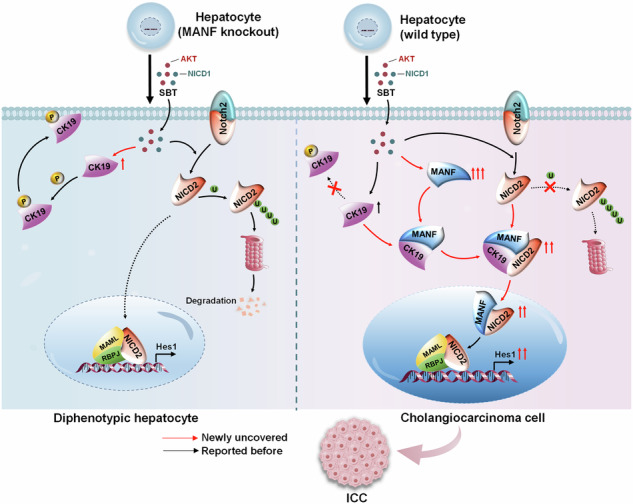

## Introduction

Cholangiocarcinoma (CCA) is an aggressive hepatic malignant biliary epithelial tumor, it occurs at all regions of the biliary tree and is classified into intrahepatic CCA (ICC), peri-hilar CCA (PCC), and distal CCA (DCC) [1]. ICC remains clinically challenge due to late-stage presentation and high post-surgery recurrence [2]. The existing single-cell RNA sequencing data showed that there are four subgroups in human ICC, including inflammatory subgroup (S1), the highest levels of proteins related to cancer-associated fibroblasts and extracellular matrix subgroup (S2), metabolic subgroup (S3), and maximum expression of adhesion and biliary-specific proteins subgroup (S4) [3]. ICC is a highly heterogeneous tumor, thus research into the molecular profiles and potential pathogenic factors of this tumor is urgently needed.

It was originally believed that ICC originates from the malignant transformation of biliary epithelial cells (BECs). Recent studies have described that ICC also originates from the transdifferentiation of hepatocytes and hepatic progenitor cells (HPCs) in response to liver injury [4]. Hepatocytes are plastic, and mature hepatocytes can generate bipotential HPCs in some microenvironmental conditions [5–7]. Numerous studies have uncovered that abnormal overexpression of Yap, PI3K/AKT, and Hedgehog signals can promote hepatocytes reprogramming to BECs and ICC cells [8–12]. Recent studies showed that Sox9^+^ biphenotypic hepatocytes originate from mature hepatocytes, and some of them are incorporated into ductular structures [9, 13]. Beyond that, TP53, Myc, KRAS, TGF-β, Wnt/β-catenin also regulate the reprogramming of hepatocyte in a certain tumor microenvironment [14–17]. Notch signaling was found to promote hepatocytes to convert into mature BECs or ICC cells that form functional bile ducts [18–21]. However, how are the mature hepatocytes transformed to BECs, HPCs, or ICC cells? The underlying mechanisms remain unclear.

Mesencephalic astrocyte-derived neurotrophic factor (MANF), an endoplasmic reticulum (ER) stress-inducible protein, was initially identified as a member of the new family of neurotrophic factors and plays a cytoprotective role in Parkinson’s disease, Alzheimer’s disease, cerebral ischemia, and diabetes [22–25]. Our previous studies showed that MANF protects ischemia- or drug-induced liver injury and inhibits peripheral inflammation by negatively regulating TLR4/NF-κB signal pathway [26–29]. It’s more interesting that we found MANF was downregulated in hepatocellular carcinoma (HCC) and inhibited HCC progression [30], while it was upregulated in ICC, another primary liver cancer. However, whether MANF plays a treacherously oncogenic role in ICC and how MANF behaves in mature hepatocytes transdifferentiation are not yet known.

To answer these questions, we observed the expressional profile of MANF in human and sleeping beauty transposon (SBT)- or thioacetamide (TAA)-induced mice ICC tissues. We also investigated the oncogenic role of MANF in ICC by using ICC cell lines, nude mice bearing tumor, hepatocyte-specific MANF knockin (KI)/knockout (KO) modeling mice, and administration of recombinant human MANF (rhMANF). Lineage tracing was employed to observe the transdifferentiation of mature hepatocytes. We found that MANF promotes the tumorigenesis and progression of ICC by driving the transformation of mature hepatocytes into ICC cells. Mechanistically, we identified a complex involving MANF, CK19, and Notch2. Specifically, MANF binds to CK19 at Ser35 to block CK19 membrane recruitment. The retention of CK19 in cytosol stabilizes Notch2 intracellular domain (NICD2) by binding to AR domain of NICD2 and consequently triggers its nuclear signaling.

## Results

### MANF is specifically upregulated in human ICC tissues and positively correlates with the clinicopathological features

**Fig. 1:**
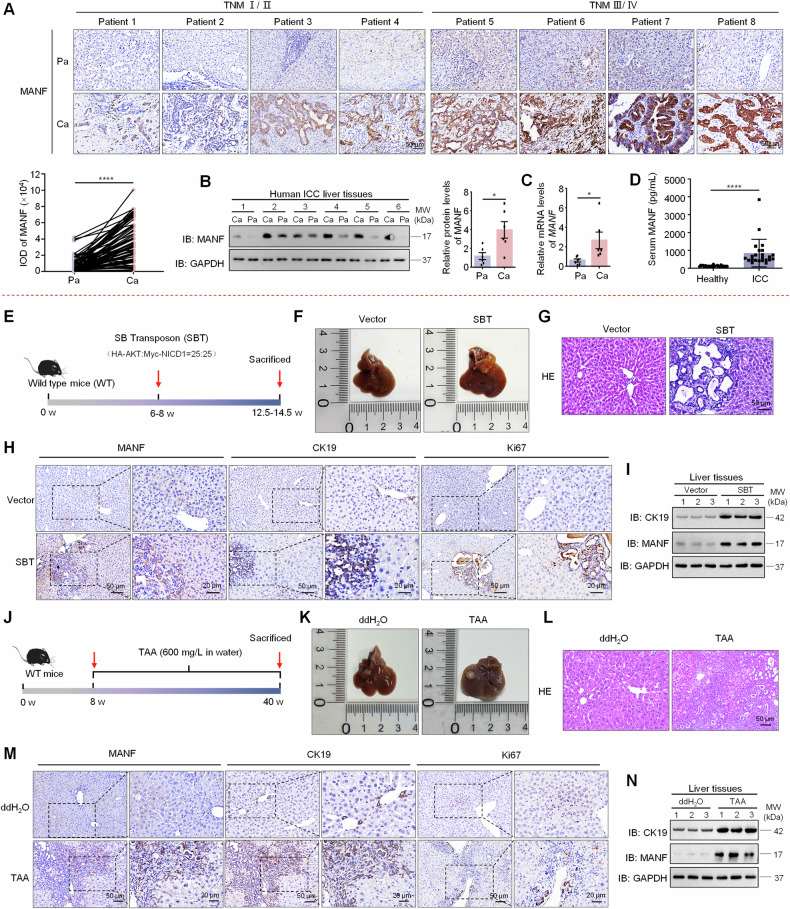
MANF is upregulated in ICC. **A** MANF expression in the liver tissues of ICC patients was detected by immunohistochemistry assay. *n* = 78, ****P < 0.0001. **B** MANF protein levels were detected in Ca and the Pa tissues by western blot assay. *n* = 6, *P < 0.05. **C** The mRNA levels of MANF in the liver tissues of ICC patients were detected by qPCR assay. *n* = 6, *P < 0.05. **D** MANF level in the serum of ICC patients (*n* = 23) and healthy controls (*n* = 25) was detected by ELISA assay. ****P < 0.0001. **E** Scheme for SBT-induced ICC. **F** Gross morphology of mice livers. **G** HE staining of mice liver tissues. The levels of MANF, CK19, and Ki67 in SBT-induced mice ICC were detected by immunohistochemistry staining (**H**), and western blot assays (**I**). **J** Scheme for TAA-induced mice ICC. **K** Gross morphology of mice livers. **L** HE staining of mice liver tissues. The expressions of MANF, CK19, and Ki67 in TAA-induced mice ICC were detected by immunohistochemistry staining (**M**), and western blot assays (**N**).

To initially investigate the role of MANF in ICC, we collected paraffin sections from 78 patients and frozen samples from 6 patients with simple ICC, four samples with both ICC and HCC, and serum from 23 ICC patients and 25 healthy individuals. As shown in Fig. 1A–D, MANF had a high expression in cancer tissues (Ca) compared with adjacent para-cancer tissues (Pa) in either protein, mRNA, or the levels in serum. In four samples mixed with HCC and ICC identified by HE staining and immunohistochemistry with the antibodies of HNF4α and CK19, we observed that MANF was also highly expressed in ICC, while very low level in HCC under the same view field and in the same slide (Supplementary Fig. 1A). RNA sequencing data from GSE179443 database also showed that MANF was highly expressed in patient ICC other than HCC tissues (Supplementary Fig. 1B, C). Meanwhile, correlation analysis between MANF level and clinical variables in ICC showed that high expression of MANF was associated with larger tumor diameter (*P* = 0.026), TNM stage (*P* = 0.000), and distant metastasis (*P* = 0.017) (Supplementary Table 1). Similar results were observed in the TCGA, GSE107943, and GSE241923 datasets (Supplementary Fig. 2A, B, G, H). We further analyzed the correlation between MANF level and survival probability of ICC patients. High level of MANF predicted poor prognosis was identified by Kaplan–Meier curves and receiver operating characteristic curves (ROC) (Supplementary Fig. 2C–F). Notably, MANF expression in undifferentiated ICC (ICC-UDC) was significantly higher than that in differentiated ICC (ICC-DC) (GSE221589) (Supplementary Fig. 2I–K). Moreover, we found that there was no significant change in MANF expression in extrahepatic cholangiocarcinoma (Supplementary Fig. 1D). These clinical characteristics suggest that MANF is a predictive biomarker for ICC diagnosis.

### MANF is upregulated in mouse ICC tissues in experimental models

To further observe the profile of MANF in ICC tissues, we utilized the SBT system consisting of HA-AKT, Myc-NICD1, and transferase to construct a mouse ICC model (Fig. 1E). We monitored the dynamic changes in body weight and found that the mice lost weight at a later stage after SBT injection (Supplementary Fig. 3A), and the liver-body weight ratio was increased compared with vector controls (Supplementary Fig. 3B). As expected, ICC mice exhibited elevated serum alanine transaminase (ALT), aspartate aminotransferase (AST), total bilirubin (TBIL), and direct bilirubin (DBIL) levels compared with the controls (Supplementary Fig. 3C). Meanwhile, increased liver size, more tumor number and area, and HE staining showed obvious cystic adenoid change in ICC tissues (Fig. 1F, G and Supplementary Fig. 3D, E). Furthermore, SBT system-derived ICC was identified by the expressions of HA, Myc, CK19 (a biomarker of BECs and ICC), and Ki67 (a biomarker for proliferation) (Fig. 1H and Supplementary Fig. 3F, H). Notably, MANF expression was significantly higher in Ca than Pa tissues after SBT induction (Supplementary Fig. 3G). The mRNA and protein levels of MANF were significantly increased in SBT-induced ICC tissues compared with the vector controls (Fig. 1H, I and Supplementary Fig. 3H–J).

We next used the TAA-induced ICC model to verify MANF expression in mouse ICC (Fig. 1J). We observed a decrease in body weight (Supplementary Fig. 3K), an increase in liver-body weight ratio (Supplementary Fig. 3L), elevated serum levels of ALT, AST, TBIL, and DBIL (Supplementary Fig. 3M), increased tumor nodules and numbers (Fig. 1K and Supplementary Fig. 3N), as well as significant ductular reaction and larger tumor area (Fig. 1L and Supplementary Fig. 3O) in TAA-treated mice, confirming successful induction of the ICC model. MANF expression was also significantly higher in Ca than Pa tissues in TAA-induced mice ICC (Supplementary Fig. 3P). Additionally, the levels of MANF, CK19, and Ki67 in TAA-treated mice were increased (Fig. 1M, N and Supplementary Fig. 3Q–S), which is similar to that in SBT-induced mice ICC model.

### MANF promotes the malignant biological behaviors of ICC cell lines in vitro and in vivo

**Fig. 2:**
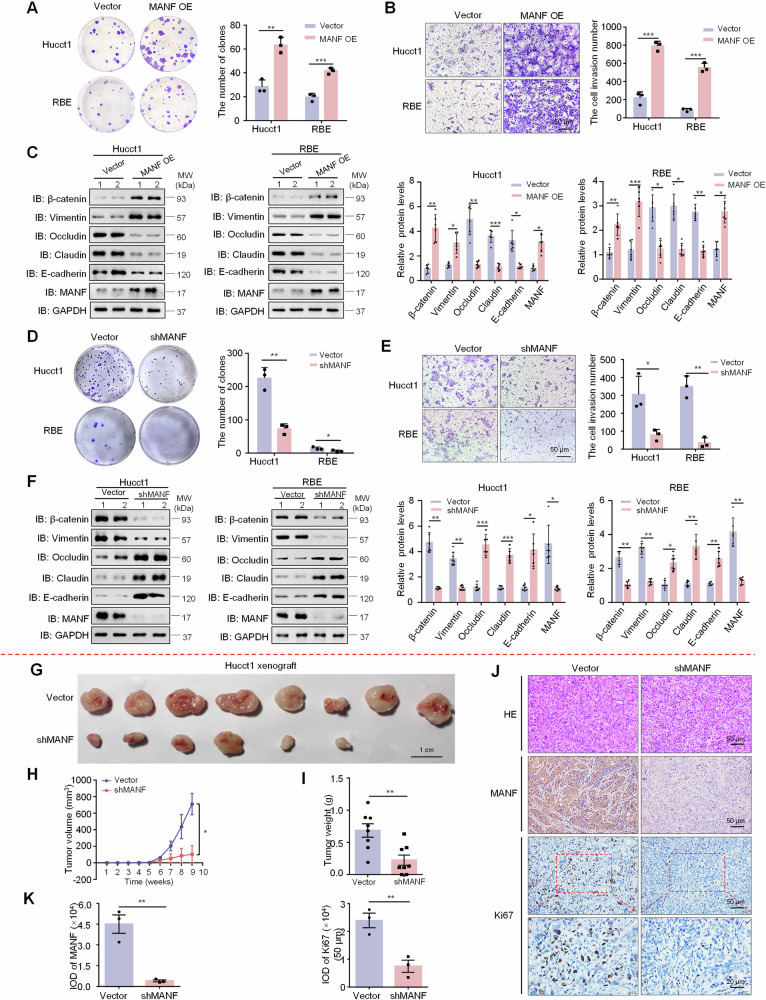
MANF promotes the malignant biological behavior of ICC cells in vitro and in vivo. The proliferation and invasion of ICC cells overexpressing MANF were examined by colony formation (**A**) and transwell assays (**B**), respectively. *n* = 3, **P < 0.01, ***P < 0.001. **C** Effects of MANF overexpression on EMT of ICC cells. The protein levels of MANF, E-cadherin, Claudin, Occludin, Vimentin, and β-catenin were detected by western blot assay. *n* = 6, *P < 0.05, **P < 0.01, ***P < 0.001. The proliferation and invasion of ICC cells with MANF knockdown were examined by colony formation (**D**) and transwell assays (**E**), respectively. *n* = 3, *P < 0.05, **P < 0.01. **F** Effects of MANF knockdown on EMT of ICC cells. The protein levels of MANF, E-cadherin, Claudin, Occludin, Vimentin, and β-catenin were detected by western blot assay. *n* = 6, *P < 0.05, **P < 0.01, ***P < 0.001. MANF knockdown reduces tumor size (**G**), volume (**H**), and weight (**I**) in subcutaneous xenograft mice. *n* = 8, *P < 0.05, **P < 0.01. **J**, **K** The levels of MANF and Ki67 were detected by immunohistochemistry assay. *n* = 3, **P < 0.01.

The above data suggest an oncogenic role of MANF in ICC. To test this, we firstly detected MANF level in Hucct1, HCCC9810, and RBE cell lines. MANF expression in Hucct1 cells was highest, followed by RBE cells (Supplementary Fig. 4A). Next, we prepared lentiviral transduction particles containing MANF DNA (MANF OE) and short hairpin RNA (shMANF) to be stably transfected into ICC cell lines, respectively (Supplementary Fig. 4B–E). In the colony formation and transwell experiments, MANF OE cells had stronger proliferation and invasion than the vector controls (Fig. 2A, B), while shMANF showed the opposite effects (Fig. 2D, E). Meanwhile, MANF OE increased the protein levels of mesenchymal markers β-catenin and Vimentin, but decreased the protein levels of epithelial markers Occludin, Claudin, and E-cadherin (Fig. 2C), which was opposite to the effects caused by shMANF (Fig. 2F).

Next, we subcutaneously injected Hucct1 cells stably knocked down MANF into nude mice. We found the mean size, volume, and weight of tumors were reduced after MANF knockdown than vector controls (Fig. 2G–I). And MANF knockdown decreased the levels of MANF and Ki67 (Fig. 2J, K). The above results indicate that high level MANF promotes the malignant biological behaviors of ICC cells in vitro and in vivo.

### Hepatocyte-specific MANF knockin accelerates SBT-induced ICC

**Fig. 3:**
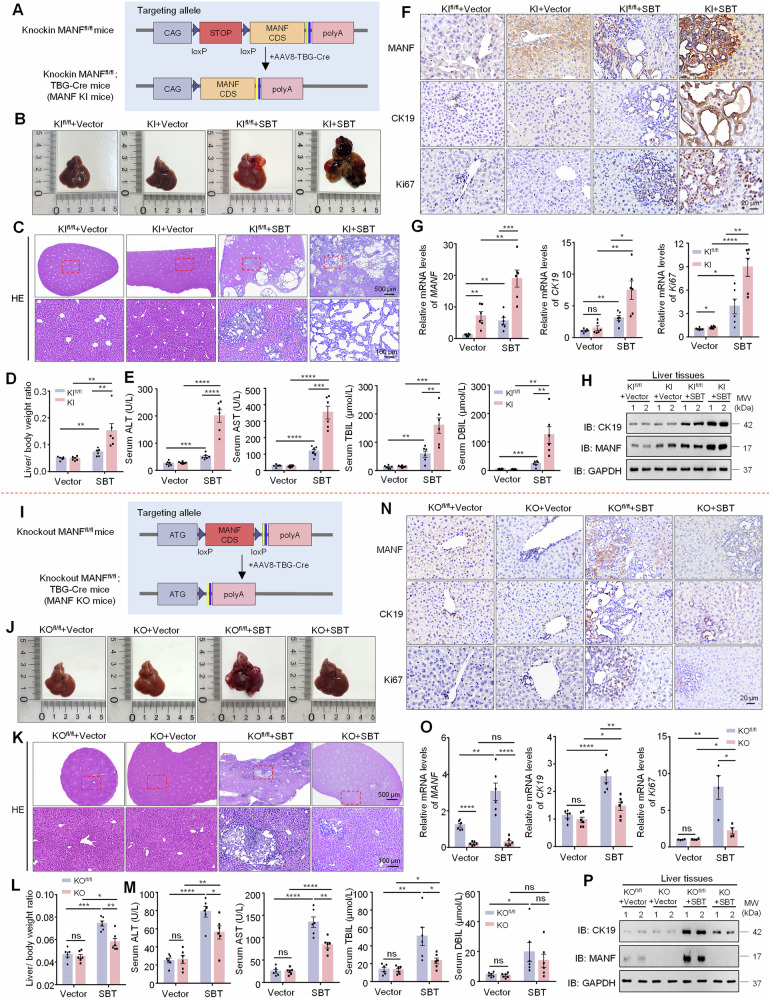
Effects of hepatocyte-specific MANF knockin/knockout on SBT-induced mice ICC. **A** Construction strategy of mature hepatocyte-specific MANF knockin in mice. **B** Gross morphology of mice livers. **C** HE staining of mice liver tissues. **D** Liver-body weight ratio. *n* = 6, **P < 0.01. **E** Serum ALT, AST, TBIL, and DBIL levels. *n* = 6, **P < 0.01, ***P < 0.001, ****P < 0.0001. The levels of MANF, CK19, and Ki67 in MANF knockin mice treated with SBT were detected by immunohistochemistry staining (**F**), qPCR (**G**, *n* = 6, *P < 0.05, **P < 0.01, ***P < 0.001, ****P < 0.0001), and western blot assays (**H**). **I** Construction strategy of mature hepatocyte-specific MANF knockout in mice. **J** Gross morphology of mice livers. **K** HE staining of mice liver tissues. **L** Liver-body weight ratio. *n* = 6, *P < 0.05, **P < 0.01, ***P < 0.001. **M** Serum ALT, AST, TBIL, and DBIL levels. *n* = 6, *P < 0.05, **P < 0.01, ****P < 0.0001. The levels of MANF, CK19, and Ki67 in MANF knockout mice treated with SBT were detected by immunohistochemistry staining (**N**), qPCR (**O**, *n* = 4–6, *P < 0.05, **P < 0.01, ****P < 0.0001), and western blot assays (**P**).

To confirm the effect of MANF on ICC, we constructed mature hepatocyte- specific MANF knockin (KI) mice via CRISPER-Cas9 technology under the control of AAV8-TBG-Cre (Fig. 3A and Supplementary Fig. 5A, C). The MANF KI mice were injected with SBT to induce ICC model. We found the liver size, tumor number, tumor area, and liver-body weight ratio in MANF KI mice were significantly increased, compared with MANF KI^fl/fl^ mice after SBT treatment (Fig. 3B–D, Supplementary Fig. 6A, B). In addition, serum levels of ALT, AST, TBIL, and DBIL also significantly ascended in MANF KI mice than KI^fl/fl^ mice after SBT treatment (Fig. 3E). Moreover, the mRNA and protein levels of MANF, CK19, and Ki67 were elevated in MANF KI mice than KI^fl/fl^ mice after SBT induction (Fig. 3F–H and Supplementary Fig. 6C–E). These data indicate that hepatocyte-specific MANF KI promotes the tumorigenesis and development of ICC in mice.

### Hepatocyte-specific MANF knockout attenuates SBT-induced ICC

The mature hepatocyte-specific MANF KO mice were also made in the same way as MANF KI mice (Fig. 3I and Supplementary Fig. 5B, C). MANF KO mice exhibited smaller liver size, less tumor number and area (Fig. 3J, K and Supplementary Fig. 6F, G), lower liver-body weight ratio (Fig. 3L), and poorer serum levels of ALT, AST, TBIL, and DBIL (Fig. 3M) than KO^fl/fl^ mice under SBT induction, which was opposite to MANF KI mice. In correspondence with tumor growth, the mRNA and protein levels of MANF, CK19, and Ki67 were reduced in MANF KO mice, compared with KO^fl/fl^ mice under SBT induction (Fig. 3N–P, and Supplementary Fig. 6H–J). These results indicate that mature hepatocyte-specific MANF KO inhibits SBT-induced mice ICC.

### Hepatic MANF knockout decelerates TAA-induced ICC and rhMANF supplement rescues this change

**Fig. 4:**
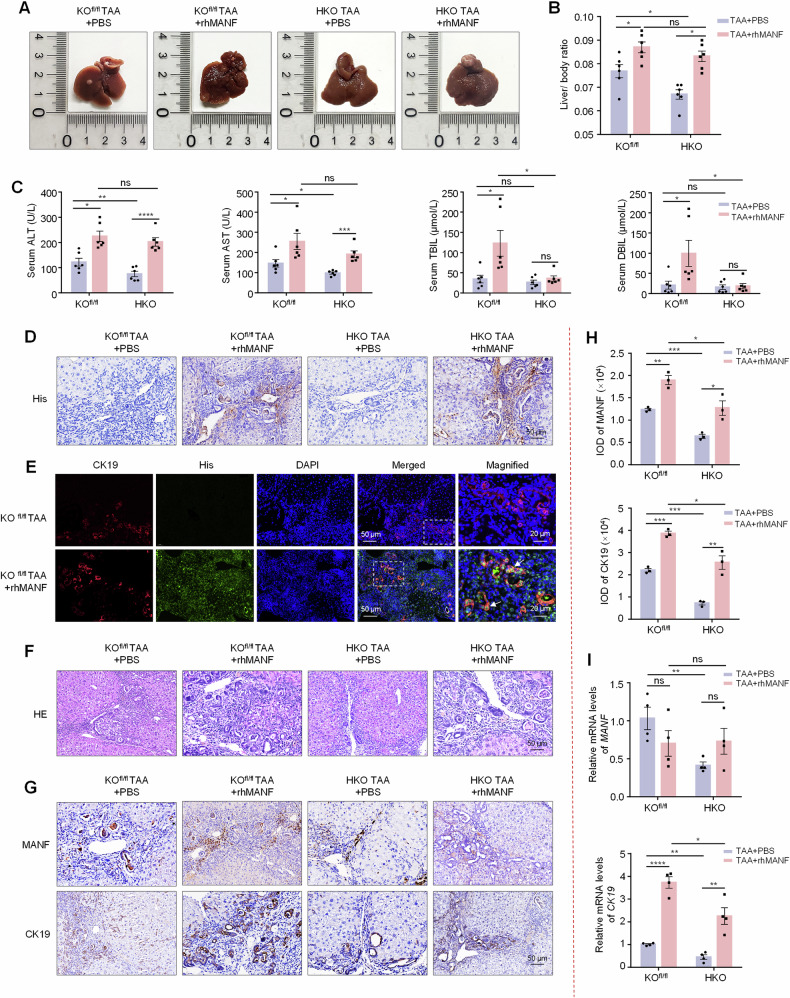
Effects of hepatic MANF knockout and rhMANF on TAA-induced mice ICC. **A** Gross morphology of mice livers. **B** Liver-body weight ratio. *n* = 6, *P < 0.05. **C** Serum ALT, AST, TBIL, and DBIL levels. *n* = 6, *P < 0.05, **P < 0.01, ***P < 0.001, ****P < 0.0001. **D** The efficacy of MANF-His protein entering liver tissues. **E** Co-localization of His (green) and CK19 (red) detected by immunofluorescence. The nuclei were stained with DAPI (blue). **F** HE staining of mice liver tissues. **G**, **H** Immunohistochemistry was used to detect the levels of MANF and CK19 in TAA-induced ICC mice, with or without rhMANF injection. *n* = 3, *P < 0.05, **P < 0.01, ***P < 0.001. **I** The mRNA levels of MANF, CK19, and Ki67 were detected by qPCR assay. *n* = 4, *P < 0.05, **P < 0.01, ****P < 0.0001.

Because of the diversity of ICC molecular phenotypes, we further observed the role of MANF in TAA-induced ICC. Considering the short effect of AAVs and the long-term induction of TAA for ICC, we constructed hepatic MANF KO (HKO) mice by using Alb-Cre tool mice (Supplementary Fig. 6K–N). Meanwhile, the rhMANF was administrated to HKO mice to investigate the rescue of MANF after MANF deficiency. Recombinant MANF was injected by tail vein twice a week for 28 weeks at a dose of 2 mg/kg. The distribution of rhMANF in liver tissue was detected by immunohistochemistry and immunofluorescence with anti-His antibody (Fig. 4D, E). We found the tumor size and number, liver-body weight ratio, serum ALT and AST levels, and tumor area were significantly reduced in HKO mice compared with KO^fl/fl^ mice under TAA induction, which was inhibited by rhMANF (Fig. 4A–C, F and Supplementary Fig. 6O, P). We also found that rhMANF treatment increased the liver-body weight ratio, tumor number and area, and serum ALT and AST levels, compared with PBS controls in KO^fl/fl^ mice treated with TAA (Fig. 4A–C, F and Supplementary Fig. 6O, P). The levels of mRNA and proteins of CK19 were increased after rhMANF treatment (Fig. 4G–I). These data further demonstrate that hepatic MANF KO restrains ICC, and rhMANF supplementation promotes ICC and cancels the effect of MANF KO on ICC.

### Specific lineage tracing reveals hepatocytes reprogramming in ICC

**Fig. 5:**
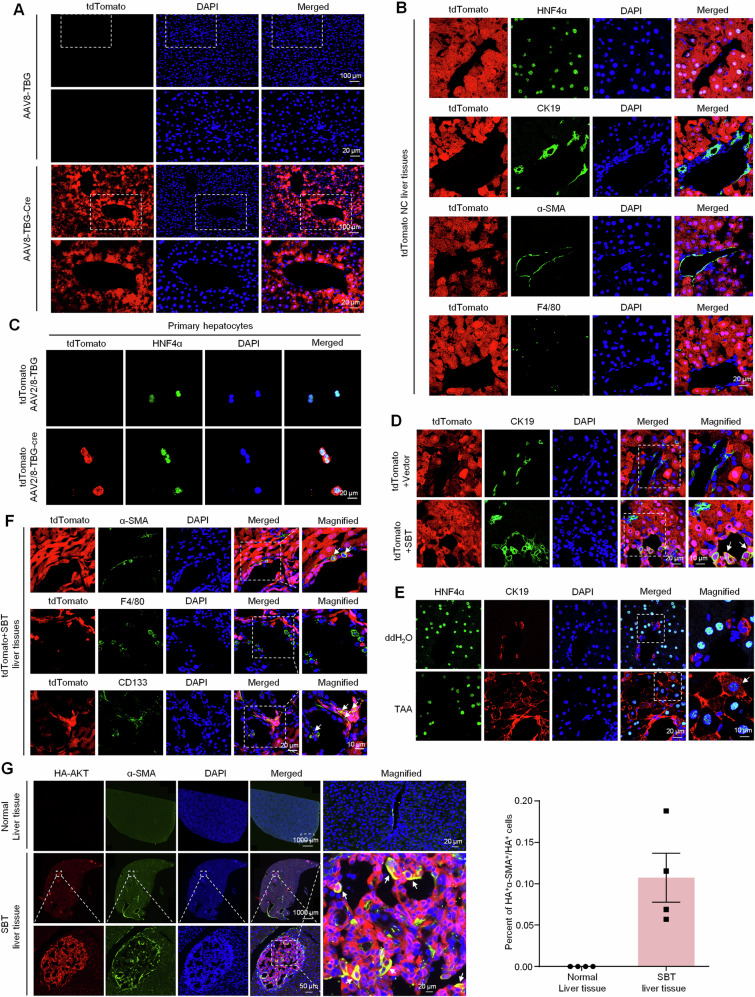
Specific lineage tracing reveals hepatocyte reprogramming in mice ICC. **A**, **B** Identification of the specificity of tdTomato (red) in mature hepatocytes after AAV8-TBG-Cre injection. Hepatocytes were labeled with anti-HNF4α antibody (green). The nonparenchymal cells were labeled with anti-CK19 (green, BECs marker), α-SMA (green, hepatic stellate cells marker), and F4/80 (green, macrophages marker) antibodies, respectively. **C** Identification of high specificity of AAV8-TBG-Cre for HNF4α^+^ hepatocytes. **D** Identification of double-positive hepatocytes in the liver tissues of SBT-induced ICC mice with tdTomato (red) and anti-CK19 (green) antibody. **E** Identification of HNF4α^+^CK19^+^ cells in TAA-induced ICC mice with anti-HNF4α (green) and anti-CK19 (red) antibodies, respectively. **F** Multiple lineages reprogramming of hepatocytes in SBT-induced mice ICC double labeled by tdTomato and anti-α-SMA (green), anti-F4/80 (green), and anti-CD133 (green), respectively. **G** The proportion of reprogramming from hepatocytes to myofibroblasts in mice ICC induced by SBT was calculated by anti-HA (red) and anti-α-SMA (green). The nuclei were stained with DAPI (blue).

Above results have confirmed the contributory effect of MANF on ICC, but the underlying mechanism remains unclear. Given that hepatocytes are the major sources of ICC and MANF is mainly expressed in hepatocytes [29], the genealogical tracing in mature hepatocytes was achieved by using tdTomato fluorescent reporter mice injected with AAV8-TBG-Cre. The tdTomato tracing hepatocytes were identified in Fig. 5A–C. We validated the specific expression of tdTomato in HNF4α-labeled hepatocytes, but not in CK19-labeled BECs, α-SMA-labeled myofibroblasts, and F4/80-labeled macrophages in liver tissues (Fig. 5B).

Then, the tdTomato mice were used to prepare SBT-induced ICC. The colocalization of tdTomato and CK19 was detectable in SBT-induced ICC mice, but not the vector controls (Fig. 5D), suggesting that the hepatocytes were reprogrammed into ICC cells after SBT challenge. The HNF4α^+^CK19^+^ cells were also found in TAA-induced ICC (Fig. 5E). We also found that mature hepatocytes were transformed into CD133^+^ stem cells (cancer stem cells) and α-SMA^+^ myofibroblasts, but not F4/80^+^ macrophages (Fig. 5F). Further study showed 11% HA^+^ hepatocytes were transformed into α-SMA^+^ myofibroblasts after SBT challenge (Fig. 5G).

### MANF promotes the transformation of hepatocytes into ICC cells in vivo and in vitro

**Fig. 6:**
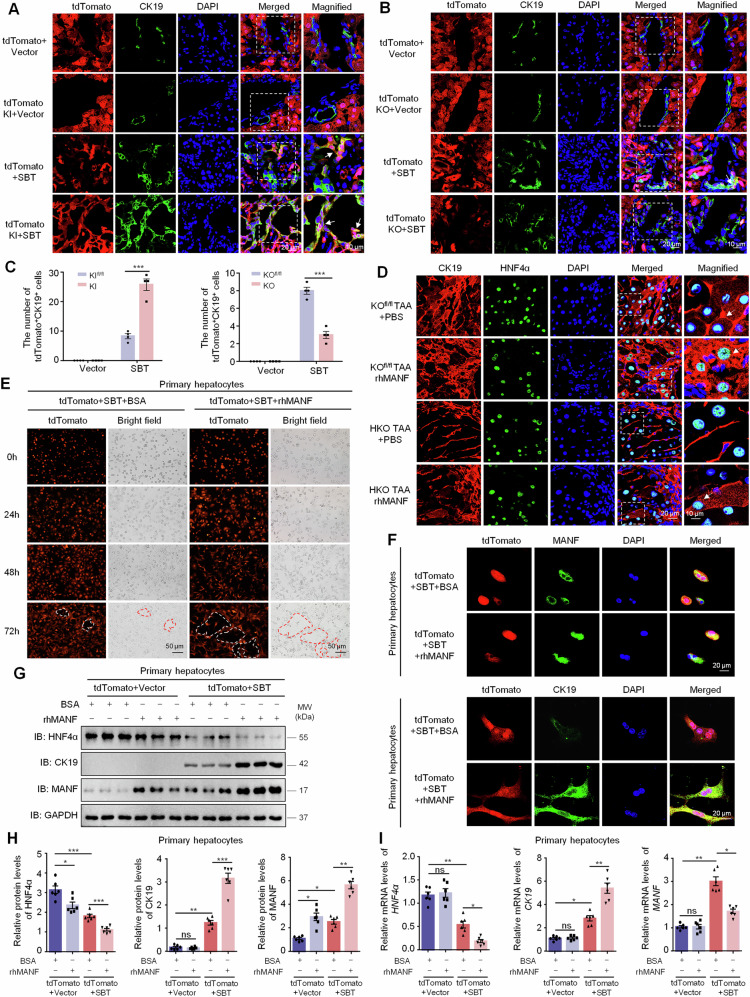
MANF promotes the transformation of hepatocytes into ICC cells in vivo and in vitro. Effects of MANF knockin (**A**, **C**) and knockout (**B**, **C**) on hepatocytes transformation in the liver tissues of SBT-induced ICC mice. The hepatocytes were double labeled by tdTomato (red) and anti-CK19 (green) antibody. *n* = 4, ***P < 0.001. **D** Effects of MANF knockout and rhMANF on hepatocytes transformation in the liver tissues of TAA-induced ICC mice. The hepatocytes were co-labeled with anti-HNF4α (green) and anti-CK19 (red) antibodies. **E** Effects of rhMANF on the hepatocytes morphology of primary hepatocytes isolated from tdTomato (red) mice challenged with SBT for 2 weeks. The cells were treated with rhMANF (80 ng/mL) for 24, 48, and 72 h, respectively. BSA (80 ng/mL) was used as the controls. **F** The promotive effect of rhMANF on the levels of cytosolic CK19 in primary hepatocytes isolated from tdTomato mice challenged by SBT for 2 weeks. The hepatocytes were labeled by tdTomato (red) and anti-MANF (green) or anti-CK19 (green) antibody. **G**, **H** Effects of rhMANF on the protein levels of HNF4α, CK19, and MANF in primary hepatocytes isolated from tdTomato mice challenged by SBT for 2 weeks. The protein levels were detected by western blot assay. *n* = 6, *P < 0.05, **P < 0.01, ***P < 0.001. **I** The mRNA levels of HNF4α, CK19, and MANF in primary hepatocytes treated with rhMANF examined by qPCR. *n* = 6, *P < 0.05, **P < 0.01.

To investigate the effect of MANF on hepatocyte transformation into ICC cells, we crossed MANF^fl/fl^ mice with tdTomato^fl/fl^ mice and then injected AAV8-TBG-Cre via tail vein to construct mature hepatocyte-specific MANF KI/KO lineage tracing mice (Supplementary Fig. 7). By using these MANF KI/KO lineage tracing mice, ICC model was induced by SBT. After SBT injection, tdTomato^+^CK19^+^ cells appeared in the liver tissues, and the double-labeled cells were more in tdTomato MANF KI mice than tdTomato control mice (Fig. 6A, C). On the contrary, tdTomato^+^CK19^+^ cells were reduced in tdTomato MANF KO mice compared with tdTomato control mice after SBT induction (Fig. 6B, C). Meanwhile, we found many HNF4α^+^ CK19^+^ cells in TAA-induced ICC mice, and CK19 level was increased after rhMANF treatment. However, the number of HNF4α^+^ CK19^+^ cells was decreased in HKO mice, which was attenuated after rhMANF treatment in TAA-induced ICC mice (Fig. 6D). These results suggest that MANF drives mature hepatocytes to transform to ICC cells.

To verify this finding, we isolated the primary hepatocytes from tdTomato mice challenged with SBT for 2 weeks (Supplementary Fig. 8). The primary hepatocytes were treated with rhMANF at a concentration of 80 ng/mL. Obvious ductular-forming was found in tdTomato^+^ hepatocytes treated with rhMANF, compared with BSA-treated controls (Fig. 6E). Furthermore, rhMANF supplementation increased not only intracellular MANF, but also the number of tdTomato^+^CK19^+^ cells and the cytosolic CK19 level (Fig. 6F). We also found rhMANF treatment downregulated the HNF4α in the primary hepatocytes with or without SBT injection, while upregulated CK19 only after SBT exposure (Fig. 6G, H). The mRNA levels of HNF4α and CK19 in the primary hepatocytes were changed along with their protein levels. However, MANF mRNA level was obviously increased after SBT exposure but was reduced after rhMANF treatment (Fig. 6I). These data indicate that rhMANF acts as an exogenous inducer to promote mature hepatocytes lineage reprogramming to ICC cells.

### MANF detains CK19 in cytosol by binding to its Ser35 site

**Fig. 7:**
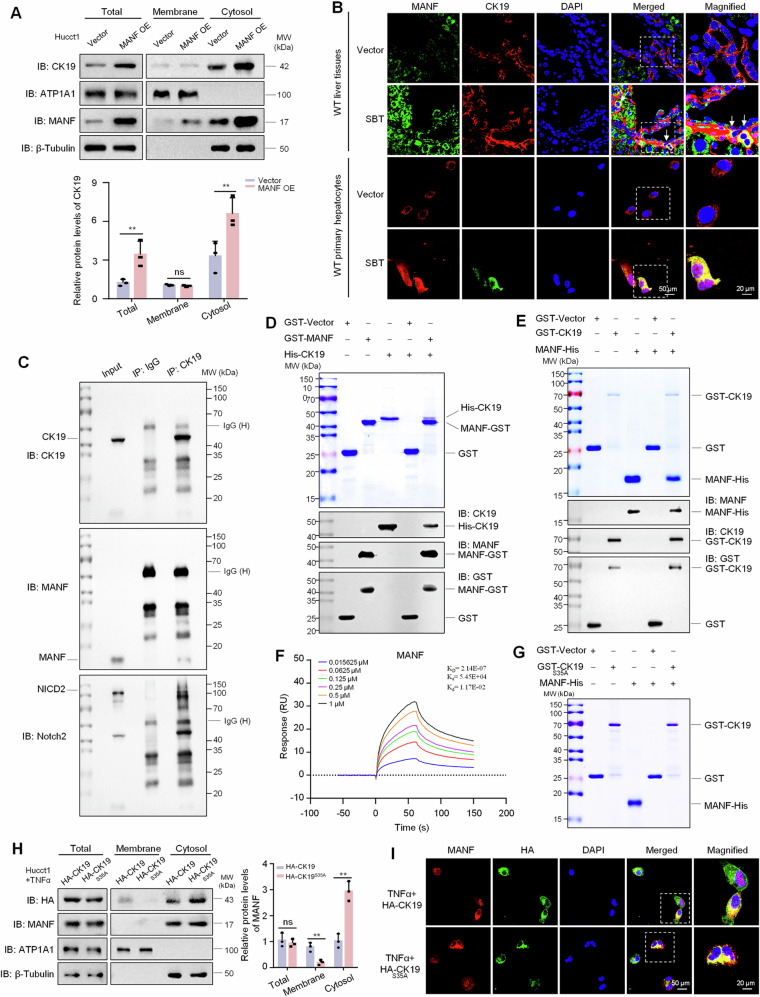
MANF detains CK19 in cytosol by binding to its Ser35 site. **A** The protein levels of CK19 and MANF in the membrane and cytosol of Hucct1 cells after MANF overexpression. *n* = 3, **P < 0.01. ATP1A1 and β-tubulin were used as the markers of cell membrane and cytosol, respectively. **B** The co-localization of MANF and CK19 in the liver tissues and primary hepatocytes of SBT-induced ICC mice. **C** Interaction of MANF, CK19, and NICD2 in Hucct1 cells detected by Co-IP assay. MANF and NICD2 were co-immunoprecipitated on the same PVDF membrane. The isotype IgG was used as a negative control. **D** His-CK19 was pulled down by MANF-GST. **E** MANF-His was pulled down by GST-CK19. **F** The affinity between MANF and CK19 was detected using Biacore assay. **G** The interaction of MANF and CK19 was canceled after CK19 mutation at Ser35 phosphorylation site. **H**, **I** CK19 mutation at Ser35 increases cytosolic CK19 in Hucct1 cells. The cells were transient transfected with CK19 and CK19^S35A^ plasmids and treated with TNFα (50 ng/mL). The proteins were detected by western blot (**H**, *n* = 3, **P < 0.01) and immunofluorescent staining (**I**) assays, respectively. ATP1A1 and β-tubulin were used as the markers of cell membrane and cytosol, respectively.

The localization of HNF4α and CK19 in TAA-induced mice ICC tissues in Fig. 6D make us more impressive. We observed that CK19 was mainly localized in cell membrane in HNF4α^+^CK19^+^ cells in TAA-induced ICC mice. After treatment with rhMANF, CK19 was mainly localized in the cytosol of the double-labeled cells. To investigate the effect of MANF on CK19 localization, we overexpressed MANF in Hucct1 cells and isolated the membrane and cytosol proteins. We found that both total CK19 and cytosolic CK19 were obviously increased after MANF overexpression, with CK19 primarily accumulating in the cytosol. More interesting, except for the increased cytosolic MANF, membrane-associated MANF was slightly increased in the cells overexpressing MANF, compared with the vector controls (Fig. 7A). Immunofluorescent double labeling of MANF and CK19 also revealed that the co-localization of MANF and CK19 was mainly in the cytosol in both liver tissues and primary hepatocytes (Fig. 7B). The interaction between MANF and CK19 was verified by Co-IP, GST-pull down, and Biacore assays (Fig. 7C–F). Considering the phosphorylation of CK19 at Ser35 is essential for its membrane recruitment [31, 32], we mutated CK19 at Ser35 into Ala35 (CK19^S35A^) and found that CK19 mutation canceled the interaction between MANF and CK19 (Fig. 7G). When we overexpressed HA-CK19^S35A^ in Hucct1 cells, CK19 level was decreased in the membrane, but increased in the cytosol, compared with the wild type (Fig. 7H, I). These results indicate that MANF inhibits the membrane translocation of CK19 by interacting with the Ser35 of CK19 in ICC cells.

### CK19 stabilizes NICD2 protein level to enhance its nuclear signaling

**Fig. 8:**
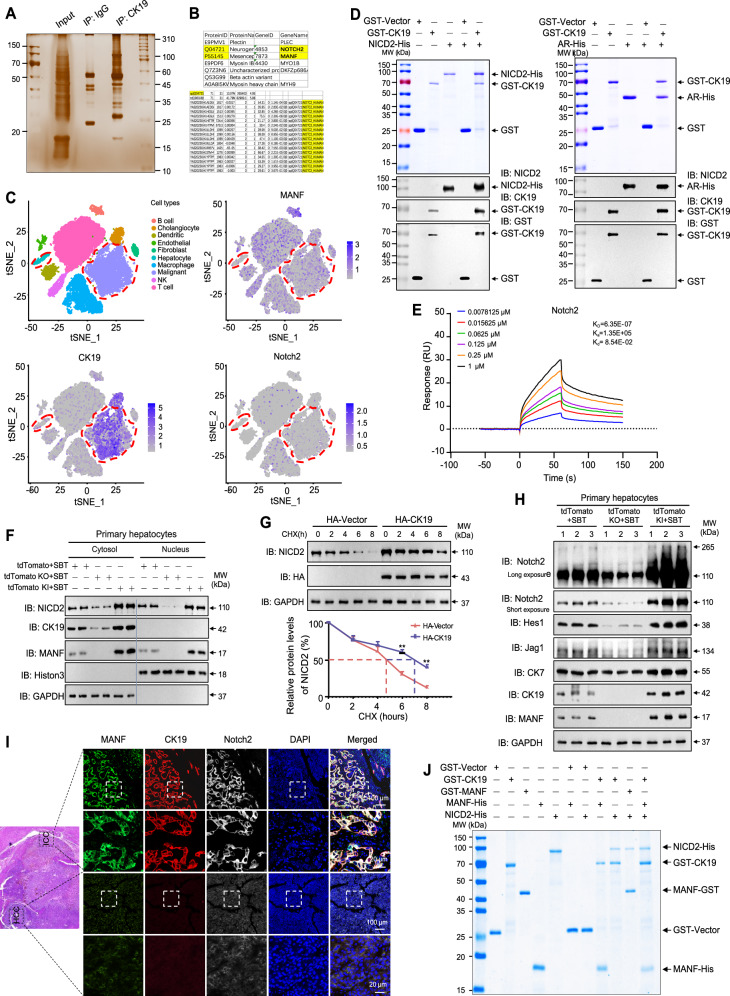
CK19 stabilizes NICD2 protein level to enhance its nuclear signaling. **A** Gel electrophoresis of the lysates stained by Silver after Co-IP with anti-CK19. Hucct1 cells were treated with TNFα (50 ng/mL) for 72 h before harvested. **B** Tryptic peptides of Notch2 identified by mass spectrometry in the immunoprecipitated complex by using an anti-CK19 antibody. **C** tSNE plots, color-coded for the expression (gray to blue) of marker genes for each cell type, as indicated (GSE138709). **D** The interaction of CK19 with NICD2 and AR detected by GST-pull down assays. **E** The affinity between CK19 and NICD2 was detected by Biacore assay. **F** Effects of MANF knockin/ knockout on the levels of NICD2 and CK19 in cytosol and nucleus of primary hepatocytes isolated from tdTomato mice challenged with SBT for 2 weeks. GAPDH and Histon 3 were used as the markers of cytosol and nucleus, respectively. **G** Effect of CK19 on the stability of NICD2 in Hucct1 cells transfected with HA-CK19 and treated with CHX (100 μg/mL). *n* = 3, **P < 0.01. **H** Effects of MANF knockin/knockout on the levels of Notch2, Hes1, Jag1 in primary hepatocytes isolated from tdTomato mice challenged with SBT for 2 weeks. **I** Comparison of the co-localization of MANF, CK19, and Notch2 in human ICC and HCC tissues detected by immunofluorescent staining with anti-MANF (green), anti-CK19 (red), and anti-Notch2 (white). **J** A complex composed of MANF, CK19, and NICD2 was identified by GST-pull down assay.

The next question is what’s the role of cytosolic CK19 in ICC. To answer this, we firstly screened CK19-interacting proteins using Co-IP plus mass spectrometry (MS). Notch2, a Notch signaling pathway receptor, was found as a candidate of CK19-interacting protein (Fig. 8A, B). Bioinformation analysis also showed that CK19 was associated with Notch2 (Supplementary Fig. 9A, B). By analyzing single-cell RNA sequencing data (GSE138709), we found that the expressions of MANF, CK19, and Notch2 signaling pathway genes were higher in malignant ICC cells than that in hepatocytes (Fig. 8C and Supplementary Fig. 9C). The co-localization of CK19 and Notch2 was found in the cytosol of primary hepatocytes (Supplementary Fig. 10A). The interaction between CK19 and Notch2 was verified by Co-IP, GST-pull down, and Biacore assays (Fig. 7C and Fig. 8D, E). To figure out the interacting domain, we constructed the truncates of Notch2, including the intracellular domains (NICD2) and its components (Supplementary Fig. 10B). Pull down assays showed that CK19 interacted with NICD2 and AR domains, but not RAM, TAD, and PEST domains (Fig. 8D and Supplementary Fig. 10C).

In the primary hepatocytes isolated from ICC mice treated with SBT for 2 weeks, we found that MANF knockout reduced cytosolic CK19. Meanwhile, MANF knockout reduced NICD2 in both cytosol and nucleus (Fig. 8F and Supplementary Fig. 10D). On the contrary, MANF overexpression increased cytosolic CK19 and NICD2, as well as nuclear NICD2 (Fig. 8F and Supplementary Fig. 10D). To know the effect of CK19 on the stability of NICD2, we performed cycloheximide (CHX) chase experiment in Hucct1 cells overexpressing HA-CK19. We found that CK19 overexpression significantly increased the stability and prolonged the half-life of Notch2 (Fig. 8G).

Apart from this, we detected the expression of Notch2 and its downstream molecules in primary hepatocytes isolated from SBT-induced ICC mice. Along with the increase of CK19 and Notch2, the levels of Hes1, Jag1, and CK7 were increased after MANF knockin while decreased after MANF knockout (Fig. 8H and Supplementary Fig. 10E). However, statistical difference was only observed in the mRNA level of Hes1 (Supplementary Fig. 10F).

To further determined the relationship among MANF, CK19, and Notch2 in ICC, we detected the expressions of MANF, CK19, and Notch2 in the liver tissues mixed with ICC and HCC by multiple immunofluorescent staining. The co-localization of MANF, CK19, and Notch2 was only observed in ICC, but not HCC (Fig. 8I). GST-pull down assay revealed a complex of MANF, CK19, and NICD2 (Fig. 8J), which suggests that MANF may act as a bridge to connect CK19 and NICD2. In addition, we also found that MANF and Notch2 were co-located in the nuclei of primary hepatocytes isolated from ICC mice challenged with SBT for 2 weeks (Supplementary Fig. 10G).

Notch1, the known intracellular domain NICD1 can be used to induce ICC model, was next investigated. The data revealed that higher Notch1 level coincided with higher MANF level, while lower Notch1 level accompanied by lower MANF level in ICC cell lines (Supplementary Fig. 11A, B). However, the interaction of MANF and Notch1 was not detectable by Co-IP assay (Supplementary Fig. 11C, D).

### MANF depends on CK19 and Notch2 to promote hepatocytes-derived ICC

**Fig. 9:**
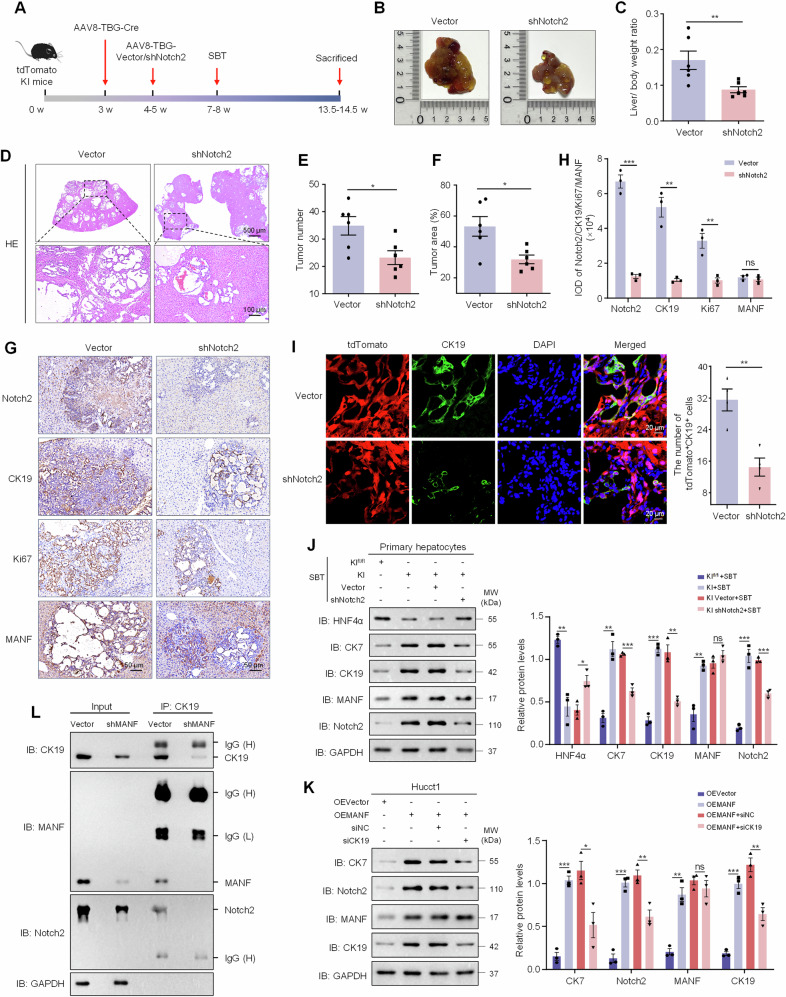
MANF depends on CK19 and Notch2 to promote hepatocytes-derived ICC. **A** Scheme for SBT-induced ICC after knockdown of Notch2 using AAVs. **B** Gross morphology of mice livers. **C** Liver-body weight ratio. *n* = 6, **P < 0.01. **D** HE staining of mice liver tissues. **E** Tumor number. *n* = 6, *P < 0.05. **F** Tumber area. *n* = 6, *P < 0.05. **G**, **H** The levels of Notch2, CK19, Ki67, and MANF in SBT-induced mice ICC after Notch2 knockdown detected by immunohistochemistry staining. *n* = 3, **P < 0.01, ***P < 0.001. **I** Effects of Notch2 knockdown on hepatocytes transformation in the liver tissues of SBT-induced ICC mice after MANF knockin. The hepatocytes were double labeled by tdTomato (red) and anti-CK19 (green) antibody. **J** Effects of Notch2 knockdown on the levels of HNF4α, CK7, CK19, MANF, and Notch2 in primary hepatocytes isolated from tdTomato MANF KI mice challenged with SBT for 2 weeks. *n* = 3, *P < 0.05, **P < 0.01, ***P < 0.001. **K** Effects of CK19 knockdown on the levels of CK7, Notch2, MANF, and CK19 in Hucct1 cells overexpressing MANF. *n* = 3, *P < 0.05, **P < 0.01, ***P < 0.001. **L** No interaction of MANF, CK19, and NICD2 in Hucct1 cells with MANF knockdown detected by Co-IP assay. The isotype IgG was used as a negative control.

To investigate whether NICD2 alone plays a key role in the transdifferentiation of hepatocytes into ICC cells regulated by MANF, we applied AAVs (AAV8-TBG-shVector/Notch2) to downregulate Notch2 in hepatocytes in tdTomato MANF KI mice (Supplementary Fig. 12A and Fig. 9A). After Notch2 silence, the livers were significantly smaller, the tumor number and area, and liver weight ratio were also significantly reduced following SBT treatment, compared with the control group (Fig. 9B–F). We also found that the upregulation of CK19, Ki67, CK7 and downregulation of HNF4α induced by MANF overexpression could be reversed by Notch2 knockdown (Fig. 9G, H, J). Importantly, lineage tracing results showed that the increase of tdTomato^+^CK19^+^ cells caused by MANF KI can be partially rescued by the silence of Notch2 (Fig. 9I), which fully indicates that the contribution of MANF to the transformation of hepatocytes into ICC cells is partially dependent on Notch2.

Also, the upregulation of Notch2 and CK7 induced by MANF overexpression can also be partially inhibited by silencing CK19 (Supplementary Fig. 12B and Fig. 9K). Meanwhile, we found that silencing MANF in Hucct1 cells reduced the interaction between CK19 and NICD2 (Fig. 9L), suggesting that MANF enhances CK19-NICD2 interaction. In turn, MANF enhanced NICD2 stability and Notch2 activation to promote the reprogramming of hepatocytes to ICC cells. These results indicate that the promoting effect of MANF on reprogramming of hepatocytes to ICC cells is partially dependent on CK19 and Notch2.

## Disscuion

In our study, we identified high expression of MANF in ICC tissues, which is related to the survival of ICC patients. Secondly, our data demonstrated that MANF plays an oncogenic role in ICC. Furthermore, we found MANF promotes the transformation of mature hepatocytes into cholangiocyte-like cells in SBT and TAA-induced ICC by using the fluorescent reporter mice. The underlying mechanisms include that MANF physically binds to Ser35 of CK19 to inhibit CK19 membrane translocation, MANF facilitates the formation of a complex with CK19 and NICD2, and cytosolic CK19 interacts with AR domain of NICD2 to suppress NICD2 degradation and activate its nuclear signaling, thus accelerating the transformation of mature hepatocytes to ICC cells.

Our previous study found that MANF was lowered in HCC tissues and played a suppressive role in HCC [30]. However, this study shows that MANF was highly expressed in ICC tissues, while there was no significant change in MANF level in extrahepatic cholangiocarcinoma, although they are primary carcinoma of the liver.

HCC and ICC, both belong to primary liver cancer, often share similarities in pathogenesis and clinical characteristics, which makes difficulties in diagnosing ICC and HCC [33]. Pathological detection of AFP and CA19-9 is still the gold standard to distinguish ICC and HCC. However, the diagnostic specificity and sensitivity of these biomarkers remain unsatisfactory [34]. Our results suggest that MANF could serve as a novel biomarker for distinguishing ICC and HCC and predicting ICC tumor stage. As a secreted protein, MANF can be easily detected in serum, highlighting its potential as a non-invasive biomarker for the early diagnosis of ICC.

Small molecule inhibitors are widely used in anticancer therapy by targeting oncogenic proteins. In this study, we demonstrated that MANF plays a carcinogenic role in ICC, which makes MANF a potential therapeutic target for ICC. Abnormal expression of MANF may contribute to ICC pathogenesis. Screening small molecules targeting MANF will provide insights for the treatment of ICC.

Hucct1 and RBE are commonly used ICC cell lines. Cancer cells in primary and metastatic tumors exhibit significant biological heterogeneity, complicating the treatment of metastatic tumors [35]. Hucct1 cells were derived from the ascites of male ICC patient with moderately differentiated, metastasis, and gene mutations such as KRAS, TP53, and MSH6, while the RBE cells were isolated from the primary tumor of a female ICC patient with IDH1 and KRAS mutations [36]. Studies have shown that abnormal expression of KRAS and TP53 contributes to a more aggressive ICC phenotype [3]. Metastasis is the leading cause of mortality in ICC patients. However, little is known about its molecular mechanisms. Our study revealed that MANF was highly expressed in Hucct1 cells, other than in RBE cells, and MANF overexpression or knockdown had a more pronounced effect on Hucct1 cells than RBE cells, suggesting that MANF may be an effective marker for predicting ICC metastasis.

It has been reported that MANF was highly expressed in human ICC tissues and predicted poor prognosis [37], which is consistent with our findings. However, this group also reported that MANF level was increased in HCC [38]. Here, we found that MANF overexpression strongly promoted proliferation and invasion in ICC cell lines, a phenotype not observed in the previous study [37]. This difference may stem from differences in experimental approaches. We used lentivirus to construct the stable cell lines overexpressing MANF, ensuring higher transfection efficiency and repeatability, whereas the previous study adopted transient transfection with FLAG-MANF plasmid, [37]. They did not observe the effect of MANF knockdown on the tumor growth in nude mice injected with Hucct1 within four weeks [37]. However, we did find that MANF knockdown dramatically inhibited tumorigenesis and tumor growth in nude mice subcutaneously injected with Hucct1 cells stably knocked down MANF in the ninth week. The detailed reasons for these discrepancies remain unclear due to limited methodological details provided in their article.

To achieve the aim of specifically knocking out MANF in hepatocytes, we used both AAV8-TBG-Cre and Alb-Cre tool mice in this study, as each has distinct limitations. Although AAV8-TBG-Cre has the advantage of specificity for mature hepatocytes, AAV8-TBG-Cre cannot take the place of Alb-Cre due to short lasting time and multiple injections in TAA-induced ICC. Alb promoter has a relatively broad pertinence, not only in hepatocytes but also in HPCs, the latter can transdifferentiate into BECs or hepatocytes during the development stage of mice or under emergency conditions. Therefore, strictly speaking, Alb-Cre is not suitable for lineage tracing.

ICC has been divided into 4 distinct proteomic subgroups in the previous literature [3]. In our study we found that CK19 was remarkably increased after SBT treatment, suggesting that SBT-induced ICC may belong to subgroup 4 which characterized by the expression of biliary tract-specific proteins. Furthermore, our data reveal that the MANF overexpression facilitated the transformation of mature hepatocytes into ICC cells, and the hepatocytes displayed mesenchymal-like morphology in MANF KI mice after SBT induction. Meanwhile, MANF overexpression increased the levels of mesenchymal markers, while decreased the levels of epithelial markers, indicating that MANF may also promote ICC via inducing EMT. TAA-induced ICC shows obvious inflammatory infiltration and pseudolobule formation. TAA induces the proliferation of BECs and facilitates the transformation of hepatocytes into ICC, which is similar to SBT-induced ICC.

Hepatocytes, account for 80% of liver cells, work as the source of liver cells to maintain liver homeostasis after liver injury. It can be transformed to other cell types in response to the change of microenvironment. For example, hepatocytes can transform into ICC cells directly or dedifferentiate into HPCs [4, 7, 39]. Hepatocytes can also transdifferentiate into BECs, and further deteriorate into ICC cells within the appropriate tumor microenvironment [40]. For this reason, dynamic observation of hepatocytes development is required for accurately determining the lineage reprogramming mode of hepatocytes in ICC. Here, we used lineage tracing technique to explore the lineage reprogramming of hepatocytes in SBT-induced ICC, in which hepatocytes were not only transformed into ICC cells, but also transformed into tdTomato^+^α-SMA^+^ and tdTomato^+^CD133^+^ cells, suggesting that SBT induces hepatocytes to evolve to the multifunctional cells. It was reported that ICC originated from mature hepatocytes in SBT-treated mice [41]. HPCs were also mentioned to originate from hepatocytes in liver injury models [7]. However, whether the hepatocytes were transformed into myofibroblasts remains unclear. Our study reported for the first time that SBT drove hepatocytes to transform into tdTomato^+^α-SMA^+^ cells, suggesting that EMT may be induced in SBT-induced ICC. The appearance of tdTomato^+^CD133^+^ cells highlights the plasticity and diversity of hepatocytes in the process of ICC development.

Hepatocytes can be malignantly transformed into HCC and ICC simultaneously, the two major types of the primary liver cancers, in different microenvironments [40]. What reasons or mechanisms control the transformation of hepatocytes into different types? Our results demonstrated that hepatocyte-derived MANF promotes the reprogramming of hepatocytes into ICC cells and our previous study also showed MANF inhibited the malignant transformation of hepatocytes into HCC [30], which suggests that MANF may have an important role in lineage selection of hepatocytes. Therefore, MANF can be considered as a new inducer or suppressor in the transformation of hepatocytes into ICC and HCC, respectively.

HNF4α, a nuclear molecule, plays an important role in maintaining the function of mature hepatocytes [42, 43]. Recombinant MANF treatment decreased HNF4α level with or without SBT induction, which may attenuate the effect of HNF4α on the mature hepatocytes and may be benefit for the transformation of mature hepatocytes into BECs or ICC cells. It has been reported that necrotic tumor microenvironment drives hepatocytes to transform into ICC [40]. Actually, MANF needs to cooperate with other molecules or environment in promoting ICC, which was supported by the findings that MANF KI alone neither induced ICC nor exert an effect on hepatocytes transformation.

CK19, the smallest class I cytoplasmic intermediate filament protein, belongs to the keratin superfamily of filament proteins of epithelial cells [44] with assembly properties [45, 46]. Keratins have asymmetric distribution and highly concentrated under the apical domain [47]. CK19 has been recognized as a biomarker of BECs, HPCs, and tumor stem cells [48–50]. However, CK19 re-emerges on hepatocytes when the normal livers are challenged with inflammation or other injury stimuli [13, 39, 51], and CK19^+^ HCC was demonstrated aggressive behaviors [52–54]. The previous study showed it was externalized in several human cancer cell lines [55]. In our study, we observed MANF colocalized with CK19 in the cytoplasm of primarily cultured hepatocytes and hepatic cells in ICC. We also verified the physical interaction of MANF and CK19 in vitro. Since MANF is a soluble protein and can enter into cytosol, we suppose that the interaction of MANF and CK19 may happen in cytosol. Our results showed that MANF increased the level of cytosolic CK19. There is a strong correlation between accumulation of hepatotoxicity and a significant increase of CKs phosphorylation [56]. Ser35 is the major site for CK19 phosphorylation [31, 56], which is required for CK19 recruitment to the membrane [32]. Interestingly, we found a mutation at CK19 Ser35 site canceled the interaction of MANF and CK19, which indicates Ser35 is the binding site of MANF and CK19.

Keratins play critical roles in maintaining epithelial barriers, facilitating intracellular signaling conduction, and supporting differentiation [47, 50]. Recent research showed that CK19 directly interacted with β-catenin-RAC1 complex and stabilizing the ubiquitination and proteasomal degradation of β-catenin in breast cancer [57]. The PDGFRα-LAMB1 pathway supported tumor progression at the invasive front of human HCC through CK19 expression [52]. However, the role of CK19 in ICC beyond its function as a biomarker remains poorly understood. In this study, we found CK19 interacted with NICD2 and maintained NICD2 stability. Notch2 signaling pathway is an evolutionarily conservative pathway in multicellular organisms and regulating the fate of hepatocytes [18, 19, 58]. NICD2 is released and enters to cytosol after Notch2 signal pathway is activated [19]. NICD2 undergoes nuclear translocation and promotes the activation of its downstream targets. On the other hand, cytosolic NICD2 will be phosphorylated, ubiquitinated, and further degraded by proteasome [20]. It was reported that intermediate filaments is involved in Notch2 signal transmission [45]. As insoluble structures, intermediate filaments provide a solid surface to bind and immobilize proteins, referred to as “scaffolding” [47]. Intermediate filament scaffolding sequesters several proteins in the position where they should perform their functions [47]. CK19 was found to interact with AR domain of NICD2 and form a complex with MANF and CK19 in our study. It was found that CK19 stabilized HER2 to facilitate the effect of HER2 on breast cancer [31]. Our results also showed that CK19 stabilized cytosolic NICD2 level and prevent NICD2 degradation.

**Fig. 10:**
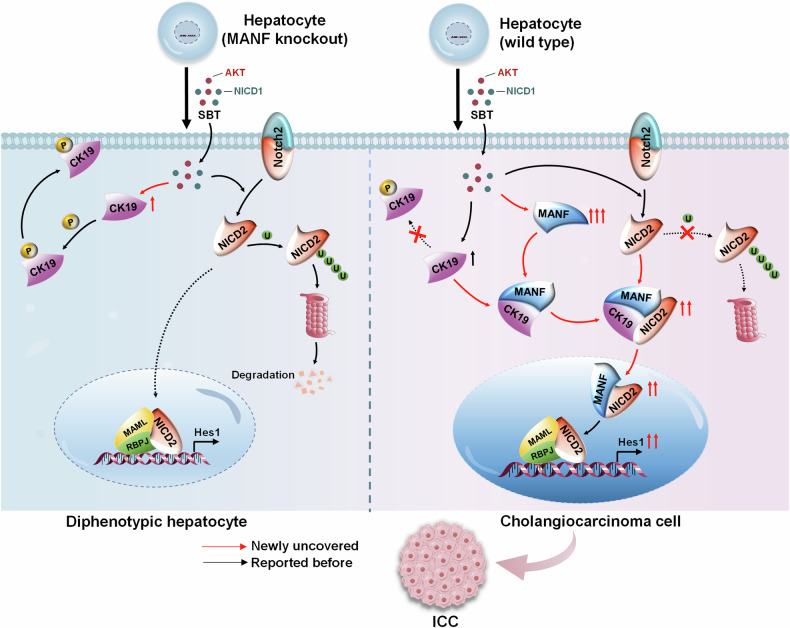
Schematic diagram of the role of MANF in promoting ICC. Mature hepatocytes become biphenotypic cells after SBT challenge. Hepatocytes lacking MANF cannot transdifferentiate into ICC cells, while the hepatocytes rich in MANF can transform into ICC cells successfully. In detail, intracellular MANF interacts with CK19 at the site of Ser35 to suppress CK19 recruitment to the membrane. As a result, CK19 is detained in the cytosol where it binds to AR domain within NICD2 to stabilize cytosolic NICD2 and enhance nuclear Notch2 signaling, which contributes to hepatocytes transformation to ICC cells.

Taking together, our study demonstrates that MANF inhibits the membrane translocation of CK19 to enhance the cytosolic interaction of CK19 and NICD2, and subsequently accelerates Notch2 signaling activation, which contributes to hepatocyte lineage reprogramming to ICC cells. Notably, the treachery of MANF, an initial liver protective factor, in ICC is a representative regulatory mechanism of body, which opens the doors for potential diagnosis and treatment of ICC (Fig. 10).

## Methods and materials

### Human samples

Human liver tissues paraffin sections from 78 ICC patients, 4 mixed HCC-ICC liver cancer patients, 6 extrahepatic cholangiocarcinoma tissues paraffin sections, the frozen liver tissues from 6 ICC patients, and the serum from 23 ICC patients and 25 healthy individuals were collected from the First Affiliated Hospital of Anhui Medical University (Hefei, China). The corresponding Pa tissues were collected as the controls. The clinical pathological features of ICC were presented in Supplementary Table 1.

### Mice

The 6 to 8-week wild type (WT) mice on a C57BL/6J background, Rosa26-CAG-LSL-Cas9-tdTomato (tdTomato^fl/fl^, T002249), MANF^fl/fl^ (for KI), MANF^fl/fl^ (for KO), Alb-Cre, and 6-week male nude mice (BALB/c background) were purchased from GemPharmatech Co., Ltd (Nanjing, China). All animals were maintained under special pathogen-free (SPF) conditions at a temperature of 20–23 °C on a 12-h light-dark cycle.

#### tdTomato^fl/fl^ mice

tdTomato^fl/fl^ male mice were used for hepatocytes lineage tracing. Cre/loxP-mediated removal of the STOP element is required to activate tdTomato expression.

#### Knockin MANF^fl/fl^; TBG-Cre mice and Knockout MANF^fl/fl^; TBG-Cre mice

MANF KI and KO mice were generated by CRISPR/Cas9 technology (Fig. 3A, I). The CDS sequence of MANF was inserted at H11 site to construct MANF KI mice, while exon 3 of MANF gene was deleted to achieve MANF KO.

#### Knockout MANF^fl/fl^; Alb-Cre mice

MANF^fl/fl^ mice were cross-bred with Alb-Cre mice to get the hepatic MANF knockout (HKO) mice (Supplementary Fig. 6K–N).

#### tdTomato^fl/fl^; Knockin MANF^fl/fl^; TBG-Cre mice and tdTomato^fl/fl^; Knockout MANF^fl/fl^; TBG-Cre mice

MANF^fl/fl^ (KI and KO) mice were crossed with tdTomato^fl/fl^ mice to construct mature hepatocyte-specific MANF KI or KO lineage tracing mice after AAV8-TBG-Cre tail vein injection (Supplementary Fig. 7).

### Mice ICC model

#### Sleeping beauty transposon (SBT)-induced ICC

Short-term mice ICC was induced in 8-week-old male mice via hydrodynamic tail vein injection (HTVI) of pT3 plasmids to overexpress Myc-tagged mouse Notch1 receptor NICD1 and HA-tagged human myrAKT1, along with a hyperactive sleeping beauty transposase plasmid (purchased from Haixing Biosciences), as previously described [59]. Briefly, system contained 25 μg HA-AKT, 25 μg Myc-NICD1, and 4 μg transferase diluted in 2 mL 0.9% NaCl and injected into tail vein within 6–8 s for 6.5 weeks (Fig. 1E).

#### TAA-induced ICC

Eight-week-old male mice were provided drinking water containing TAA (600 mg/L, Sigma, 163678) for 32 weeks to induce long-term mice ICC (Fig. 1J).

#### Tumor subcutaneous xenograft assay

Six-week-old male nude mice (BALB/c background) were subcutaneously injected with 5 × 10^6^ Hucct1 cells into the right flanks for 9 weeks. The maximal and average volume of the tumors were calculated (length × width^2^ × 0.5).

### Plasmids and antibodies

Sleeping beauty transposon plasmids, NICD2-His, RAM-His, AR-His, TAD-His, and PEST-His prokaryotic plasmids were purchased from Haixing Biosciences (Suzhou, China). MANF-GST and MANF-His prokaryotic plasmids were constructed by us and retained in our laboratory [60]. GST-CK19, GST-CK19^S35A^, His-CK19 prokaryotic plasmids, and HA-CK19, HA-CK19^S35A^ eukaryotic plasmids were purchased from Songon Biotech (Shanghai, China). Lentiviral transduction particles containing MANF DNA (MANF-Flag, MANF OE) and short hairpin RNA (shMANF) were purchased from Tsingke BiotechnologyCo., Ltd (Nanjing, China). The siRNAs targeting Notch2 and CK19 were purchased from Hanbio Tech (Shanghai, China) and Songon Biotech (Shanghai, China), respectively. The sequences of Notch2-siRNA and CK19-siRNA used to silence Notch2 and CK19 in AML12 and Hucct1 cells are presented in Supplementary Table 2. The used antibodies are listed in Supplementary Table 3.

### AAVs injection

The AAV8-TBG-Cre and AAV8-TBG-shNotch2 viruses were packaged and purified by Hanbio Tech (Shanghai, China) to specifically control Cre expression and knockdown Notch2 in mature hepatocytes, respectively. Mice aged 3–4 weeks were injected with AAVs by tail vein at 1.3 × 10^11^ genome copies per mouse. After AAVs took effect, 6–8-week-old mice were used to construct SBT-induced mice ICC.

### Serum biochemical index detection

Serum ALT, AST, TBIL, and DBIL levels were detected by fully automatic biochemical analyzer (Rili, Japan) according to the manufacturer’s instructions. In TAA model, two blood samples were excluded due to hemolysis. Serum MANF levels were measured using ELISA kits according to the manufacturer’s instructions (Reed Biotech, RE1283H-96T).

### Immunohistochemistry

Immunohistochemistry assay was performed as previously described [60]. Paraffin sections were deparaffinized, performed an antigen retrieval, incubated with antibodies, then stained with 3, 3’-diaminobenzidine (DAB, ZLI-9018, ZSGB Bio) and hematoxylin. The images were obtained with an intelligent section imaging analysis system (3D HISTECH). Image J software was used to measure the integral optical density (IOD).

### Multiplex immunohistochemical

Four-color multiple fluorescent immunohistochemical staining kit (RS0035, ImmunoWay Biotechnology) was used based on the tyramide signal amplification technique following the manufacturer’s protocol. The images were collected with laser confocal (Zeiss, LSM800).

### Immunofluorescence

Fresh liver tissues were embedded and sectioned for 8 μm after fixing in 4% paraformaldehyde and dehydrating by 30% sucrose. The corresponding antibodies and DAPI were incubated after blocking. The images were collected by microscope (Olympus) and laser confocal (Zeiss, LSM800).

### Western blot

Proteins were extracted using RIPA lysis buffer containing protease inhibitor. Equal amounts of protein were loaded on SDS-PAGE gels and probed with antibodies. Blots were visualized using a chemiluminescence system (Bio-Rad, Hercules, USA).

### Quantitative real-time fluorescence PCR (qPCR)

Total RNAs were extracted using the RNA extraction kit (AG, AG21017 and AG21023) according to the instructions. The Reverse Transcription Kit (AG, AG11605) was used to reverse RNA to cDNA. QPCR assays were carried out using SYBR Green PCR Master Mix (TOYOBO, 857100). GAPDH served as the internal control. The primer sequences involved were included in Supplementary Table 4.

### Cell lines

Hucct1, RBE, and AML12 cell lines were purchased from Procell with short tandem repeat (STR) identification and cultured in RPMI 1640 (Hucct1 and RBE) with 10% fetal bovine serum or DMEM/F12 (AML12) with 10% fetal bovine serum, 1×ITS-G, 40 ng/mL dexamethasone in incubator (37 °C, 5% CO_2_). Plasmids were transfected using lipofectamine^TM^ 3000 Reagent (Invitrogen, L3000015) according to the manufacturer’s instructions. The stably transfected cholangiocarcinoma cells were selected with 2 μg/mL puromycin (Beyotime, ST551).

### Malignant biological behavior assays

#### Colony-formation assay

Cells were seeded with at a density of 400 cells/well in 6-well plates and incubated for 2 weeks. Colonies were stained with 0.1% crystal violet, and images were captured to count colony numbers.

#### Transwell invasion assay

Fifty thousand cells suspended in serum-free RPMI 1640 were seeded into matrigel-coated filters in the upper chambers of 24-well Matrigel Invasion Chambers (Corning, USA). RPMI 1640 containing 15% fetal bovine serum was placed to the lower chamber as an attractant. Thirty-six hours later, the cells were stained with 0.1% crystal violet. The images were obtained with microscope (Olympus).

### Isolation of primary hepatocytes

Mice were anesthetized and perfused with prewarmed 37 °C Hank’s buffer (Beyotime, C0218) and 0.05% collagenase IV (Sigma, C-5138) via hepatic portal vein. The isolated livers were digested in 0.05% collagenase IV at 37 °C for 15 min, then terminated with DMEM containing 10% fetal bovine serum. The cell suspension was filtered through a 200-mesh strainer, followed by gradient centrifugation and erythrocyte lysis. Isolated hepatocytes were seeded in plates and cultured in DMEM containing 10% fetal bovine serum at 37 °C with 5% CO_2_.

### Subcellular separation assays

The specialized cellular component isolation kits were used to separate membrane-cytosol (Invent Biotechnologies, SM005) and nucleus-cytosol (Invent Biotechnologies, NT032) proteins in accordance with the manufacturer’s instructions.

### Co-Immunoprecipitation (Co-IP)

Co-IP assay was performed as previously reported [30, 60]. Briefly, protein A/G magnetic beads were pre-incubated with specific antibodies, followed by incubation with tissue or cell lysis. The indicated bound proteins were detected using western blot.

### GST-pull down assay

MANF-GST and MANF-His prokaryotic plasmids were constructed by us and kept in our laboratory [60]. All prokaryotic plasmids were induced by IPTG to produce protein. His-tag protein was incubated with Pierce Glutathione Agarose (16100, Thermo Scientific) bearing immobilized GST-tag fusion protein at 4 °C for 2 h. The pulled down proteins were detected using Coomassie and western blot.

### Biacore assay

Biacore 8 K (Biacore, GE Healthcare) equipped with a CM5 Sensor Chip (Cytiva, BR-1005-30) was used. The ligand protein CK19 was diluted to 50 μg/mL with sodium acetate and immobilized on the CM5 chip at a flow rate of 10 μL/min. MANF and NICD2 were used as analytes. The running buffer for the compounds was chosen to be 1× PBS-P+ containing 5% DMSO (PH 7.4). Compounds were serially diluted and injected into the chip in ascending concentrations. The flow rate was 30 μL/min and the duration was 150 s. The chip was regenerated with 10 mM glycine hydrochloride solution (pH 2.0) for 5 min between runs.

### Bioinformatic analysis and data acquisition

The corresponding clinical information and RNA-seq data were obtained from TCGA, GSE179443, GSE107943, GSE241923, GSE221589, and GSE138709 databases. Patients with ICC were divided into MANF low-expression and high-expression groups based on the median MANF expression. The ICC cohort from TCGA was used for co-expression analysis of CK19 and Notch2 using the LinkedOmics. For correlation analysis, genes with |cor | > 0.4 and adjusted FDR < 0.05 in the Pearson correlation test were considered.

### Statistical analysis

Statistical analysis was performed with GraphPad Prism 8.0 and SPSS 25.0 software. Samples were selected randomly within each group. All data were expressed as mean ± SEM. Statistical significance between two experimental groups was determined by paired or unpaired Student’s *t* test. Multiple groups were compared with ANOVA. The chi-squared (*χ*2) test was used to evaluate the correlation between MANF expression and clinical pathological features. *P* < 0.05 was considered as statistical significance.

## Supplementary information

Supplementary Materials

Original Data Files

## Data Availability

The data that contribute to the findings of this study are available within the article and included in Supplementary files. The MS proteomics data have been deposited to the ProteomeXchange Consortium via the PRIDE partner repository with the dataset identifier PXD048451 (Username: reviewer_pxd048451@ebi.ac.uk and Password: NHg1JLZm). Further data supporting the findings of this study are available from the corresponding author upon reasonable request. Restrictions apply to the availability of individual participant data. Source data are provided with this paper.

## References

[1] Rodrigues PM, Olaizola P, Paiva NA, Olaizola I, Agirre-Lizaso A, Landa A, et al. Pathogenesis of cholangiocarcinoma. Annu Rev Pathol-Mech. 2020;16:433–63. 10.1146/annurev-pathol-030220-020455 33264573

[2] Rizvi S, Gores GJ. Pathogenesis, diagnosis, and management of cholangiocarcinoma. Gastroenterology. 2013;145:1215–29. 24140396 10.1053/j.gastro.2013.10.013 PMC3862291

[3] Dong L, Lu D, Chen R, Lin Y, Zhu H, Zhang Z, et al. Proteogenomic characterization identifies clinically relevant subgroups of intrahepatic cholangiocarcinoma. Cancer Cell. 2022;40:70–87. 34971568 10.1016/j.ccell.2021.12.006

[4] Zhu Y, Kwong LN. Insights into the origin of intrahepatic cholangiocarcinoma from mouse models. Hepatology. 2020;72:305–14. 32096245 10.1002/hep.31200

[5] He L, Pu W, Liu X, Zhang Z, Han M, Li Y, et al. Proliferation tracing reveals regional hepatocyte generation in liver homeostasis and repair. Science. 2021;371:eabc4346. 33632818 10.1126/science.abc4346

[6] Malato Y, Naqvi S, Schürmann N, Ng R, Wang B, Zape J, et al. Fate tracing of mature hepatocytes in mouse liver homeostasis and regeneration. J Clin Investig. 2011;121:4850–60. 22105172 10.1172/JCI59261 PMC3226005

[7] Wei M, Lü L, Lin P, Chen Z, Quan Z, Tang Z. Multiple cellular origins and molecular evolution of intrahepatic cholangiocarcinoma. Cancer Lett. 2016;2:253–61. 10.1016/j.canlet.2016.02.038 26940139

[8] Hu S, Molina L, Tao J, Liu S, Hassan M, Singh S, et al. NOTCH-YAP1/TEAD-DNMT1 axis drives hepatocyte reprogramming into intrahepatic cholangiocarcinoma. Gastroenterology. 2022;163:449–65. 35550144 10.1053/j.gastro.2022.05.007 PMC9329208

[9] Liu Y, Zhuo S, Zhou Y, Ma L, Sun Z, Wu X, et al. Yap-Sox9 signaling determines hepatocyte plasticity and lineage-specific hepatocarcinogenesis. J Hepatol. 2022;76:652–64. 34793870 10.1016/j.jhep.2021.11.010 PMC8858854

[10] Wang J, Wang H, Peters M, Ding N, Ribback S, Utpatel K, et al. Loss of Fbxw7 synergizes with activated Akt signaling to promote c-Myc dependent cholangiocarcinogenesis. J Hepatol. 2019;71:742–52. 31195063 10.1016/j.jhep.2019.05.027 PMC6773530

[11] Fingas CD, Mertens JC, Razumilava N, Sydor S, Bronk SF, Christensen JD, et al. Polo-like kinase 2 is a mediator of hedgehog survival signaling in cholangiocarcinoma. Hepatology. 2013;58:1362–74. 23703673 10.1002/hep.26484 PMC3811036

[12] Razumilava N, Gradilone SA, Smoot RL, Mertens JC, Bronk SF, Sirica AE, et al. Non-canonical Hedgehog signaling contributes to chemotaxis in cholangiocarcinoma. J Hepatol. 2014;60:599–605. 24239776 10.1016/j.jhep.2013.11.005 PMC3944428

[13] Han X, Wang Y, Pu W, Huang X, Qiu L, Li Y, et al. Lineage tracing reveals the bipotency of SOX9^+^ hepatocytes during liver regeneration. Stem Cell Rep. 2019;12:624–38. 10.1016/j.stemcr.2019.01.010 PMC6409431 30773487

[14] Marquardt JU, Andersen JB, Thorgeirsson SS. Functional and genetic deconstruction of the cellular origin in liver cancer. Nat Rev Cancer. 2015;15:653–67. 26493646 10.1038/nrc4017

[15] D’Artista L, Moschopoulou AA, Barozzi I, Craig AJ, Seehawer M, Herrmann L, et al. MYC determines lineage commitment in KRAS-driven primary liver cancer development. J Hepatol. 2023;79:141–9. 36906109 10.1016/j.jhep.2023.02.039 PMC10330789

[16] Carotenuto P, Fassan M, Pandolfo R, Lampis A, Vicentini C, Cascione L, et al. Wnt signalling modulates transcribed-ultraconserved regions in hepatobiliary cancers. Gut. 2017;66:1268–77. 27618837 10.1136/gutjnl-2016-312278 PMC5530482

[17] Perugorria MJ, Olaizola P, Labiano I, Esparza-Baquer A, Marzioni M, Marin JJG, et al. Wnt–β-catenin signalling in liver development, health and disease. Nat Rev Gastro Hepat. 2019;16:121–36. 10.1038/s41575-018-0075-9 30451972

[18] Jeliazkova P, Jörs S, Lee M, Zimber-Strobl U, Ferrer J, Schmid RM, et al. Canonical Notch2 signaling determines biliary cell fates of embryonic hepatoblasts and adult hepatocytes independent of Hes1. Hepatology. 2013;57:2469–79. 23315998 10.1002/hep.26254

[19] Wang J, Dong M, Xu Z, Song X, Zhang S, Qiao Y, et al. Notch2 controls hepatocyte-derived cholangiocarcinoma formation in mice. Oncogene. 2018;37:3229–42. 29545603 10.1038/s41388-018-0188-1 PMC6002343

[20] Zhu P, Wang Y, Du Y, He L, Huang G, Zhang G, et al. C8orf4 negatively regulates self-renewal of liver cancer stem cells via suppression of NOTCH2 signalling. Nat Commun. 2015;6:7122. 25985737 10.1038/ncomms8122 PMC4479000

[21] Zender S, Nickeleit I, Wuestefeld T, Sörensen I, Dauch D, Bozko P, et al. A critical role for Notch signaling in the formation of cholangiocellular carcinomas. Cancer Cell. 2013;23:784–95. 23727022 10.1016/j.ccr.2013.04.019

[22] Li QM, Li X, Su SQ, Wang YT, Xu T, Zha XQ, et al. Dendrobine inhibits dopaminergic neuron apoptosis via MANF-mediated ER stress suppression in MPTP/MPP^+^-induced Parkinson’s disease models. Phytomedicine. 2022;102:154193. 35636177 10.1016/j.phymed.2022.154193

[23] Xu S, Di Z, He Y, Wang R, Ma Y, Sun R, et al. Mesencephalic astrocyte-derived neurotrophic factor (MANF) protects against Aβ toxicity via attenuating Aβ-induced endoplasmic reticulum stress. J Neuroinflamm. 2019;16:35. 10.1186/s12974-019-1429-0 PMC6373169 30760285

[24] Yu YQ, Liu LC, Wang FC, Liang Y, Cha DQ, Zhang JJ, et al. Induction profile of MANF/ARMET by cerebral ischemia and its implication for neuron protection. J Cerebr Blood F Met. 2010;30:79–91. 10.1038/jcbfm.2009.181 PMC2949098 19773801

[25] Lindahl M, Danilova T, Palm E, Lindholm P, Võikar V, Hakonen E, et al. MANF is indispensable for the proliferation and survival of pancreatic β cells. Cell Rep. 2014;7:366–75. 24726366 10.1016/j.celrep.2014.03.023 PMC7254957

[26] Yang Y, Wang P, Zhang C, Huang F, Pang G, Wei C, et al. Hepatocyte‐derived MANF alleviates hepatic ischaemia‐reperfusion injury via regulating endoplasmic reticulum stress‐induced apoptosis in mice. Liver Int. 2020;41:623–39. 33064897 10.1111/liv.14697

[27] Wang P, Yang Y, Pang G, Zhang C, Wei C, Tao X, et al. Hepatocyte-derived MANF is protective for rifampicin-induced cholestatic hepatic injury via inhibiting ATF4-CHOP signal activation. Free Radical Bio Med. 2021;162:283–97. 33127565 10.1016/j.freeradbiomed.2020.10.028

[28] Xu H, Jiao Y, Li S, Zhu X, Wang S, Zhang Y, et al. Hepatocyte-derived MANF mitigates ethanol-induced liver steatosis in mice via enhancing ASS1 activity and activating AMPK pathway. Acta Pharmacol Sin. 2023;44:157–68. 35655095 10.1038/s41401-022-00920-8 PMC9813016

[29] Hou C, Wang D, Zhao M, Ballar P, Zhang X, Mei Q, et al. MANF brakes TLR4 signaling by competitively binding S100A8 with S100A9 to regulate macrophage phenotypes in hepatic fibrosis. Acta Pharm Sin B. 2023;13:4234–52. 37799387 10.1016/j.apsb.2023.07.027 PMC10547964

[30] Liu J, Wu Z, Han D, Wei C, Liang Y, Jiang T, et al. Mesencephalic astrocyte‐derived neurotrophic factor inhibits liver cancer through small ubiquitin‐related modifier (SUMO)ylation‐related suppression of NF‐κB/Snail signaling pathway and epithelial‐mesenchymal transition. Hepatology. 2020;71:1262–78. 31469428 10.1002/hep.30917 PMC7187412

[31] Ju JH, Oh S, Lee K, Yang W, Nam KS, Moon HG, et al. Cytokeratin19 induced by HER2/ERK binds and stabilizes HER2 on cell membranes. Cell Death Differ. 2015;22:665–76. 25342465 10.1038/cdd.2014.155 PMC4356337

[32] Zhang X, Xu X, Zhang Z, Xue C, Kong Z, Wu S, et al. Linc-KILH potentiates Notch1 signaling through inhibiting KRT19 phosphorylation and promotes the malignancy of hepatocellular carcinoma. Int J Biol Sci. 2021;17:768–80. 33767587 10.7150/ijbs.52279 PMC7975697

[33] Rosenberg N, Haele MV, Lanton T, Brashi N, Bromberg Z, Adler H, et al. Combined hepatocellular-cholangiocarcinoma derives from liver progenitor cells and depends on senescence and IL-6 trans-signaling. J Hepatol. 2022;77:1631–41. 35988690 10.1016/j.jhep.2022.07.029

[34] Si YQ, Wang XQ, Pan CC, Wang Y, Lu ZM. An efficient nomogram for discriminating intrahepatic cholangiocarcinoma from hepatocellular carcinoma: a retrospective study. Front Oncol. 2022;12:833999. 35480111 10.3389/fonc.2022.833999 PMC9035637

[35] Uthaisar K, Vaeteewoottacharn K, Seubwai W, Talabnin C, Sawanya Wisuth K, Obchoei S, et al. Establishment and characterization of a novel human cholangiocarcinoma cell line with high metastatic activity. Oncol Rep. 2016;36:1435–46. 27461717 10.3892/or.2016.4974

[36] Zach S, Birgin E, Rückert F. Primary cholangiocellular carcinoma cell lines. J Stem Cell Res Transplant. 2015;2:1013.

[37] He J, Li G, Liu X, Ma L, Zhang J, Zheng S, et al. Mesencephalic astrocyte-derived neurotrophic factor, a prognostic factor of cholangiocarcinoma, affects sorafenib sensitivity of cholangiocarcinoma cells by deteriorating ER stress. Onco Targets Ther. 2020;13:9169–84. 32982305 10.2147/OTT.S245575 PMC7502388

[38] He J, Li G, Liu X, Ma L, Zhang P, Zhang J, et al. Diagnostic and prognostic values of MANF expression in hepatocellular carcinoma. Biomed Res Int. 2020;2020:1–18. 10.1155/2020/1936385 PMC7193290 32382531

[39] Shin S, Wangensteen KJ, Teta‐Bissett M, Wang YJ, Mosleh‐Shirazi E, Buza EL, et al. Genetic lineage tracing analysis of the cell of origin of hepatotoxin-induced liver tumors in mice. Hepatology. 2016;64:1163–77. 27099001 10.1002/hep.28602 PMC5033674

[40] Seehawer M, Heinzmann F, D’Artista L, Harbig J, Roux P-F, Hoenicke L, et al. Necroptosis microenvironment directs lineage commitment in liver cancer. Nature. 2018;562:69–75. 30209397 10.1038/s41586-018-0519-y PMC8111790

[41] Sekiya S, Suzuki A. Intrahepatic cholangiocarcinoma can arise from Notch-mediated conversion of hepatocytes. J Clin Investig. 2012;122:3914–8. 23023701 10.1172/JCI63065 PMC3484442

[42] Watt AJ. HNF4: A central regulator of hepatocyte differentiation and function. Hepatology. 2003;37:1249–53. 12774000 10.1053/jhep.2003.50273

[43] Walesky C, Edwards G, Borude P, Gunewardena S, O’Neil M, Yoo B, et al. Hepatocyte nuclear factor 4 alpha deletion promotes diethylnitrosamine-induced hepatocellular carcinoma in rodents. Hepatology. 2013;57:2480–90. 23315968 10.1002/hep.26251 PMC3669646

[44] Hou X, Wu Q, Rajagopalan C, Zhang C, Bouhamdan M, Wei H, et al. CK19 stabilizes CFTR at the cell surface by limiting its endocytic pathway degradation. FASEB J. 2019;33:12602–15. 31450978 10.1096/fj.201901050R PMC9292138

[45] Chen Y, Guldiken N, Spurny M, Mohammed HHA, Haybaeck J, Pollheimer MJ, et al. Loss of keratin 19 favours the development of cholestatic liver disease through decreased ductular reaction. J Pathol. 2015;237:343–54. 26108453 10.1002/path.4580

[46] Omary MB, Ku N-O, Tao G-Z, Toivola DM, Liao J. ‘Heads and tails’ of intermediate filament phosphorylation: multiple sites and functional insights. Trends Biochem Sci. 2006;31:383–94. 16782342 10.1016/j.tibs.2006.05.008

[47] Salas PJ, Forteza R, Mashukova A. Multiple roles for keratin intermediate filaments in the regulation of epithelial barrier function and apico-basal polarity. Tissue Barriers. 2016;4:e1178368. 27583190 10.1080/21688370.2016.1178368 PMC4993576

[48] Kawai T, Yasuchika K, Ishii T, Katayama H, Yoshitoshi EY, Ogiso S, et al. Keratin 19, a cancer stem cell marker in human hepatocellular carcinoma. Clin Cancer Res. 2015;21:3081–91. 25820415 10.1158/1078-0432.CCR-14-1936

[49] Pu W, Zhu H, Zhang M, Pikiolek M, Ercan C, Li J, et al. Bipotent transitional liver progenitor cells contribute to liver regeneration. Nat Genet. 2023;55:651–64. 36914834 10.1038/s41588-023-01335-9 PMC10101857

[50] Liu LZ, Yang LX, Zheng BH, Dong PP, Liu XY, Wang ZC, et al. CK7/CK19 index: a potential prognostic factor for postoperative intrahepatic cholangiocarcinoma patients. J Surg Oncol. 2018;117:1531–9. 29513894 10.1002/jso.25027

[51] Katsuda T, Kawamata M, Hagiwara K, Takahashi R, Yamamoto Y, Camargo FD, et al. Conversion of terminally committed hepatocytes to culturable bipotent progenitor cells with regenerative capacity. Cell Stem Cell. 2017;20:41–55. 27840021 10.1016/j.stem.2016.10.007

[52] Govaere O, Petz M, Wouters J, Vandewynckel YP, Scott EJ, Topal B, et al. The PDGFR α -laminin B1-keratin 19 cascade drives tumor progression at the invasive front of human hepatocellular carcinoma. Oncogene. 2017;36:6605–16. 28783171 10.1038/onc.2017.260 PMC5702717

[53] Govaere O, Komuta M, Berkers J, Spee B, Janssen C, de Luca F, et al. Keratin 19: a key role player in the invasion of human hepatocellular carcinomas. Gut. 2014;63:674–85. 23958557 10.1136/gutjnl-2012-304351 PMC3963546

[54] Uenishi T, Kubo S, Yamamoto T, Shuto T, Ogawa M, Tanaka H, et al. Cytokeratin 19 expression in hepatocellular carcinoma predicts early postoperative recurrence. Cancer Sci. 2003;94:851–7. 14556657 10.1111/j.1349-7006.2003.tb01366.x PMC11160230

[55] Akita M, Ajiki T, Fukumoto T, Itoh T, Zen Y. Keratin 19‐expressing hepatocellular carcinoma and small‐duct type intrahepatic cholangiocarcinoma show a similar postoperative clinical course but have distinct genetic features. Histopathology. 2019;75:385–93. 31017316 10.1111/his.13884

[56] Zhou X, Liao J, Hu L, Feng L, Omary MB. Characterization of the major physiologic phosphorylation site of human keratin 19 and its role in filament organization*. J Biol Chem. 1999;274:12861–6. 10212274 10.1074/jbc.274.18.12861

[57] Saha SK, Choi HY, Kim BW, Dayem AA, Yang GM, Kim KS, et al. KRT19 directly interacts with beta-catenin/RAC1 complex to regulate NUMB-dependent NOTCH signaling pathway and breast cancer properties. Oncogene. 2017;36:332–49. 27345400 10.1038/onc.2016.221 PMC5270332

[58] Boucher JM, Harrington A, Rostama B, Lindner V, Liaw L. A receptor-specific function for Notch2 in mediating vascular smooth muscle cell growth arrest through cyclin-dependent kinase inhibitor 1B. Circ Res. 2013;113:975–85. 23965337 10.1161/CIRCRESAHA.113.301272 PMC3882755

[59] Fan B, Malato Y, Calvisi DF, Naqvi S, Razumilava N, Ribback S, et al. Cholangiocarcinomas can originate from hepatocytes in mice. J Clin Investig. 2012;122:2911–5. 22797301 10.1172/JCI63212 PMC3408746

[60] Tao X, Wang D, Liang Y, Yang L, He E, Zhou J, et al. PRDX6 inhibits hepatic stellate cells activation and fibrosis via promoting MANF secretion. Biomed Pharmacother. 2022;156:113931. 36411620 10.1016/j.biopha.2022.113931

