# Supplementary material for: Hepatic factor MANF drives hepatocytes reprogramming by detaining cytosolic CK19 in intrahepatic cholangiocarcinoma
Source: Cell Death Differ. 2025 Feb 19;32(8):1441–59. doi: 10.1038/s41418-025-01460-4 (PMC12325741; doi:10.1038/s41418-025-01460-4)
Supplement: Supplementary file 1 — Supplementary Materials [file 41418_2025_1460_MOESM1_ESM.pdf]

Supplemental Materials

Hepatic factor MANF drives hepatocytes reprogramming by detaining cytosolic CK19 in intrahepatic cholangiocarcinoma

Qiong Mei[1,2], Yu Zhang[1,2], Hong Li[1,2], Wei Ma[2], Wenkai Huang[3], Zhengsheng Wu[2], Yongli Huang[1,2], Yanyan Liang[1,2], Chuansheng Wei[1,2], Jinfeng Wang[1,2], Yuefeng Ruan[1,2], Lin Yang[1,2], Yan Huang[4], Yujun Shen[1,2], Jun Liu[1,2], Lijie Feng[1,2], Yuxian Shen[1,2,5,*]

Supplementary Table 1. Correlation between MANF staining intensity and clinical variables in human ICC.

Variables	n	MANF expression, n (%)		P
		Low, n (%)	High, n (%)	
**Total**	78	46 (59.0)	32 (41.0)	
**Age (years)**				
60	31	17 (54.8)	14 (45.2)	0.546
> 60	47	29 (61.7)	18 (38.3)	
**Gender**				
Male	45	26 (57.8)	19 (42.2)	0.802
Female	33	20 (60.6)	13 (39.4)	
**Tumor diameter (cm)**				
4	41	29 (70.7)	12 (29.3)	**0.026**
＞4	37	17 (45.9)	20 (55.1)	
**TNM Stage**				
I/II	41	33 (80.5)	8 (19.5)	**0.000**
III/IV	37	13 (35.1)	24 (64.9)	
**Tumor type**				
tubular 1/2	52	34 (65.4)	18 (34.6)	0.104
poorly differentiated /other	26	12 (46.2)	14 (53.8)	
**Vascular infiltration**				
No	42	26 (61.9)	16 (38.1)	0.57
Yes	36	20 (55.6)	16 (44.4)	
**Lymph node infiltration**				
No	31	19 (61.3)	12 (38.7)	0.736
Yes	47	27 (57.4)	20 (42.6)	
**Distant metastasis**				
No	51	35 (68.6)	16 (31.4)	**0.017**
Yes	27	11 (40.7)	16 (59.3)	
**Perineural invasion**				

No	33	17 (51.5)	16 (48.5)	0.251
Yes	45	29 (64.4)	16 (35.6)	
**CEA**				
0-5	50	31 (62.0)	19 (38.0)	0.468
＞5	28	15 (53.6)	13 (46.4)	
**CA19-9**				
0-37	19	12 (63.2)	7 (36.8)	0.67
＞37	59	34 (57.6)	25 (42.4)	
**CA125**				
0-35	53	32 (60.4)	21 (39.6)	0.714
＞35	25	14 (56.0)	11 (44.0)	

Supplementary Table 2. The sequences of Notch2-siRNA and CK19-siRNA used to knockdown the Notch2 and CK19 genes.

Gene	Human/Mouse	Primer	Sequence (5' to 3')
**Notch2**	Mouse	Forward-1	GCUGUGAGAUGGACAUCAATT
		Reverse-1	UUGAUGUCCAUCUCACAGCTT
		Forward-2	CACACCAACUUGCGCAUUAAATT
		Reverse-2	UUUAAUGCGCAAGUUGGUGUGTT
		Forward-3	CACUAGUCUUCAUGUCUUATT
		Reverse-3	UAAGACAUGAAGACUAGUGTT
**CK19**	Human	Forward-1	GGAAGAUCACUACAACAAU
		Reverse-1	AUUGUUGUAGUGAUCUUCC
		Forward-2	GGAUUGUCCUGCAGAUCGA
		Reverse-2	UCGAUCUGCAGGACAAUCC
		Forward-3	GGACCGACCUGGAGAUGCA
		Reverse-3	UGCAUCUCCAGGUCGGUCC

Supplementary Table 3. Antibodies involved in this study.

Name	Company	Catalog number	Purpose
**MANF**	Abcam	ab67271	IHC, IF, mIHC, WB
**CK19**	Bioss	bsm-33057M	IHC, IF
**CK19**	Abcam	ab52625	IHC, IF, mIHC, WB
**Ki67**	Hua Bio	HA721115	IHC
**HNF4α**	Abcam	ab199431	IHC, IF, WB
**HA**	Sigma	H6908	IHC, IF, WB
**Myc**	Sigma	SAB2108476	IHC, IF, WB
**His**	Enogen	E12-004	IHC
**GAPDH**	Affinity	T0004	WB
**Flag**	Abcam	ab205606	WB
**β-catenin**	CST	8480S	WB
**Vimentin**	CST	5741S	WB
**Occludin**	Affinity	DF7504	WB
**Claudin**	Affinity	AF0127	WB
**E-cadherin**	BD	610182	WB
**α-SMA**	Abcam	ab124964	IF
**F4/80**	CST	30325s	IF
**CD133**	CST	64326s	IF
**β-tubulin**	Boster	M01857-3	WB
**ATP1A1**	Proteintech	14418-1-AP	WB
**HistonH3**	Affinity	BF9211	WB
**CK7**	Affinity	DF7027	WB
**GST**	Abcam	ab111947	WB
**Notch2**	CST	5732s	IHC, IF, mIHC, WB
**Jagged1**	Abcam	ab7771	IHC, WB
**Hes1**	Hua Bio	ET1610-97	IHC, WB

Supplementary Table 4. Primer sequences involved in this study.

Gene	Human/Mouse	Primer	Sequence (5’ to 3’)
**GAPDH**	Human	Forward	CTGGGCTACACTGAGCACC
		Reverse	AAGTGGTCGTTGAGGGCAATG
**MANF**	Human	Forward	TCACATTCTCACCAGCCACT
		Reverse	CAGGTCGATCTGCTTGTCATAC
**GAPDH**	Mouse	Forward	AGGTCGGTGTGAACGGATTTG
		Reverse	TGTAGACCATGTAGTTGAGGTCA
**MANF**	Mouse	Forward	TCTGGGACGATTTTACCAGGA
		Reverse	TCTTGCTTCACGGCAAAACTTTA
**CK19**	Mouse	Forward	GGGGGTTCAGTACGCATTGG
		Reverse	GAGGACGAGGTCACGAAGC
**Ki67**	Mouse	Forward	AGCACAAAGAGACGGTCTAAGA
		Reverse	CTCTGCCTCGTGACTGTGTT
**Notch2**	Mouse	Forward	ATGTGGACGAGTGTCTGTTGC
		Reverse	GGAAGCATAGGCACAGTCATC
**Jag1**	Mouse	Forward	CCTCGGGTCAGTTTGAGCTG
		Reverse	CCTTGAGGCACACTTTGAAGTA
**Hes1**	Mouse	Forward	CCAGCCAGTGTCAACACGA
		Reverse	AATGCCGGGAGCTATCTTTCT
**HNF4α**	Mouse	Forward	GTGGCGAGTCCTTATGACACG
		Reverse	GCTGTTGGATGAATTGAGGTTGG

Supplemental Fig. 1. MANF is specifically highly expressed in ICC. (A) The human liver cancer samples were stained by HE and immunohistochemistry assay with the antibodies of anti-MANF, anti-HNF4α, and anti-CK19. **(B-C)** Volcano plot (B) and Violin plot (C) of MANF expression in HCC and ICC (GSE179443). **(D)** The human extrahepatic cholangiocarcinoma tissues were stained by immunohistochemistry assay with MANF antibody.

Supplemental Fig. 2. High expression of MANF is associated with poor survival of ICC patients. (A-B) The mRNA level of MANF was upregulated in human ICC tissues based on TCGA and GSE107943 datasets. **(C-D)** The overall survival curves of ICC patients in TCGA and GSE107943 datasets presented by Kaplan-Meier plotter ($P < 0.05$). **(E-F)** ROC curve of the risk model (AUC>0.65). **(G-H)** Volcano plot (G) and Heatmap (H) showing the differential expression of MANF in normal and ICC cells (GSE241923). **(I-K)** Volcano plot (I), Violin plot (J), and Heatmap (K) showing the differential expression of MANF in ICC-UDC and ICC-DC (GSE221589).

Supplemental Fig. 3. MANF is increased in the liver tissues of ICC mice. (A) The time-effect curve of body weight in SBT-induced ICC mice. n=6, **$P < 0.01$. **(B)** Liver-body weight ratio. n=6, ***$P < 0.01$. **(C)** Serum ALT, AST, TBIL, and DBIL levels. n=5-6, **$P < 0.01$, ***$P < 0.001$. **(D)** The tumor number of SBT-induced mice ICC. n=6, ****$P < 0.0001$. **(E)** The tumor area of SBT-induced mice ICC. n=6, ***$P < 0.001$. **(F)** The expression of HA and Myc were detected by immunohistochemistry staining.

(G) Expression of MANF in Ca and Pa tissues from SBT-induced ICC mice. n=3, *P < 0.05. **(H)** The quantitative data in Fig. 1H. n=3, **P < 0.01, ***P < 0.001. **(I)** The levels of MANF, CK19, and Ki67 in tdTomato MANF knockin mice treated with SBT were detected by qPCR. n=4, **P < 0.01, ***P < 0.001. **(J)** The quantitative data in Fig. 1I. n=3, *P < 0.05, **P < 0.01. **(K)** The time-effect curve of body weight in TAA-induced ICC mice. n=6, **P < 0.01, ***P < 0.001, ****P < 0.0001. **(L)** Liver-body weight ratio. n=6, ***P < 0.001. **(M)** Serum ALT, AST, TBIL, DBIL levels. n=6, **P < 0.01, ****P < 0.0001. **(N)** The tumor number of TAA-induced mice ICC. n=6, ****P < 0.0001. **(O)** The tumor area of TAA-induced mice ICC. n=6, ****P < 0.0001. **(P)** Expression of MANF in Ca and Pa tissues from TAA-induced mice ICC. n=3, *P < 0.05. **(Q)** The quantitative data in Fig. 1M. n=3, *P < 0.05, **P < 0.01. **(R)** The expressions of MANF, CK19, and Ki67 in TAA-induced mice ICC were detected by qPCR. n=6, **P < 0.01, ***P < 0.001, ****P < 0.0001. **(S)** The quantitative data in Fig. 1N. n=3, *P < 0.05, **P < 0.01.

Supplemental Fig. 4. Generation and identification of MANF overexpression and knockdown ICC cell lines. (A) MANF expression in Hucct1, HCCC9810, and RBE was detected by western blot assay. n=3, *P < 0.05, ***P < 0.001, ****P < 0.0001. **(B-C)** The stable transfection efficacy of MANF overexpression in ICC cell lines was verified by mCheery (red) fluorescence (B) and western blot assays (C, n=3, **P < 0.01). **(D-E)** The stable transfection efficacy of MANF knockdown (shMANF) in ICC cell lines was verified by EGFP (green) fluorescence (D) and western blot assays (E,

n=3, ***$P < 0.001$).

Supplemental Fig. 5. Mature hepatocytes-specific MANF transgenic mice are successfully constructed. (A-B) The efficacy of mature hepatocytes-specific MANF knockin/knockout in primary hepatocytes was confirmed by western blot assays. n=9-12, ***$P < 0.001$, ****$P < 0.0001$. **(C)** Identification of the specificity of MANF knockin/knockout in mature hepatocytes after AAV8-TBG-Cre injection. MANF was marked in red. The hepatocytes were labeled with anti-HNF4α (green), the BECs were labeled with anti-CK19 (green), the macrophages were labeled with CD68 (green), and the myofibroblasts were labeled with α-SMA (green). The nuclei were stained with DAPI (blue).

Supplemental Fig. 6. MANF accelerates mice ICC. (A) The tumor number in MANF knockin mice treated with SBT. n=6, ****$P < 0.0001$. **(B)** The tumor area in MANF knockin mice treated with SBT. n=6, **$P < 0.01$. **(C)** The levels of MANF in MANF knockin were detected by immunohistochemistry staining. **(D)** The quantitative data in Fig. 3F. The levels of MANF, CK19, and Ki67 in MANF knockin mice treated with SBT were detected by immunohistochemistry staining. n=3, *$P < 0.05$, **$P < 0.01$, ***$P < 0.001$. **(E)** The quantitative data in Fig. 3H. The protein levels of CK19 and MANF in MANF knockin mice treated with SBT were detected by western blot. n=6, **$P < 0.01$, ***$P < 0.001$, ****$P < 0.0001$. **(F)** The tumor number in MANF knockout mice treated with SBT. n=6, **$P < 0.01$. **(G)** The tumor area in MANF knockout mice

treated with SBT. n=6, ***$P < 0.001$. **(H)** The levels of MANF in MANF knockout were detected by immunohistochemistry staining. **(I)** The quantitative data in Fig. 3N. The levels of MANF, CK19, and Ki67 in MANF knockout mice treated with SBT were detected by immunohistochemistry staining. n=3, *$P < 0.05$, **$P < 0.01$, ***$P < 0.001$. **(J)** The quantitative data in Fig. 3P. The protein levels of CK19 and MANF in MANF knockout mice treated with SBT were detected by western blot. n=6, *$P < 0.05$, ***$P < 0.001$, ****$P < 0.0001$. **(K)** Construction of hepatic MANF knockout mice under the control of Alb-Cre. **(L-N)** The efficacy of hepatic-specific MANF knockout in liver tissues (L-M) and primary hepatocytes (N) were confirmed by immunohistochemistry staining (L, n=3, **$P < 0.01$) and western blot assays (M-N, n=3, ****$P < 0.0001$). **(O)** The tumor number in TAA-induced mice ICC, with or without rhMANF injection. n=6, **$P < 0.01$, ***$P < 0.001$, ****$P < 0.0001$. **(P)** The tumor area in TAA-induced mice ICC, with or without rhMANF injection. n=6, *$P < 0.05$, **$P < 0.01$.

Supplemental Fig. 7. Strategies for generation and identification of MANF knockin and knockout fluorescence reporter mice. (A) Scheme for construction of mature hepatocyte-specific MANF knockin fluorescence reporter mice. **(B)** Identification of MANF knockin fluorescence reporter mice. **(C)** Scheme for construction of mature hepatocyte-specific MANF knockout fluorescence reporter mice. **(D)** Identification of MANF knockout fluorescence reporter mice.

Supplemental Fig. 8. Isolation and identification of hepatocytes. (A) Purity of

primary hepatocytes isolated from tdTomato (red) mice. **(B)** Verification of the efficiency of SBT transfection in the primary hepatocytes isolated from tdTomato (red) mice. HA-AKT1 and Myc-NICD1 were detected by the antibodies against HA (green) and Myc (green).

Supplemental Fig. 9. Screening the interacting proteins of CK19 in ICC. (A) Notch signaling pathway is involved in the regulation of CK19 in KEGG database. **(B)** Notch2 is positively correlated with CK19. **(C)** Violin plots showing marker genes expression for 10 distinct cell types (GSE138709).

Supplemental Fig. 10. Effects of MANF knockin/knockout on the levels of CK19, NICD2, and the activation of Notch2 signaling pathway. (A) Co-localization of CK19 (red) and Notch2 (green) detected by immunofluorescent staining with anti-CK19 and anti-Notch2 antibodies in primary hepatocytes isolated from ICC mice challenged with SBT for 2 weeks. DAPI (blue) was used to stain nuclei. **(B)** Construction of the truncates of Notch2, including NICD2, RAM, AR, TAD, and PEST. **(C)** Identifying the binding site of CK19 among NICD2 domain. **(D)** The quantitative data in Fig. 8F. Effects of MANF knockin/knockout on the levels of NICD2, CK19, and MANF in cytosol and nucleus. n=6, $^{**}P < 0.01$, $^{***}P < 0.001$, $^{****}P < 0.0001$. **(E)** Quantitative data in Fig. 8H. The protein levels of NICD2, Hes1, Jag1, CK7, CK19, and MANF were detected in the primary hepatocytes isolated from tdTomato mice, or plus MANF knockin (KI) and knockout (KO), treated with SBT for 2 weeks. n=3, *P

< 0.05, **$P < 0.01$, ***$P < 0.001$. **(F)** The mRNA levels of Notch2, Jag1, and Hes1 in SBT-induced ICC mice detected by qPCR assay. n=4, ***$P < 0.001$. **(G)** The co-localization of MANF (red) and Notch2 (green) in the liver tissues and primary hepatocytes isolated from ICC mice challenged with SBT for 2 weeks. The nuclei were stained with DAPI (blue).

Supplemental Fig. 11. Effects of MANF on the expression of Notch1 in ICC cells. **(A-B)** Effects of MANF knockin/knockout on the levels of Notch1 in Hucct1 (A) and RBE cells (B). n=3, *$P < 0.05$, **$P < 0.01$, ***$P < 0.001$. **(C-D)** Co-IP assays showed that there was no interaction between MANF or CK19 and Notch1 in Hucct1 cells. The isotype IgG was used as a negative control.

Supplemental Fig. 12. Selection of RNA sequence for efficient knockdown of Notch2 and CK19. **(A)** Selection of RNA sequences for efficient knockdown of Notch2 in AML12 cells. n=8, *$P < 0.05$, ***$P < 0.001$. **(B)** Selection of RNA sequences for efficient knockdown of CK19 in Hucct1 cells. n=8, ****$P < 0.0001$.

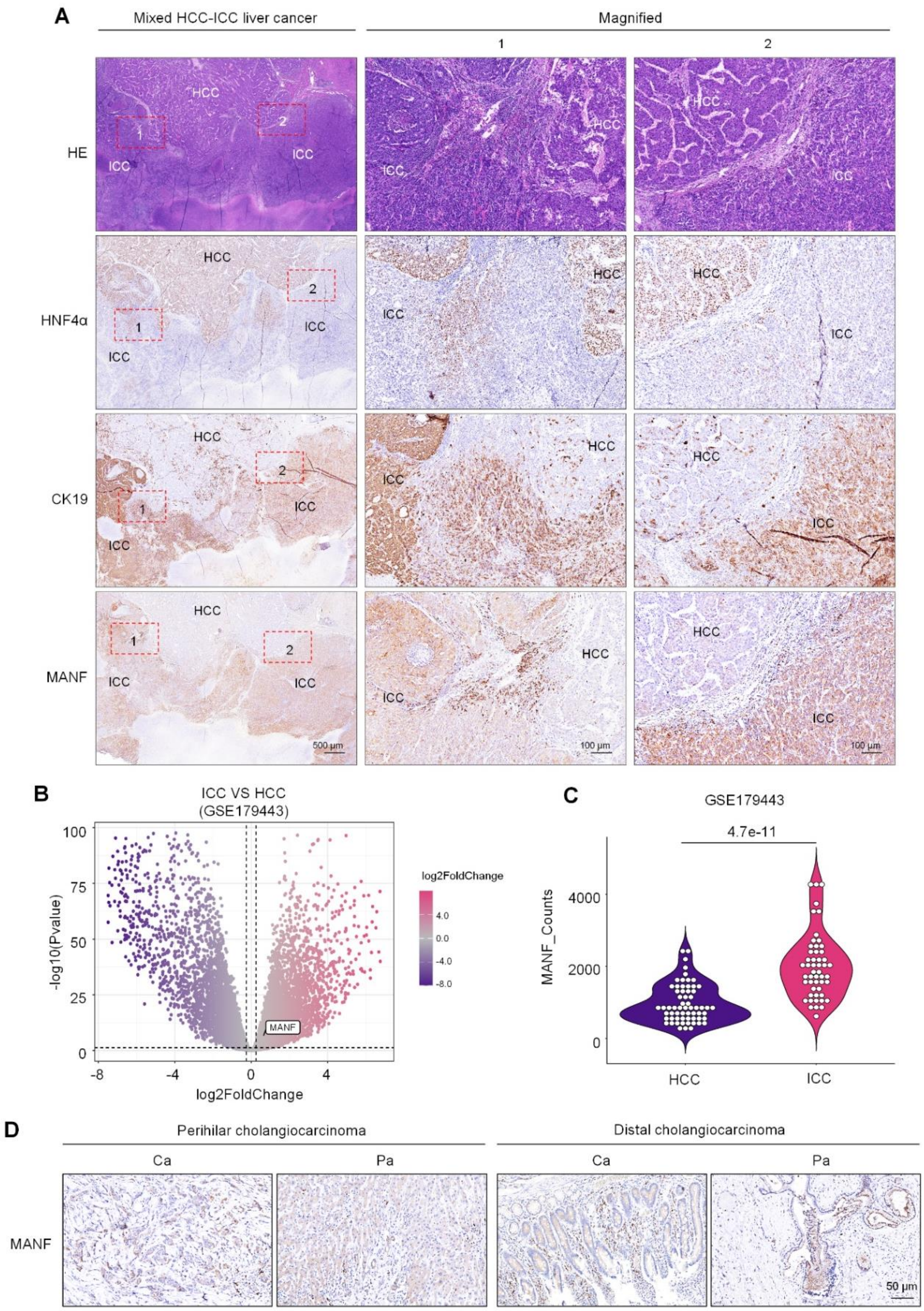

A
Mixed HCC-ICC liver cancer
Magnified
1
2
HE
HNF4α
CK19
MANF
HCC
ICC
500 μm
100 μm
B
ICC VS HCC
(GSE179443)
-log10(Pvalue)
log2FoldChange
MANF
C
GSE179443
4.7e-11
MANF_Counts
HCC
ICC
D
Perihilar cholangiocarcinoma
Distal cholangiocarcinoma
Ca
Pa
MANF
50 μm

230

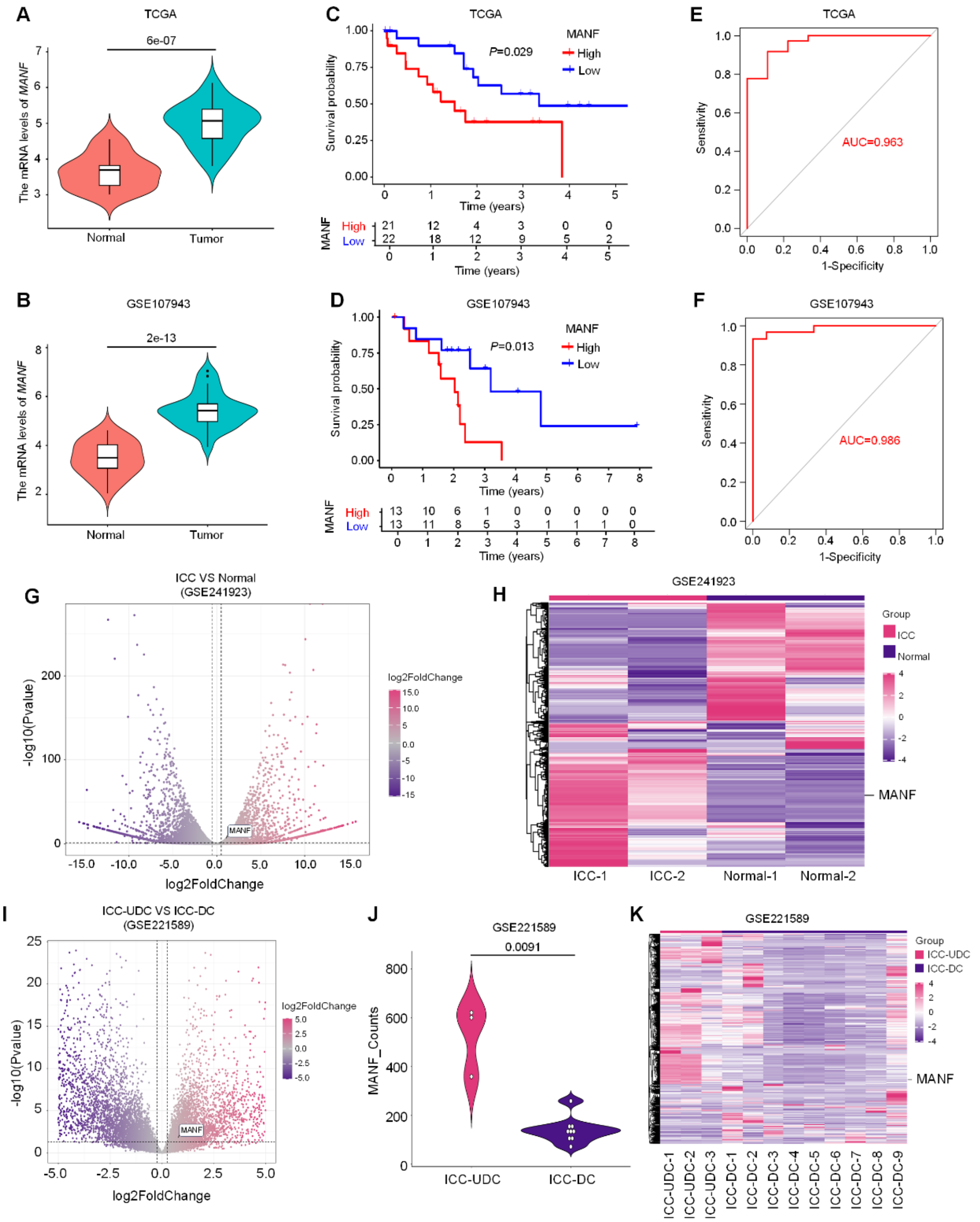
A
TCGA
6e-07
The mRNA levels of MANF
Normal
Tumor
B
GSE107943
2e-13
The mRNA levels of MANF
Normal
Tumor
C
TCGA
Survival probability
P=0.029
MANF
High
Low
Time (years)
D
GSE107943
Survival probability
P=0.013
MANF
High
Low
Time (years)
E
TCGA
Sensitivity
AUC=0.963
1-Specificity
F
GSE107943
Sensitivity
AUC=0.986
1-Specificity
G
ICC VS Normal
(GSE241923)
-log10(Pvalue)
log2FoldChange
MANF
H
GSE241923
Group
ICC
Normal
MANF
ICC-1
ICC-2
Normal-1
Normal-2
I
ICC-UDC VS ICC-DC
(GSE221589)
-log10(Pvalue)
log2FoldChange
MANF
J
GSE221589
0.0091
MANF_Counts
ICC-UDC
ICC-DC
K
GSE221589
Group
ICC-UDC
ICC-DC
MANF

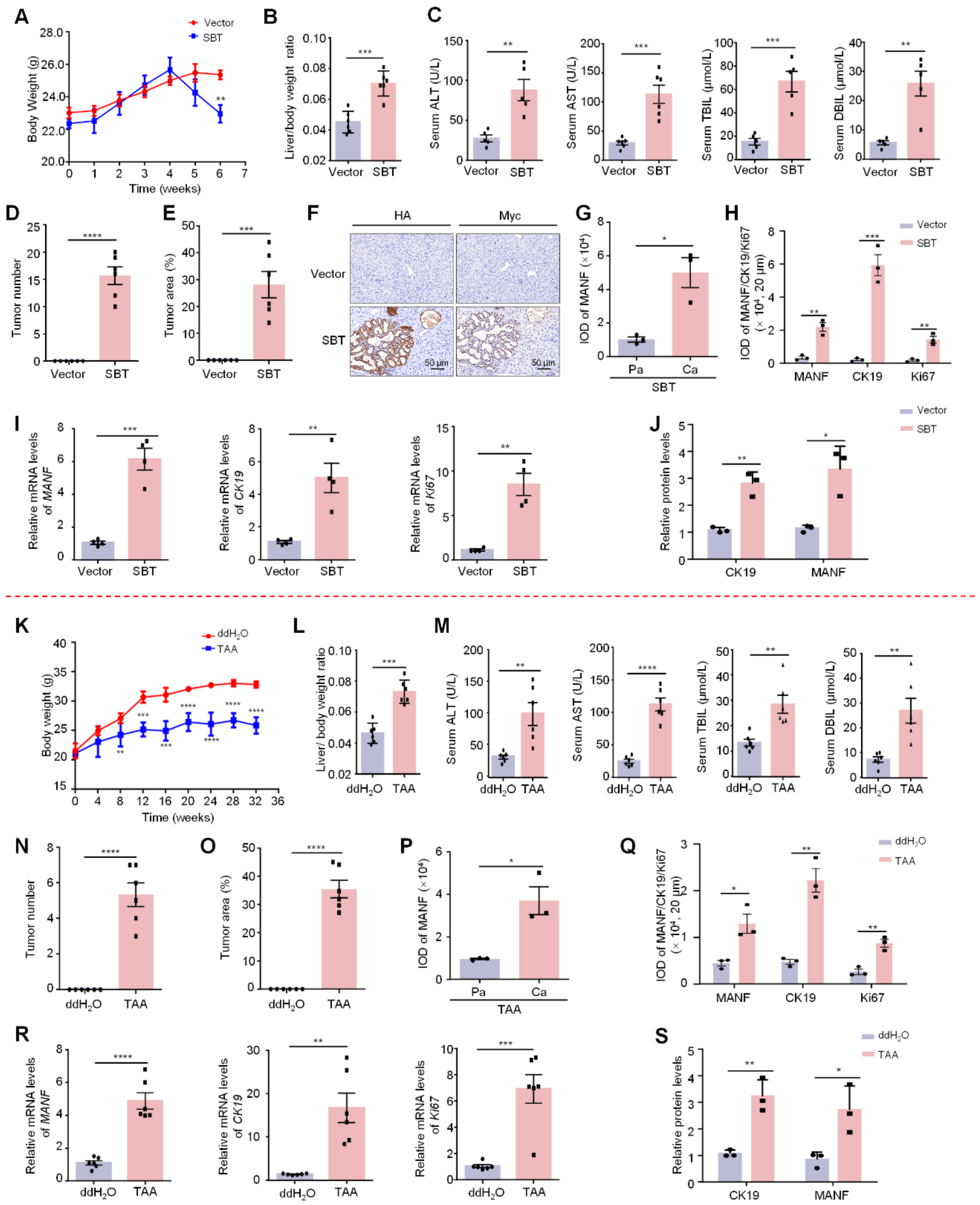

A
Vector
SBT
Body Weight (g)
Time (weeks)
B
Liver/body weight ratio
Vector SBT
C
Serum ALT (U/L)
Serum AST (U/L)
Serum TBIL (μmol/L)
Serum DBIL (μmol/L)
D
Tumor number
E
Tumor area (%)
F
HA
Myc
Vector
SBT
50 μm
G
IOD of MANF (×10⁴)
Pa
Ca
SBT
H
IOD of MANF/CK19/Ki67 (× 10⁴, 20 μm)
MANF
CK19
Ki67
I
Relative mRNA levels of MANF
Relative mRNA levels of CK19
Relative mRNA levels of Ki67
J
Relative protein levels
CK19
MANF
K
ddH2O
TAA
Body weight (g)
Time (weeks)
L
Liver/ body weight ratio
M
Serum ALT (U/L)
Serum AST (U/L)
Serum TBIL (μmol/L)
Serum DBIL (μmol/L)
N
Tumor number
O
Tumor area (%)
P
IOD of MANF (×10⁴)
Pa
Ca
TAA
Q
IOD of MANF/CK19/Ki67 (× 10⁴, 20 μm)
MANF
CK19
Ki67
R
Relative mRNA levels of MANF
Relative mRNA levels of CK19
Relative mRNA levels of Ki67
S
Relative protein levels
CK19
MANF

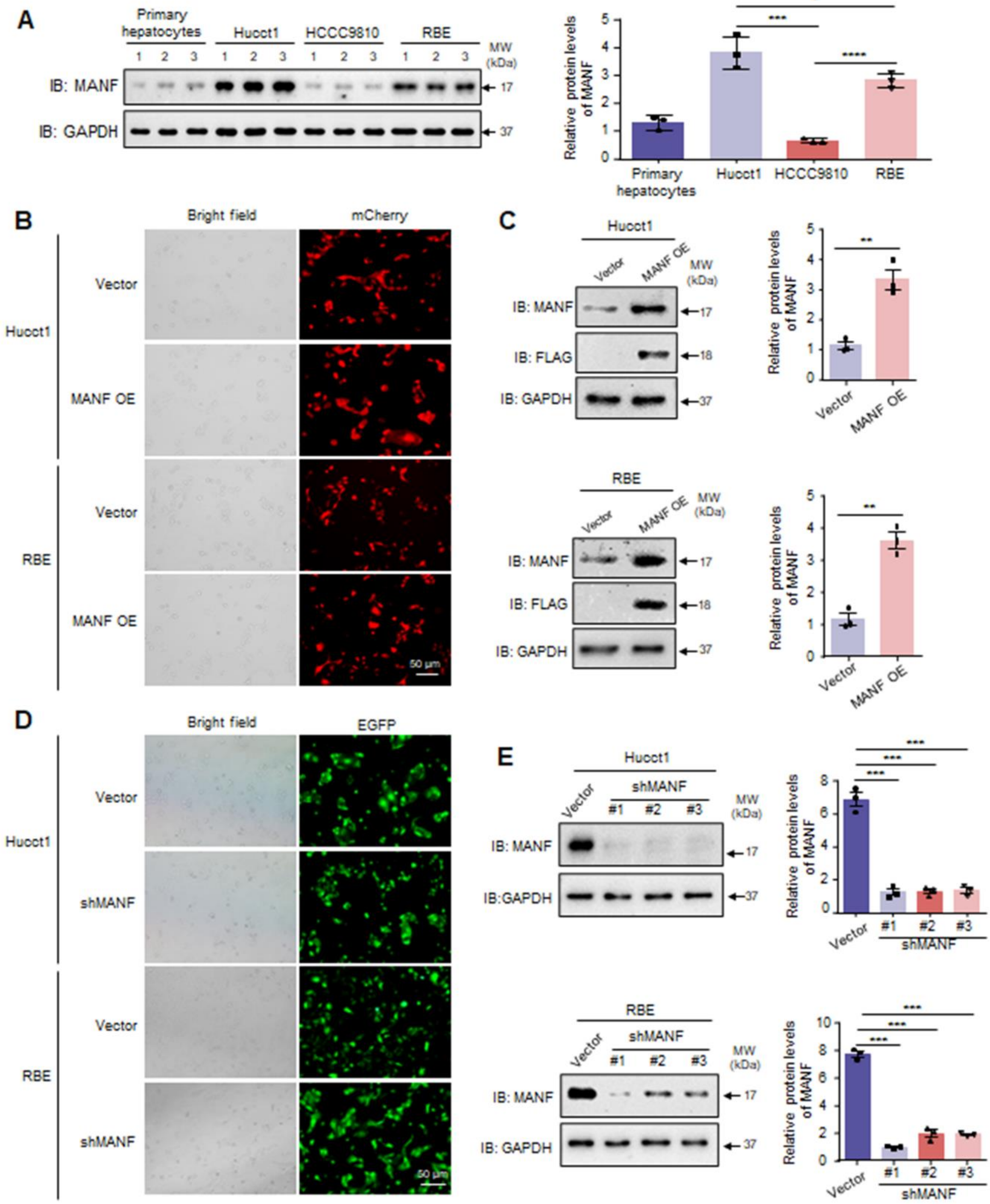
A
Primary hepatocytes
Hucct1
HCCC9810
RBE
MW (kDa)
IB: MANF
IB: GAPDH
17
37
Relative protein levels of MANF
B
Bright field
mCherry
Hucct1
RBE
Vector
MANF OE
50 μm
C
IB: FLAG
18
D
EGFP
shMANF
E
#1
#2
#3

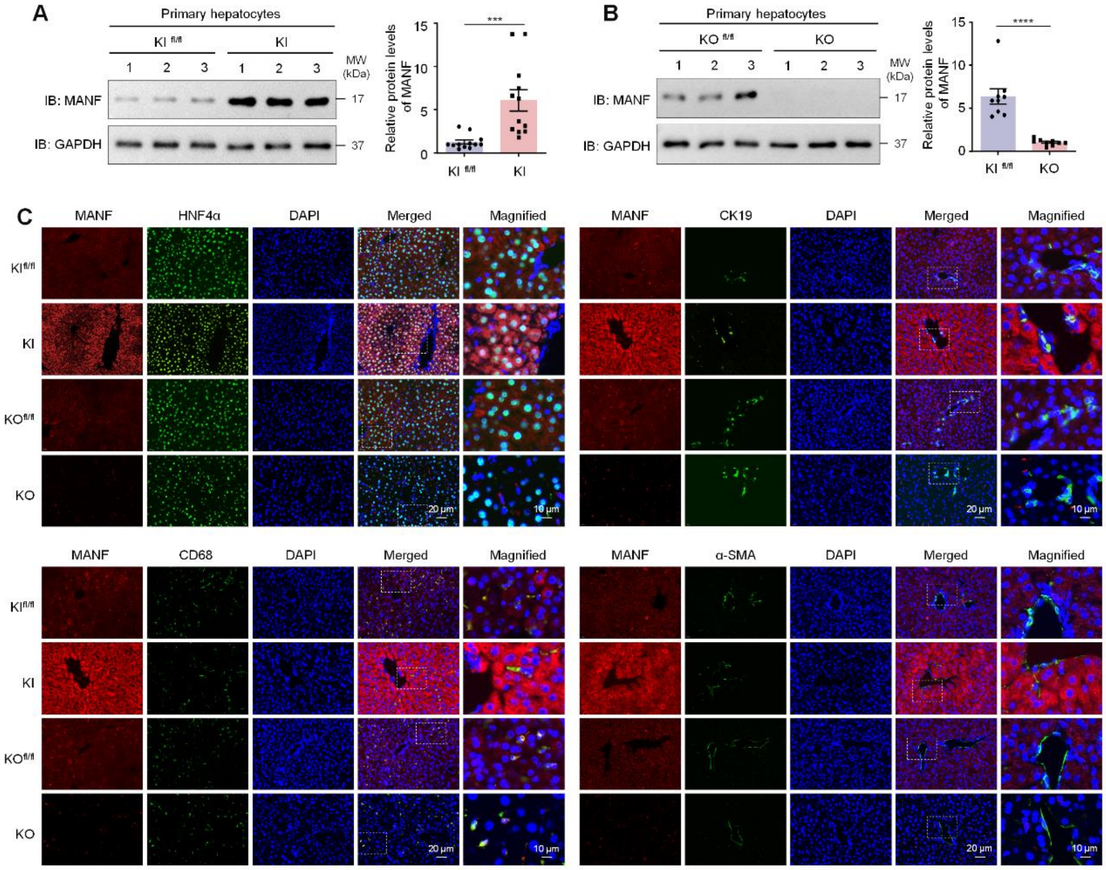
A
Primary hepatocytes
KI fl/fl
KI
1 2 3 1 2 3
MW (kDa)
IB: MANF
17
IB: GAPDH
37
Relative protein levels of MANF
***
B
Primary hepatocytes
KO fl/fl
KO
1 2 3 1 2 3
MW (kDa)
IB: MANF
17
IB: GAPDH
37
Relative protein levels of MANF
****
C
MANF
HNF4α
DAPI
Merged
Magnified
CK19
CD68
α-SMA
KI fl/fl
KI
KO fl/fl
KO
20 μm
10 μm

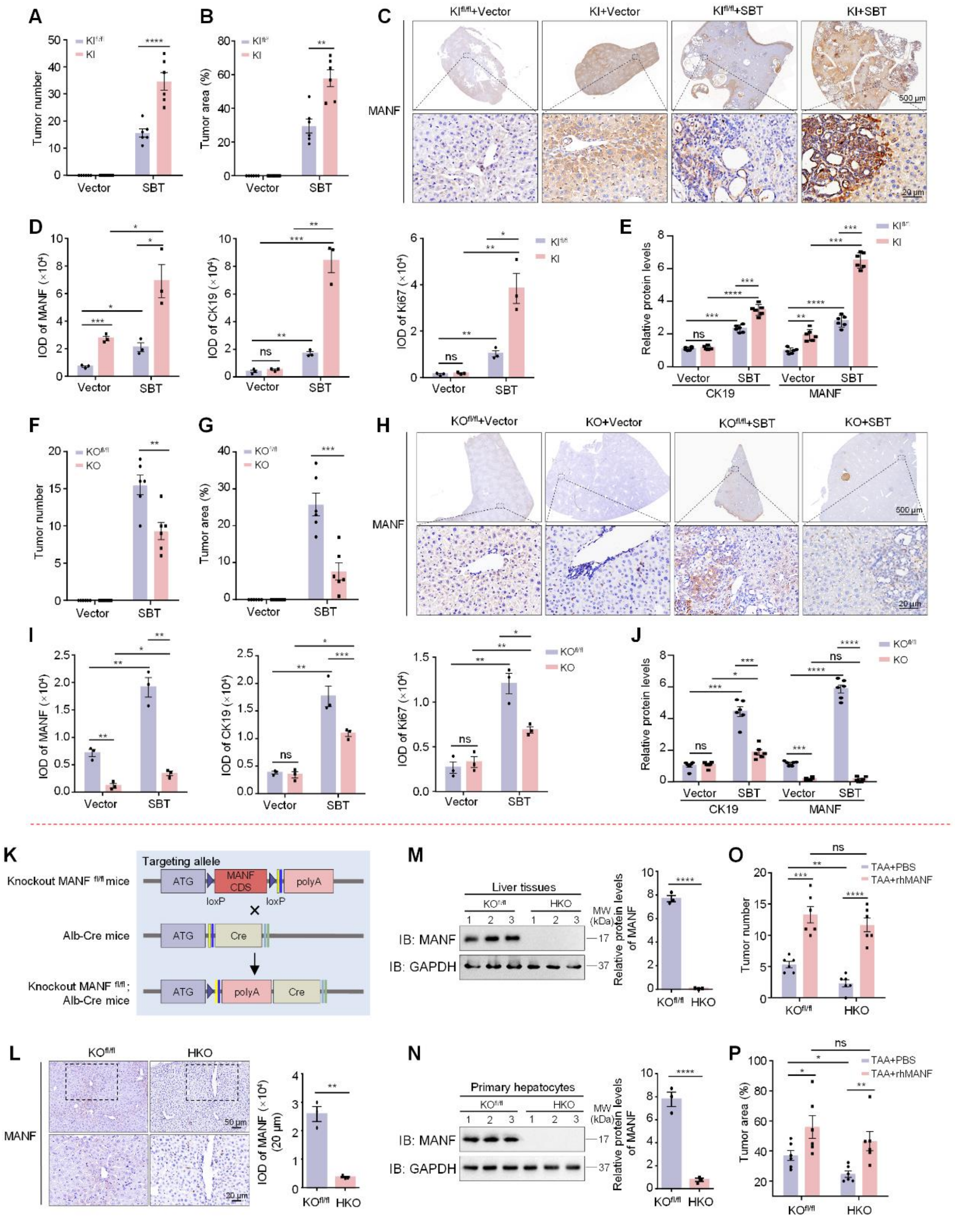
A
Tumor number
KI^fl/fl
KI
Vector
SBT
B
Tumor area (%)
C
KI^fl/fl+Vector
KI+Vector
KI^fl/fl+SBT
KI+SBT
MANF
500 μm
20 μm
D
IOD of MANF (×10⁴)
IOD of CK19 (×10⁴)
IOD of Ki67 (×10⁴)
E
Relative protein levels
CK19
MANF
F
KO^fl/fl
KO
G
H
KO^fl/fl+Vector
KO+Vector
KO^fl/fl+SBT
KO+SBT
I
J
K
Targeting allele
Knockout MANF fl/fl mice
ATG
MANF CDS
polyA
loxP
Alb-Cre mice
Cre
Knockout MANF fl/fl; Alb-Cre mice
L
KO^fl/fl
HKO
50 μm
20 μm
IOD of MANF (×10⁴) (20 μm)
M
Liver tissues
MW (kDa)
IB: MANF
IB: GAPDH
Relative protein levels of MANF
N
Primary hepatocytes
O
TAA+PBS
TAA+rhMANF
P

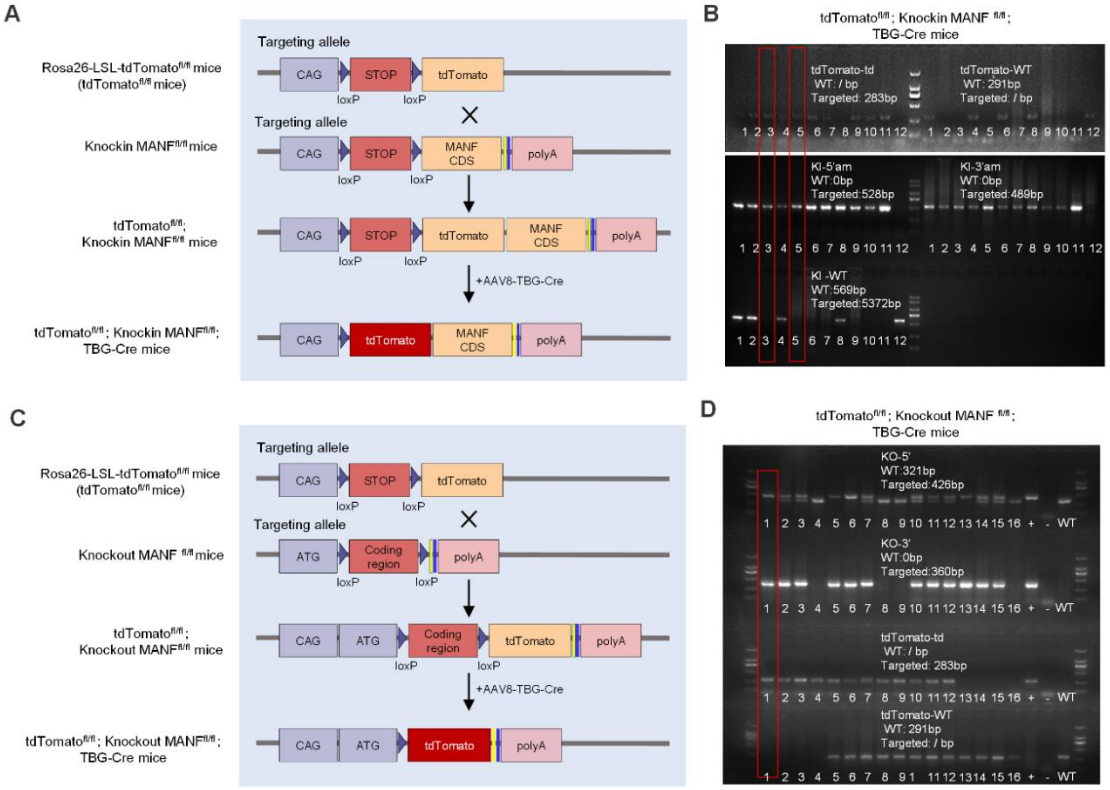
A
Targeting allele
Rosa26-LSL-tdTomato fl/fl mice (tdTomato fl/fl mice)
CAG
STOP
tdTomato
loxP
Knockin MANF fl/fl mice
MANF CDS
polyA
tdTomato fl/fl; Knockin MANF fl/fl mice
+AAV8-TBG-Cre
tdTomato fl/fl; Knockin MANF fl/fl; TBG-Cre mice
B
tdTomato fl/fl; Knockin MANF fl/fl; TBG-Cre mice
tdTomato-td WT: / bp Targeted: 283bp
tdTomato-WT WT: 291bp Targeted: / bp
KI-5'am WT:0bp Targeted:528bp
KI-3'am WT:0bp Targeted:489bp
KI -WT WT:569bp Targeted:5372bp
C
Knockout MANF fl/fl mice
ATG
Coding region
tdTomato fl/fl; Knockout MANF fl/fl mice
tdTomato fl/fl; Knockout MANF fl/fl; TBG-Cre mice
D
tdTomato fl/fl; Knockout MANF fl/fl; TBG-Cre mice
KO-5' WT:321bp Targeted:426bp
KO-3' WT:0bp Targeted:360bp
tdTomato-td WT: / bp Targeted: 283bp
tdTomato-WT WT: 291bp Targeted: / bp
WT

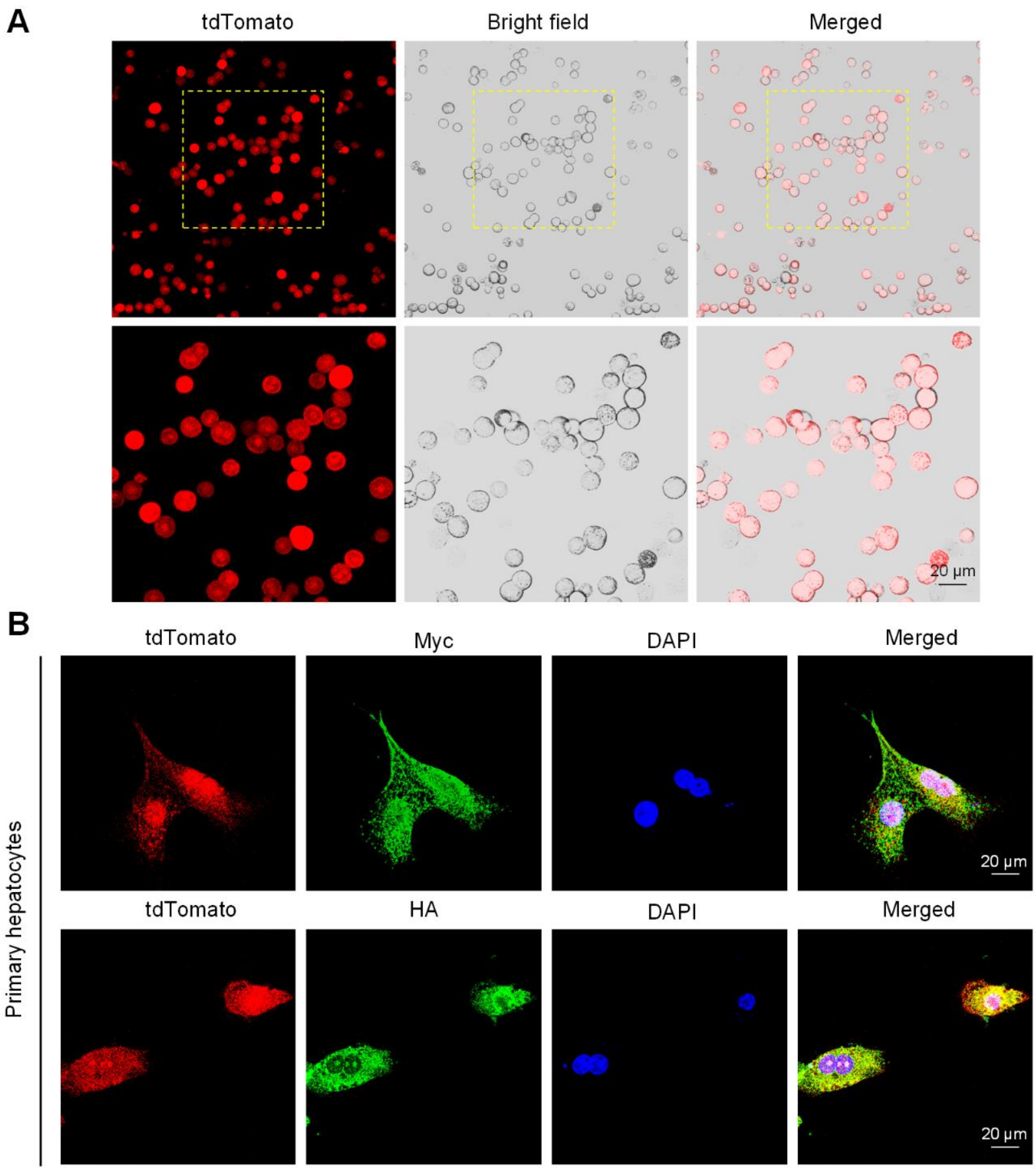
A
tdTomato
Bright field
Merged
20 μm
B
tdTomato
Myc
DAPI
Merged
Primary hepatocytes
20 μm
tdTomato
HA
DAPI
Merged
20 μm

249

250

251

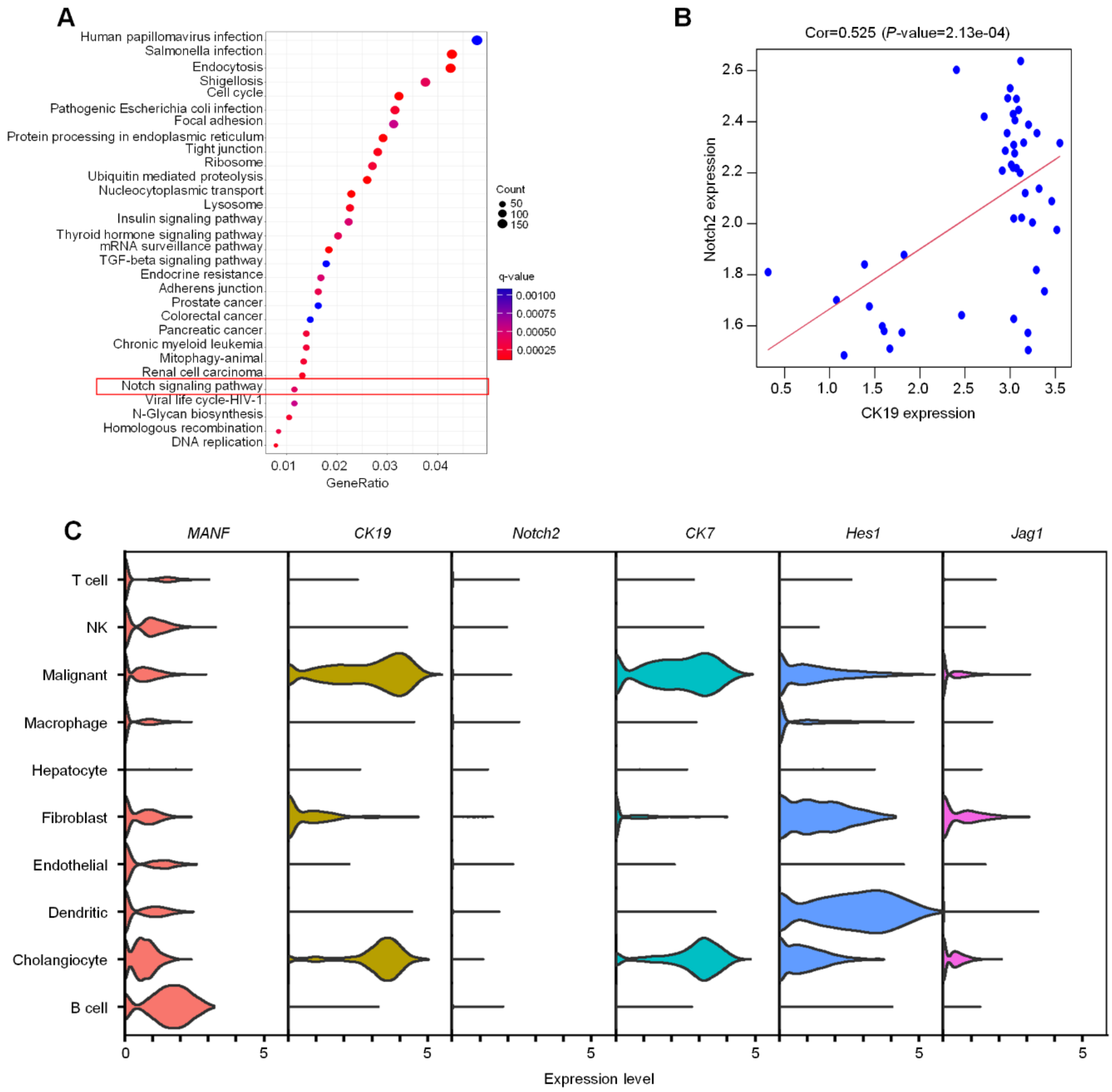
A
Human papillomavirus infection
Salmonella infection
Endocytosis
Shigellosis
Cell cycle
Pathogenic Escherichia coli infection
Focal adhesion
Protein processing in endoplasmic reticulum
Tight junction
Ribosome
Ubiquitin mediated proteolysis
Nucleocytoplasmic transport
Lysosome
Insulin signaling pathway
Thyroid hormone signaling pathway
mRNA surveillance pathway
TGF-beta signaling pathway
Endocrine resistance
Adherens junction
Prostate cancer
Colorectal cancer
Pancreatic cancer
Chronic myeloid leukemia
Mitophagy-animal
Renal cell carcinoma
Notch signaling pathway
Viral life cycle-HIV-1
N-Glycan biosynthesis
Homologous recombination
DNA replication
0.01
0.02
0.03
0.04
GeneRatio
Count
50
100
150
q-value
0.00100
0.00075
0.00050
0.00025
B
Cor=0.525 (P-value=2.13e-04)
Notch2 expression
2.6
2.4
2.2
2.0
1.8
1.6
0.5
1.0
1.5
2.0
2.5
3.0
3.5
CK19 expression
C
MANF
CK19
Notch2
CK7
Hes1
Jag1
T cell
NK
Malignant
Macrophage
Hepatocyte
Fibroblast
Endothelial
Dendritic
Cholangiocyte
B cell
0
5
Expression level

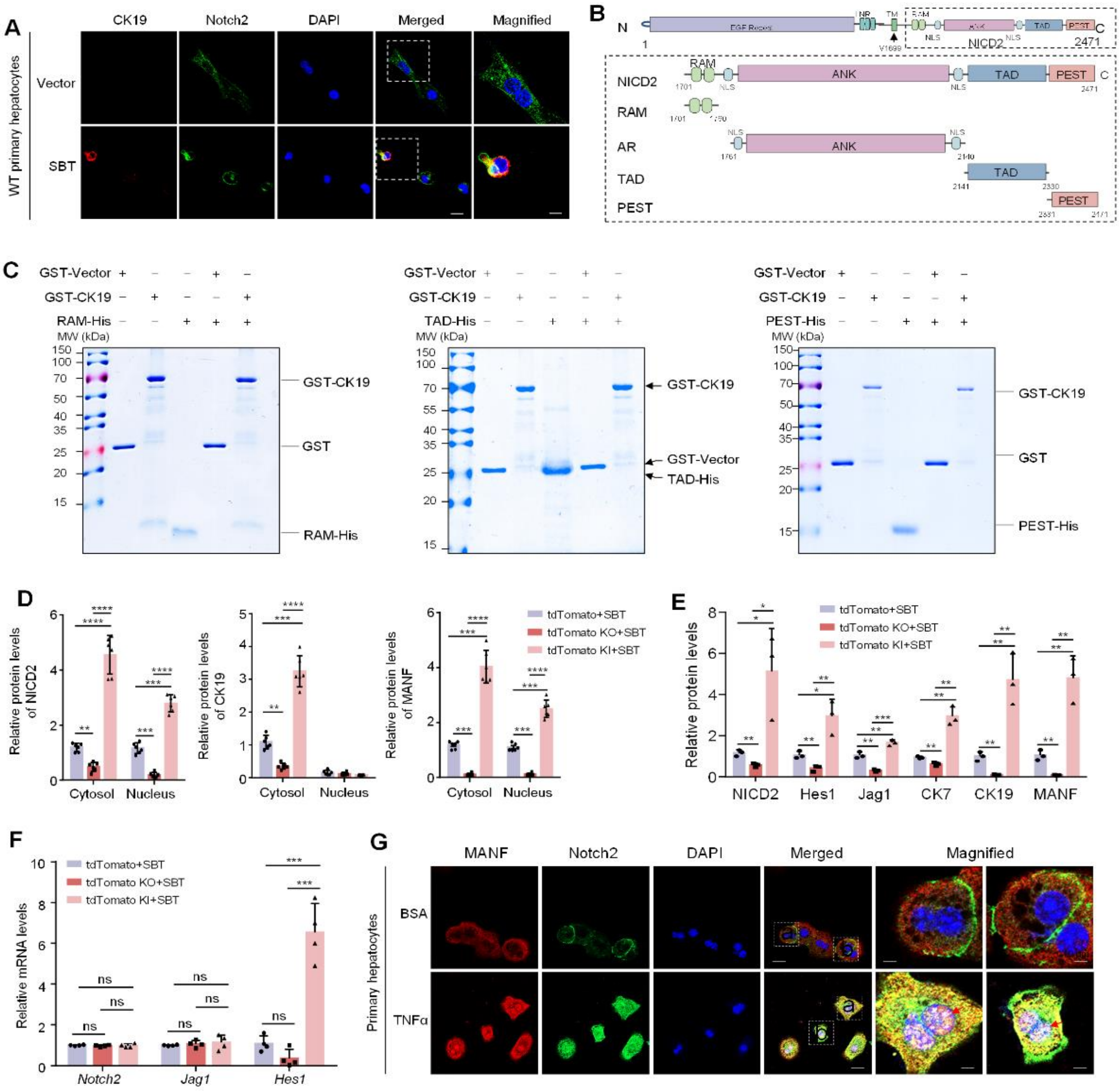
A
CK19
Notch2
DAPI
Merged
Magnified
WT primary hepatocytes
Vector
SBT
B
N
C
NICD2
RAM
ANK
TAD
PEST
AR
NLS
2471
C
GST-Vector
GST-CK19
RAM-His
TAD-His
PEST-His
MW (kDa)
GST
D
Relative protein levels of NICD2
Relative protein levels of CK19
Relative protein levels of MANF
Cytosol
Nucleus
tdTomato+SBT
tdTomato KO+SBT
tdTomato KI+SBT
E
Relative protein levels
NICD2
Hes1
Jag1
CK7
CK19
MANF
F
Relative mRNA levels
Notch2
Jag1
Hes1
G
MANF
Notch2
DAPI
Merged
Magnified
Primary hepatocytes
BSA
TNFα

254

255

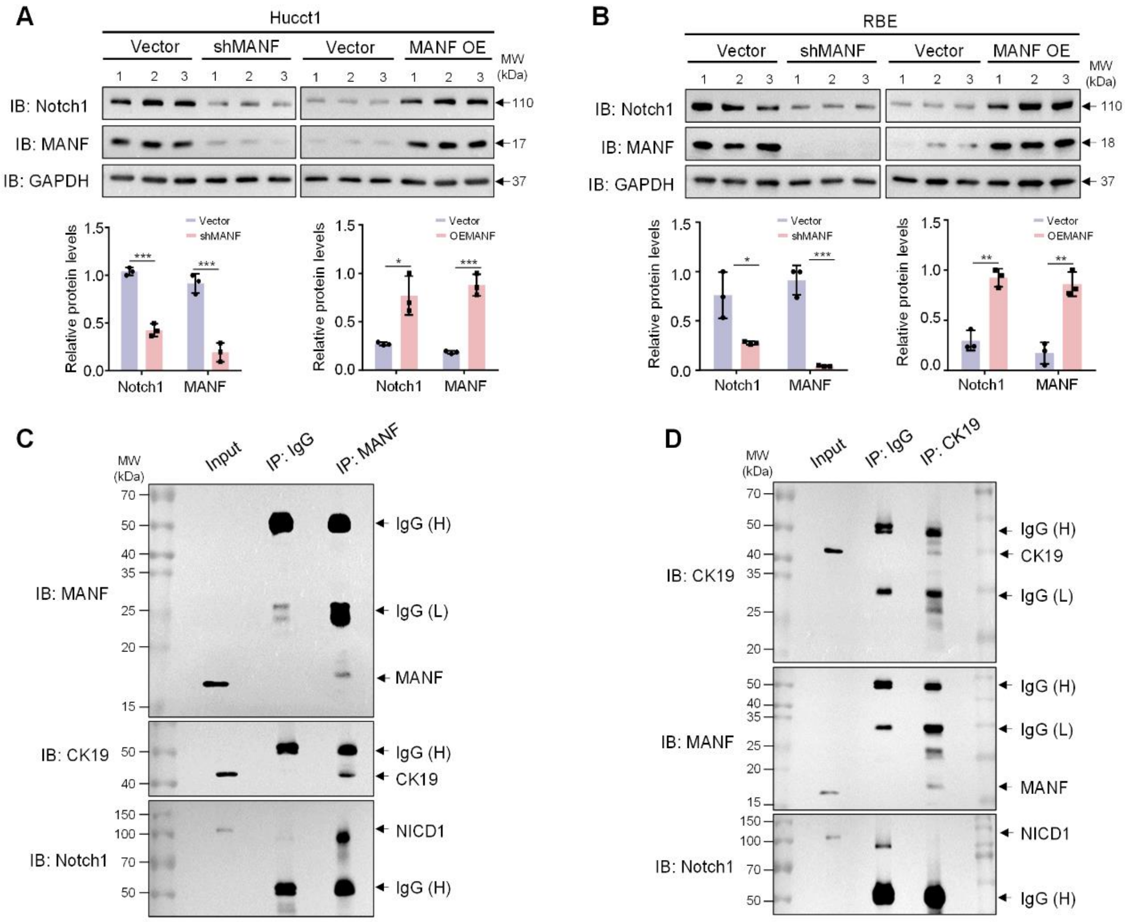
A
Hucct1
Vector
shMANF
Vector
MANF OE
MW (kDa)
IB: Notch1
IB: MANF
IB: GAPDH
110
17
37
Relative protein levels
Vector
shMANF
OEMANF
Notch1
MANF
B
RBE
Vector
shMANF
Vector
MANF OE
MW (kDa)
IB: Notch1
IB: MANF
IB: GAPDH
110
18
37
C
Input
IP: IgG
IP: MANF
IB: MANF
IB: CK19
IB: Notch1
IgG (H)
IgG (L)
MANF
CK19
NICD1
D
Input
IP: IgG
IP: CK19
IB: CK19
IB: MANF
IB: Notch1
IgG (H)
CK19
IgG (L)
MANF
NICD1

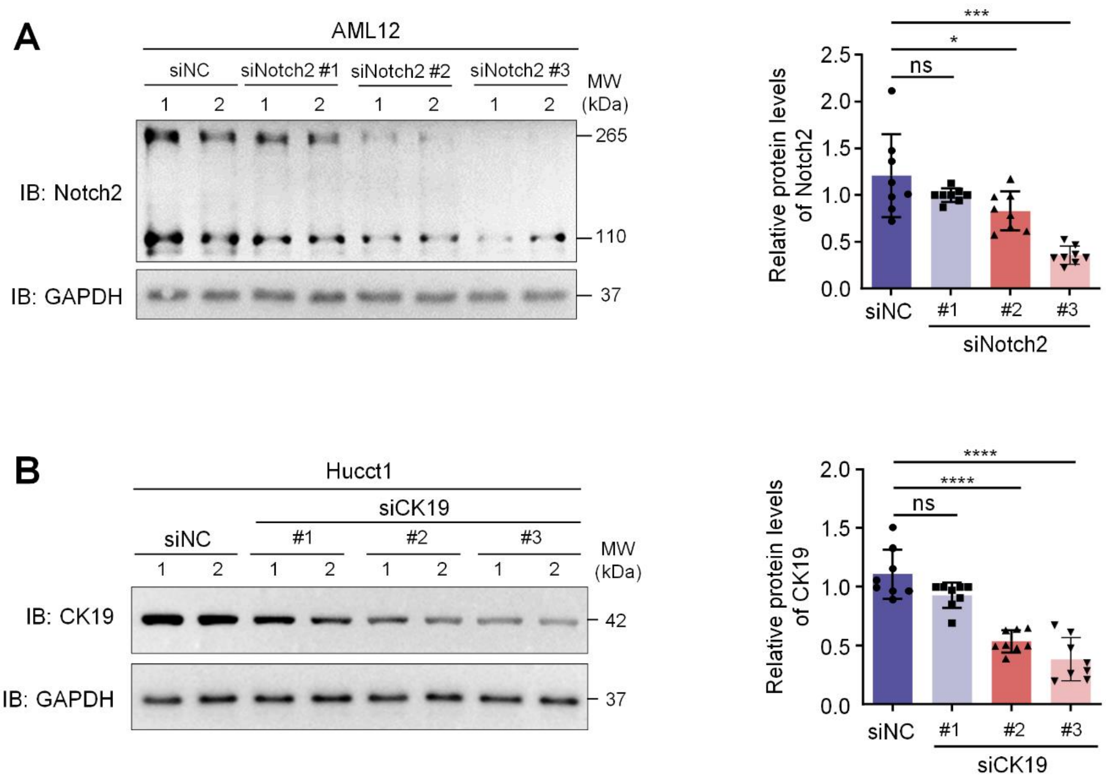
A
AML12
siNC
siNotch2 #1
siNotch2 #2
siNotch2 #3
MW (kDa)
IB: Notch2
IB: GAPDH
265
110
37
Relative protein levels of Notch2
siNC
#1
#2
#3
siNotch2
ns
*
***
B
Hucct1
siCK19
siNC
#1
#2
#3
MW (kDa)
IB: CK19
IB: GAPDH
42
37
Relative protein levels of CK19
ns
****
****
siCK19
