# Supplementary material for: Hepatic factor MANF drives hepatocytes reprogramming by detaining cytosolic CK19 in intrahepatic cholangiocarcinoma
Source: Cell Death Differ. 2025 Feb 19;32(8):1441–59. doi: 10.1038/s41418-025-01460-4 (PMC12325741; doi:10.1038/s41418-025-01460-4)

Fig 1B

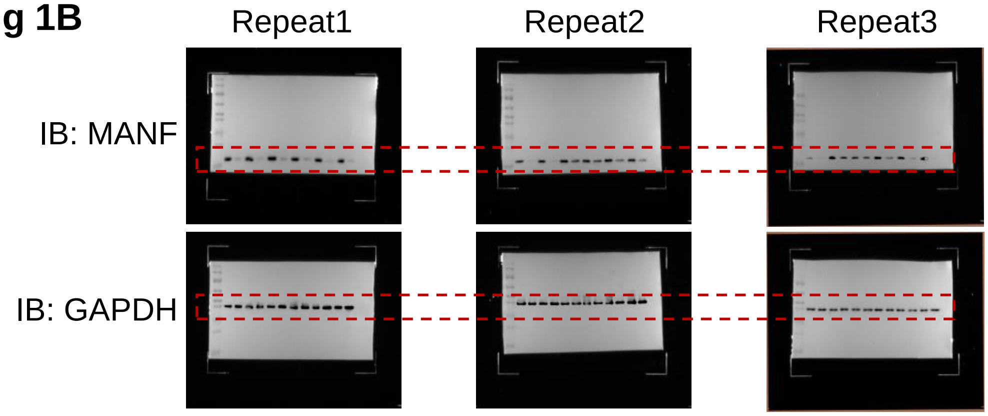

Fig 1I

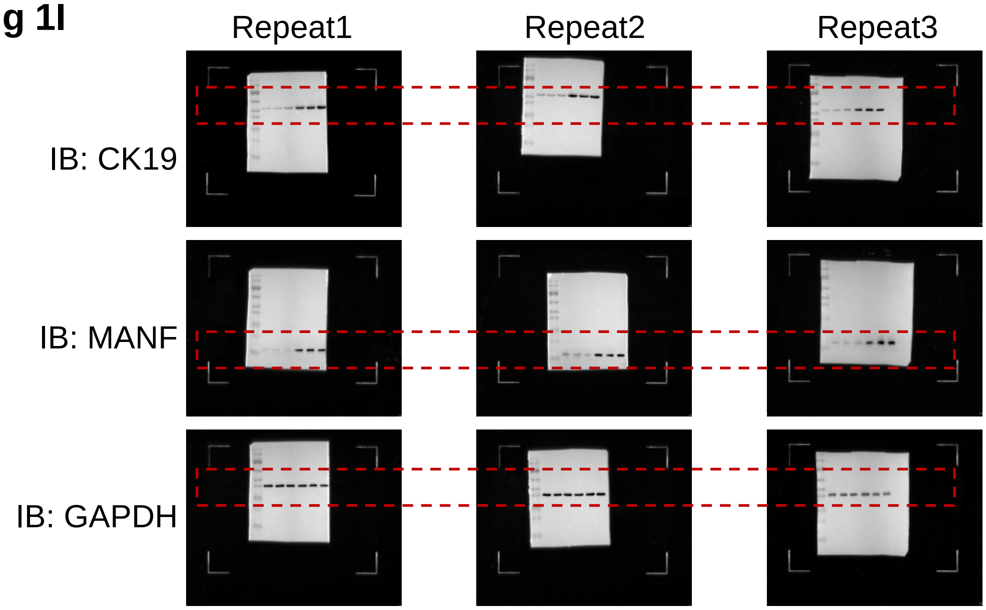

Fig 1N

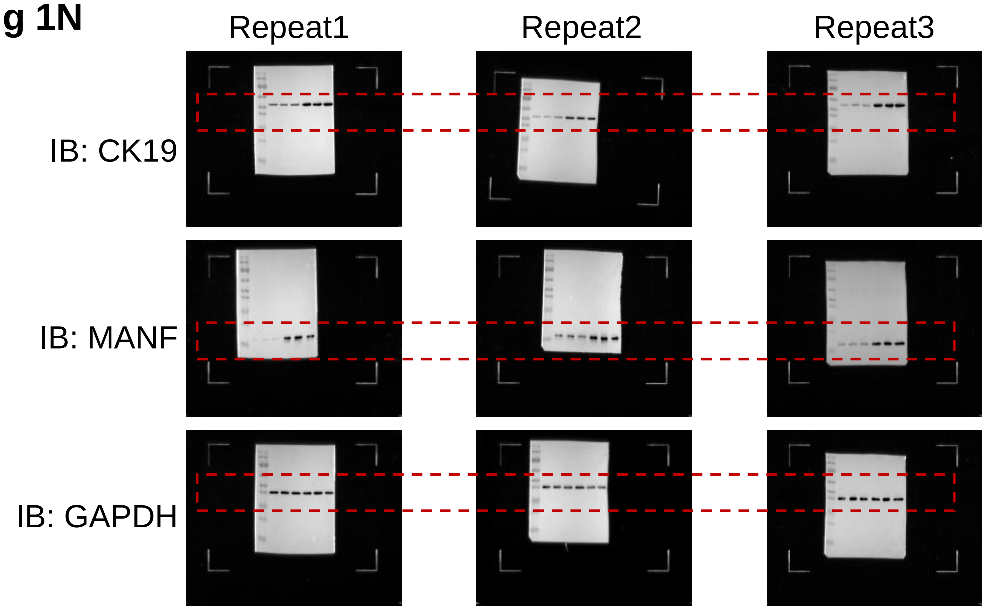

Fig 2C

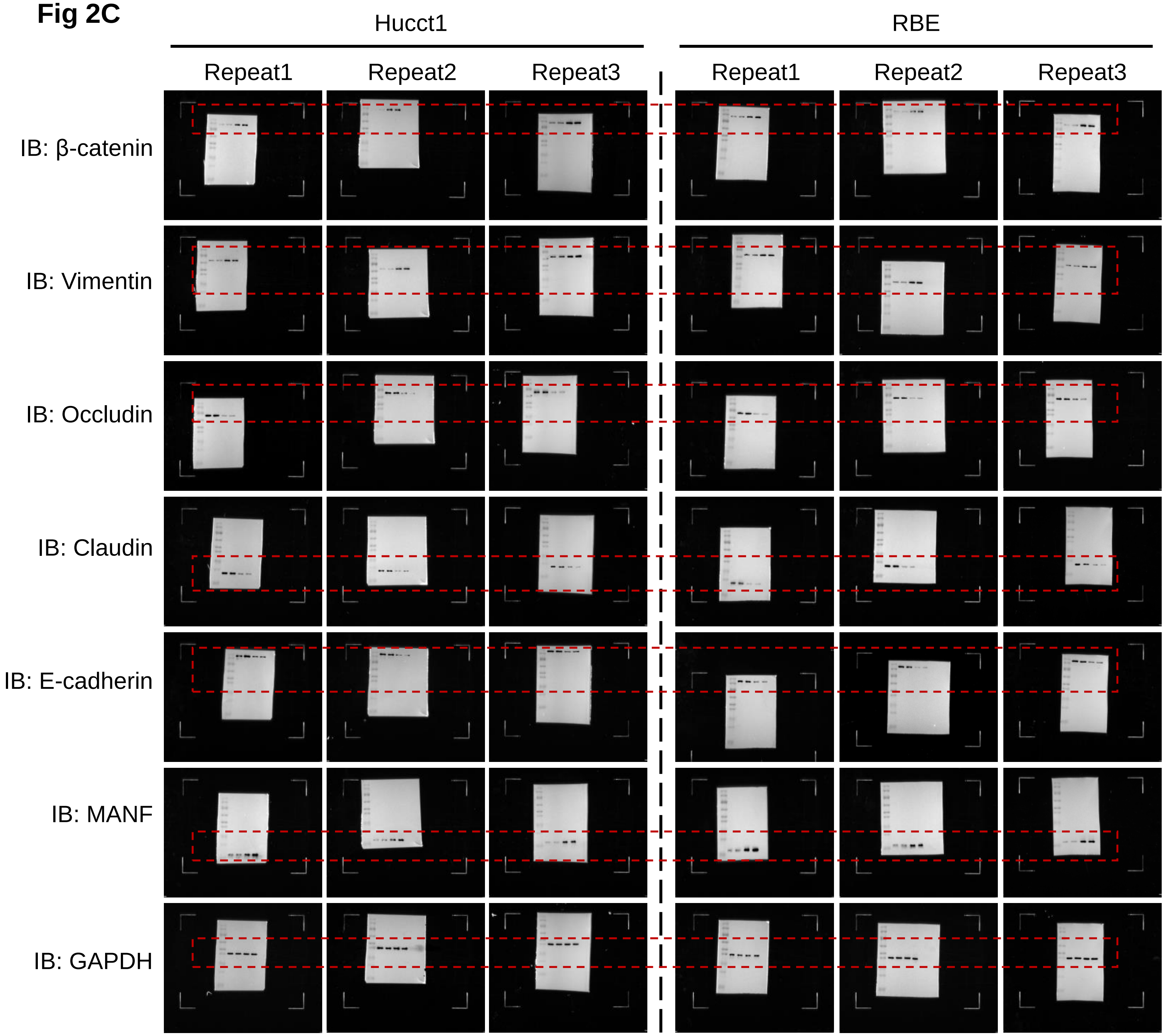
Hucct1
RBE
Repeat1
Repeat2
Repeat3
Repeat1
Repeat2
Repeat3
IB: β-catenin
IB: Vimentin
IB: Occludin
IB: Claudin
IB: E-cadherin
IB: MANF
IB: GAPDH

Fig 2F

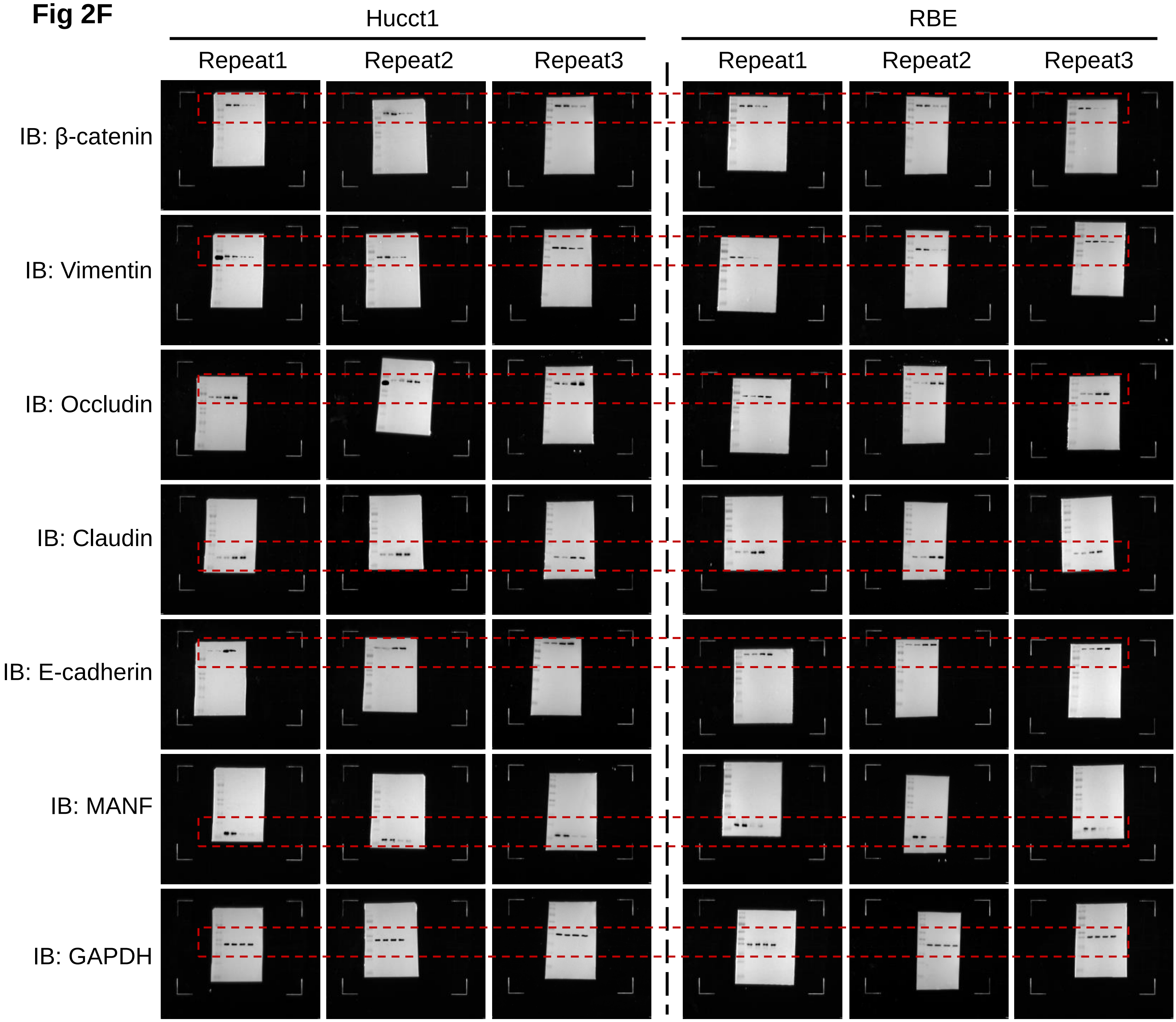
Hucct1
RBE
Repeat1
Repeat2
Repeat3
Repeat1
Repeat2
Repeat3
IB: β-catenin
IB: Vimentin
IB: Occludin
IB: Claudin
IB: E-cadherin
IB: MANF
IB: GAPDH

Fig 3H

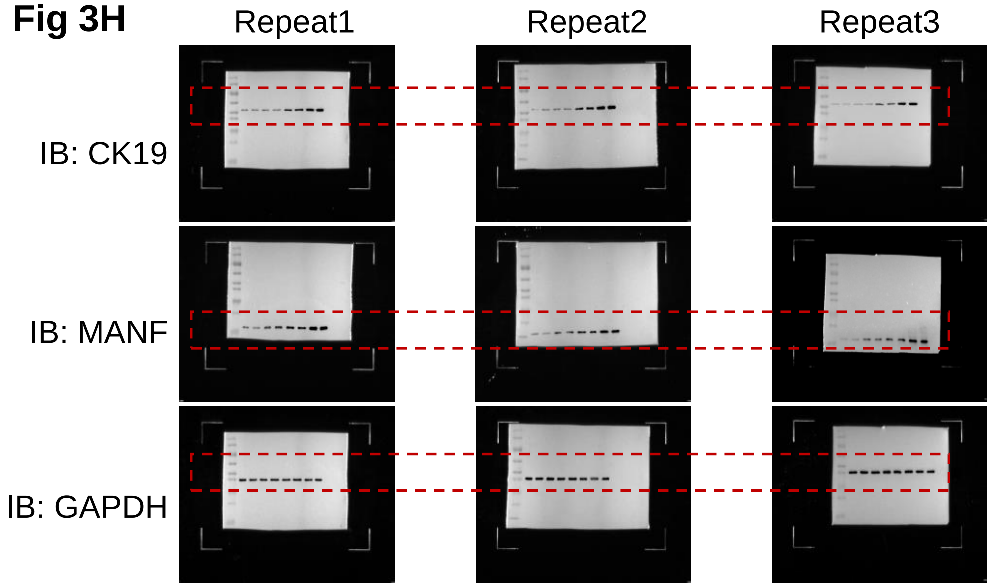

Fig 3P

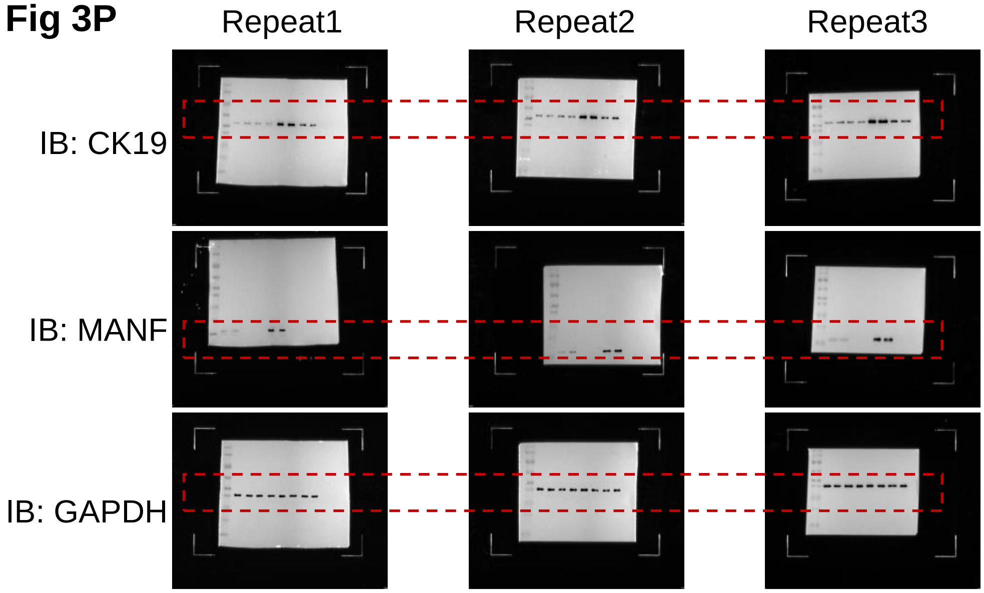

Fig 6G

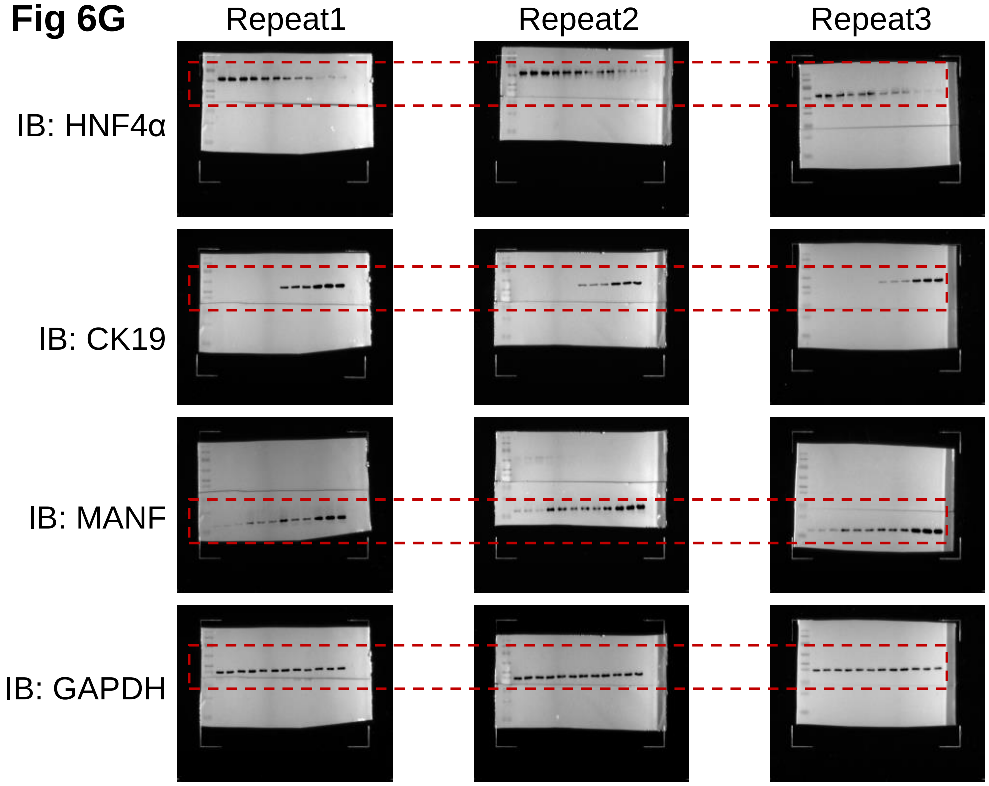

Fig 7A

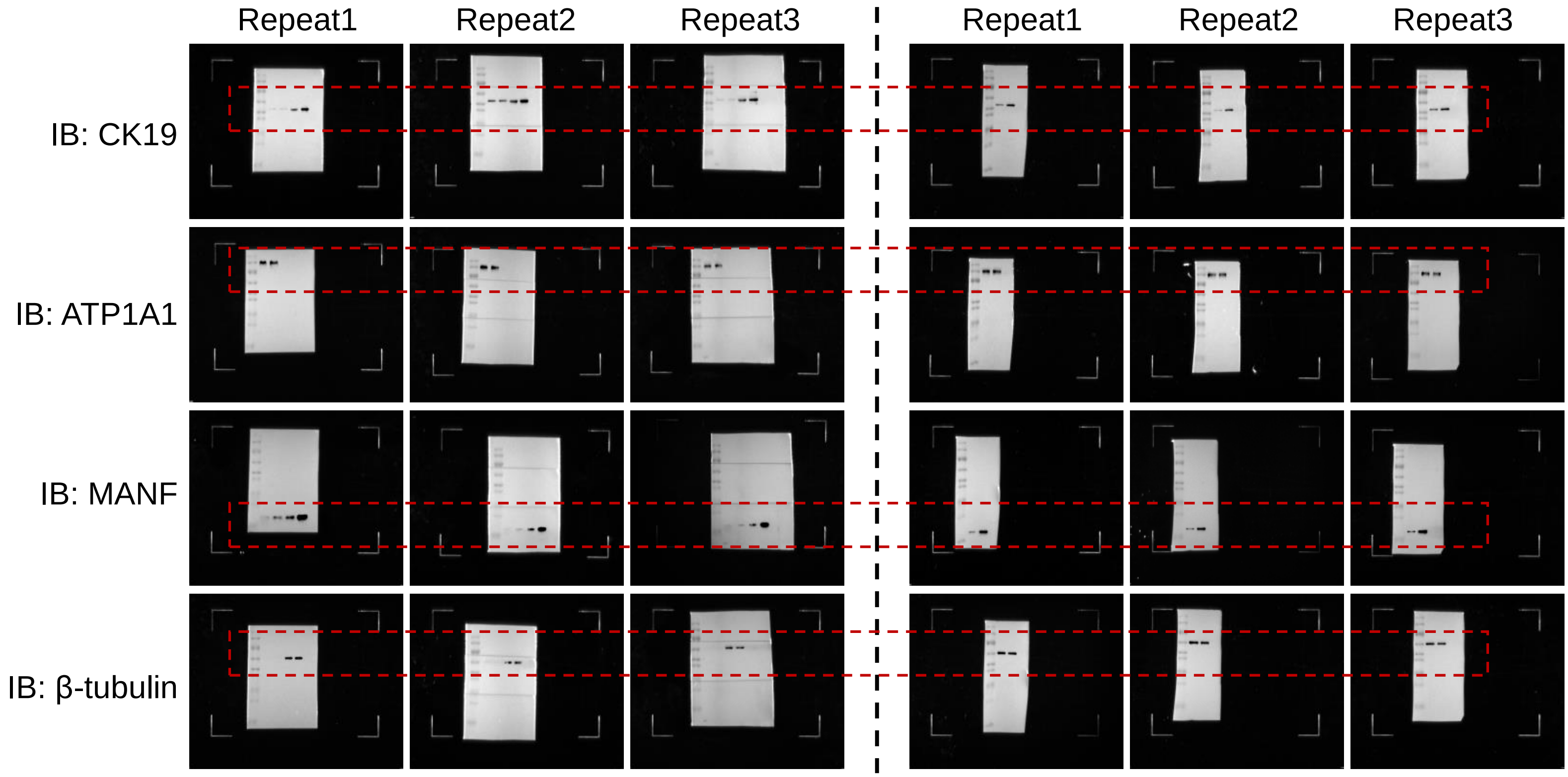

Fig 7C

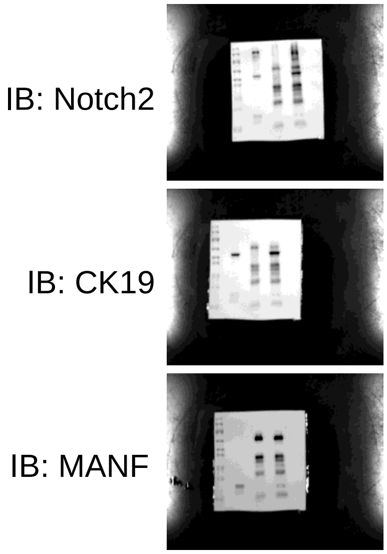

Fig 7D

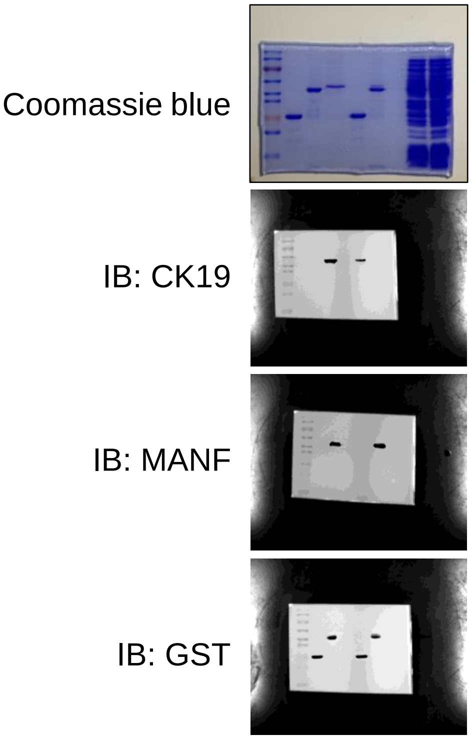

Fig 7E

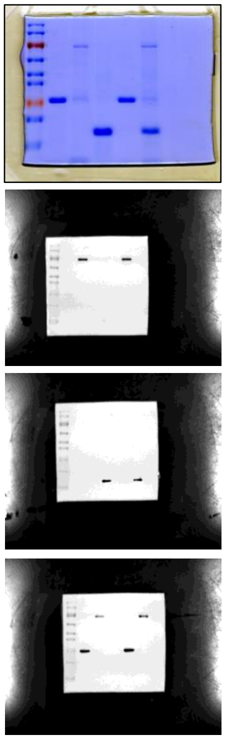

Fig 7G

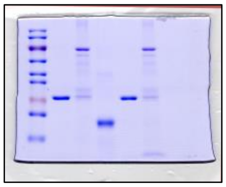

Fig 7H

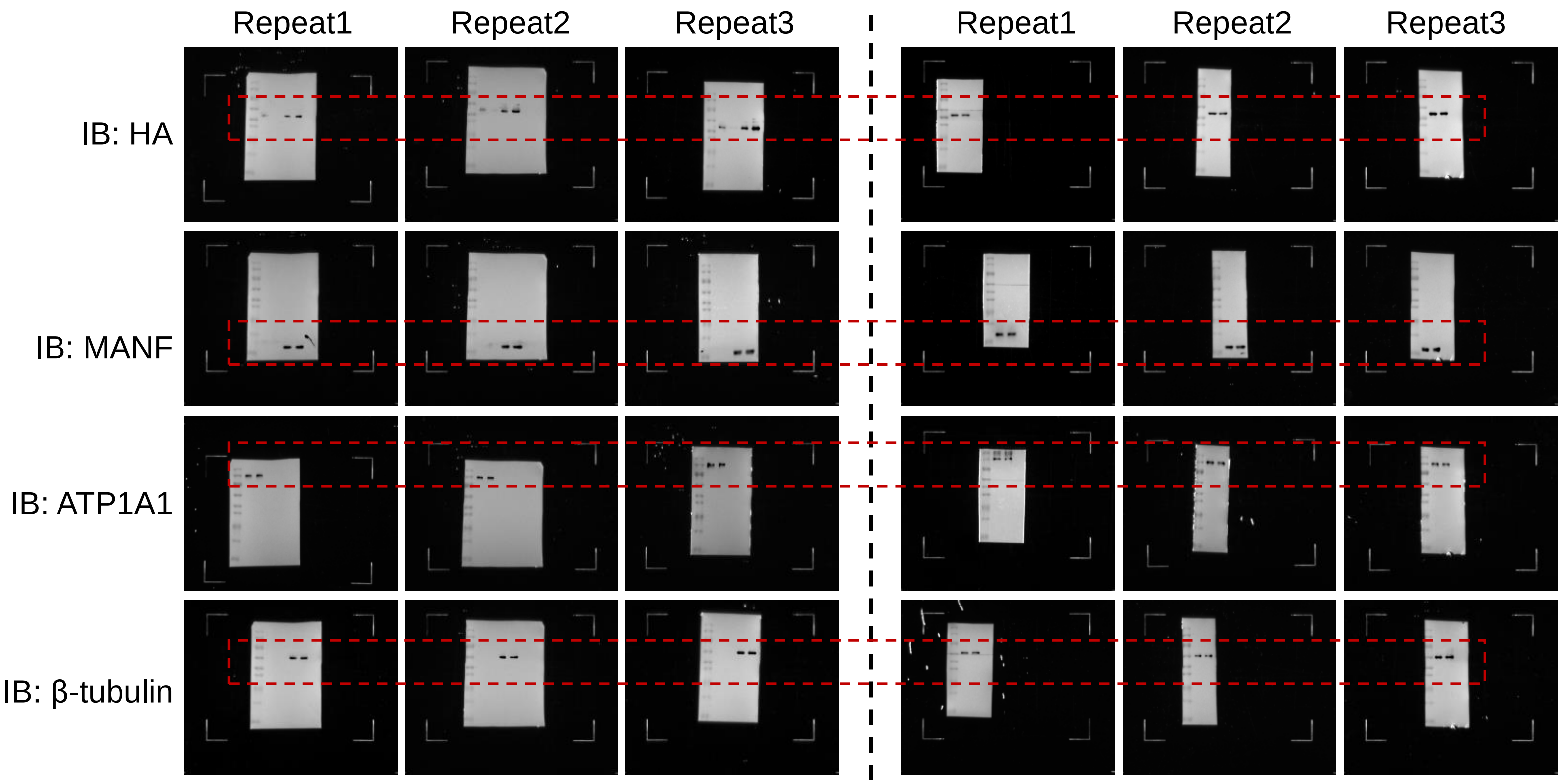

Fig 8A

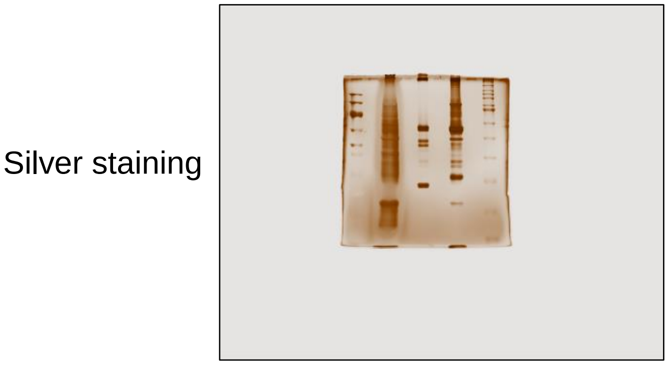

Fig 8J

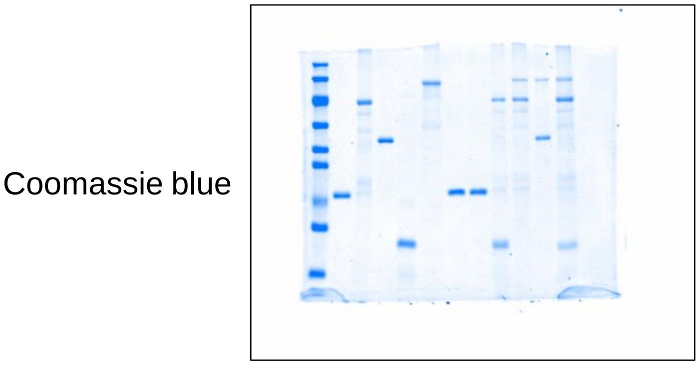

Fig 8D

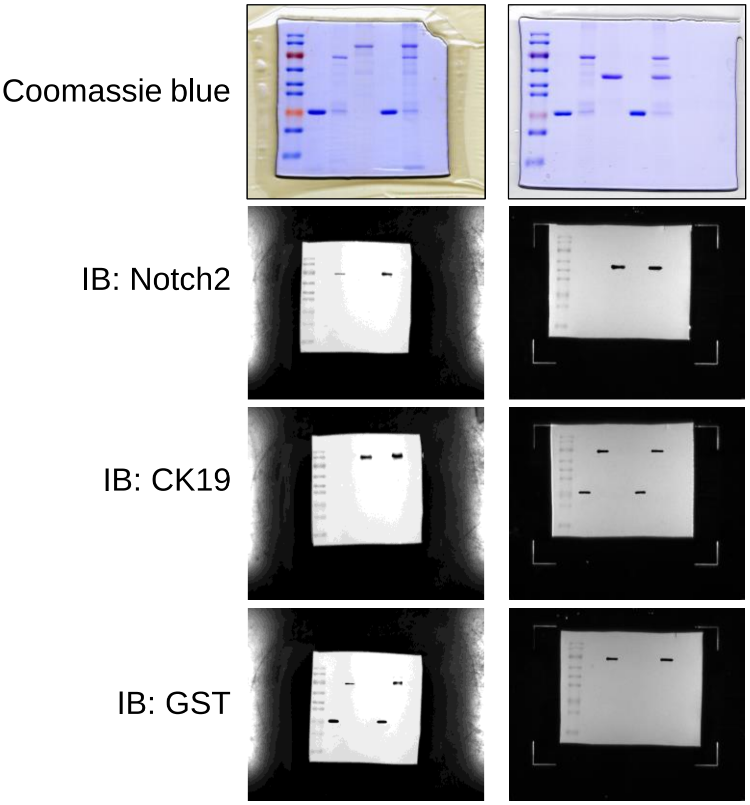

Fig 8F

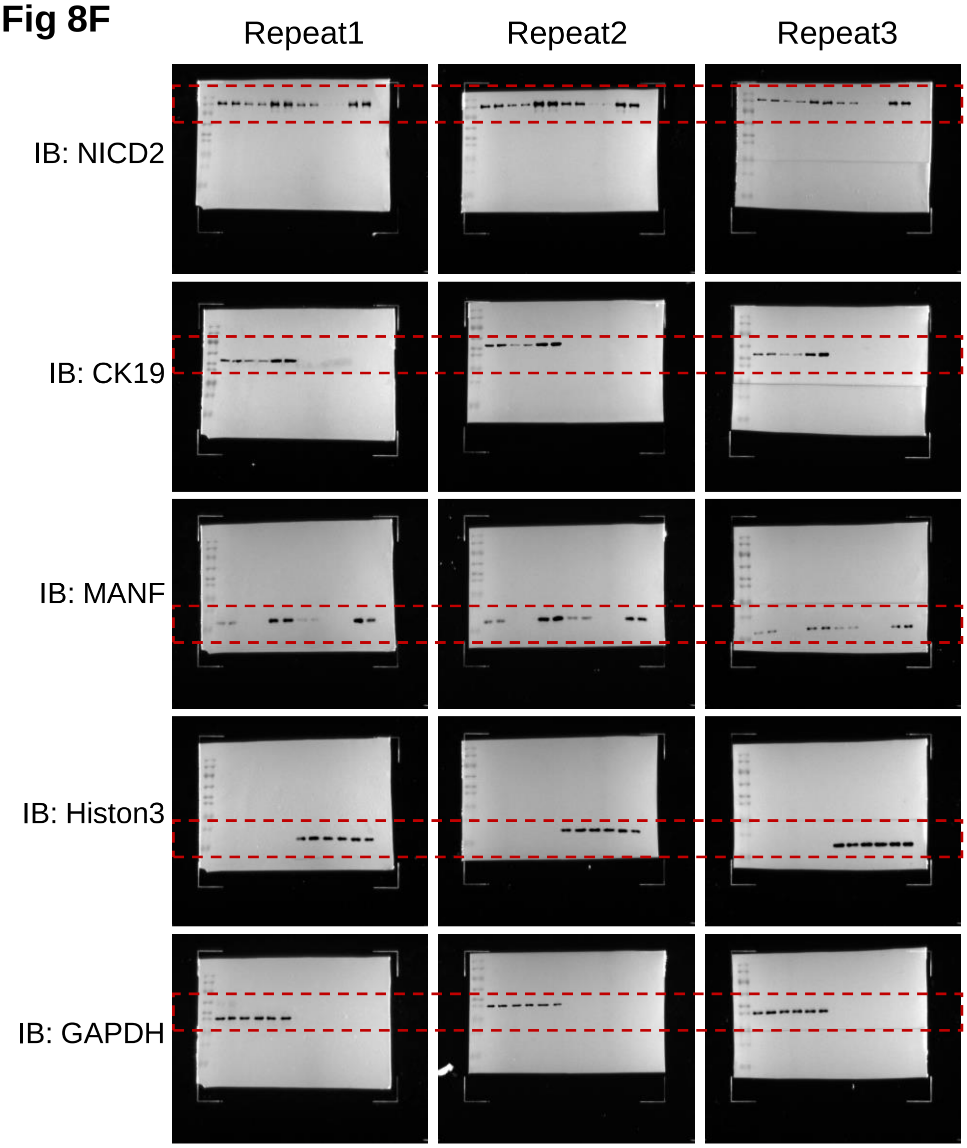
Repeat1
Repeat2
Repeat3
IB: NICD2
IB: CK19
IB: MANF
IB: Histon3
IB: GAPDH

Fig 8G

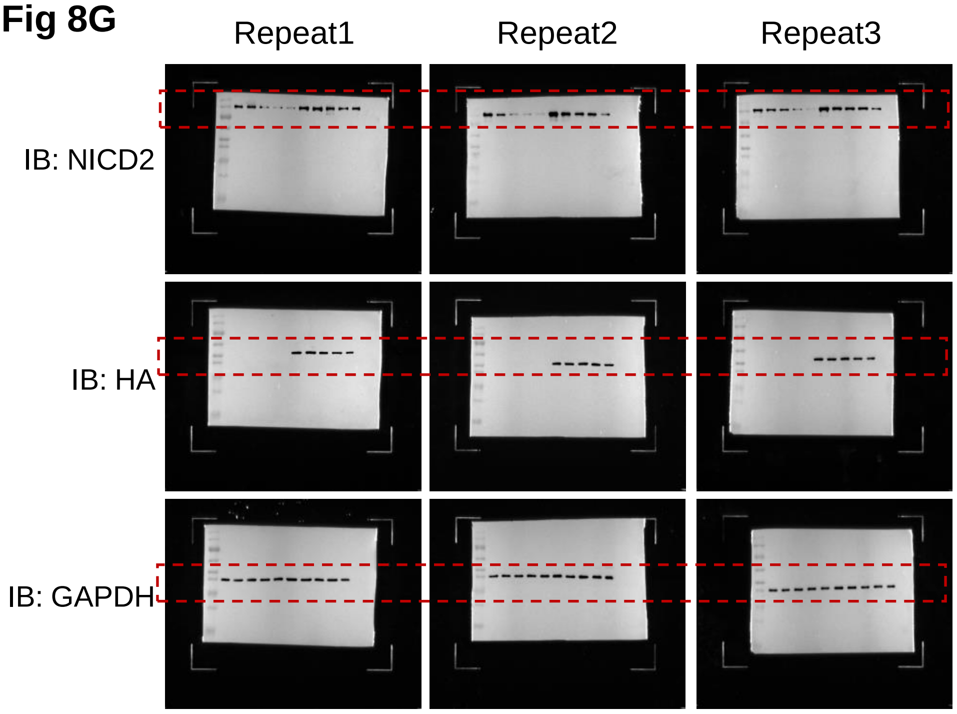
Repeat1
Repeat2
Repeat3
IB: NICD2
IB: HA
IB: GAPDH

Fig 8H

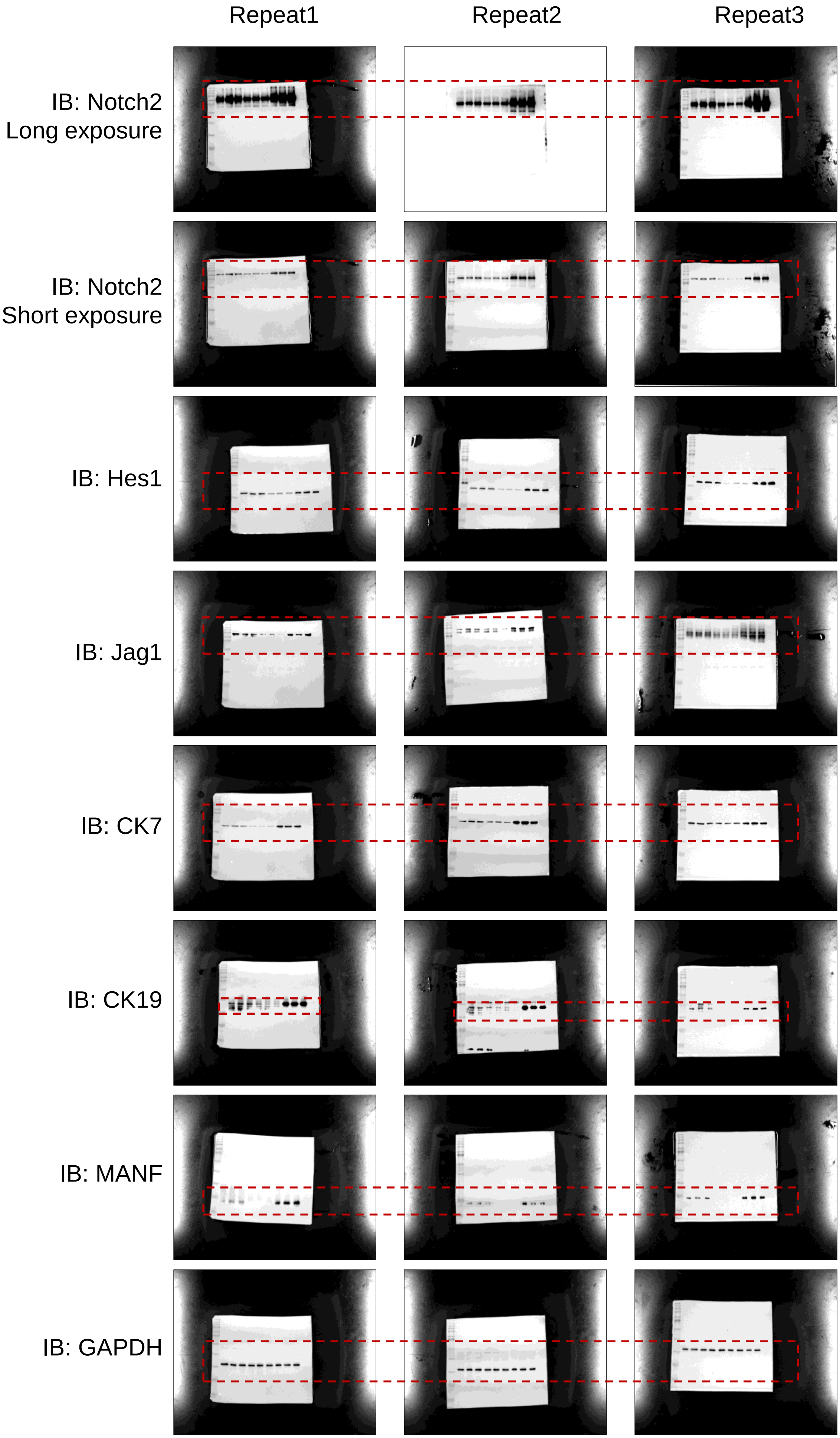

Repeat1
Repeat2
Repeat3
IB: Notch2
Long exposure
IB: Notch2
Short exposure
IB: Hes1
IB: Jag1
IB: CK7
IB: CK19
IB: MANF
IB: GAPDH

Fig 9J

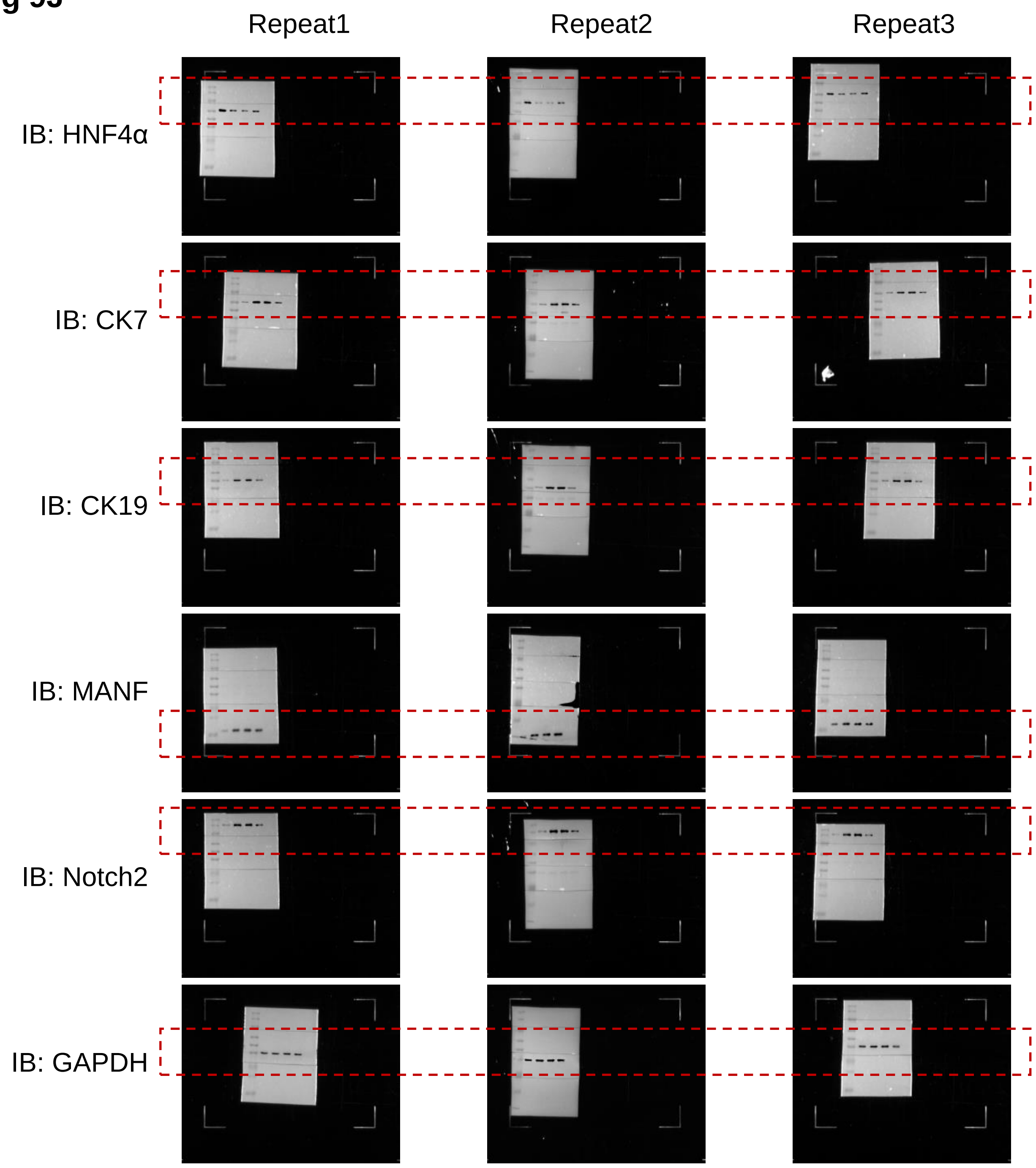
Repeat1
Repeat2
Repeat3
IB: HNF4α
IB: CK7
IB: CK19
IB: MANF
IB: Notch2
IB: GAPDH

Fig 9K

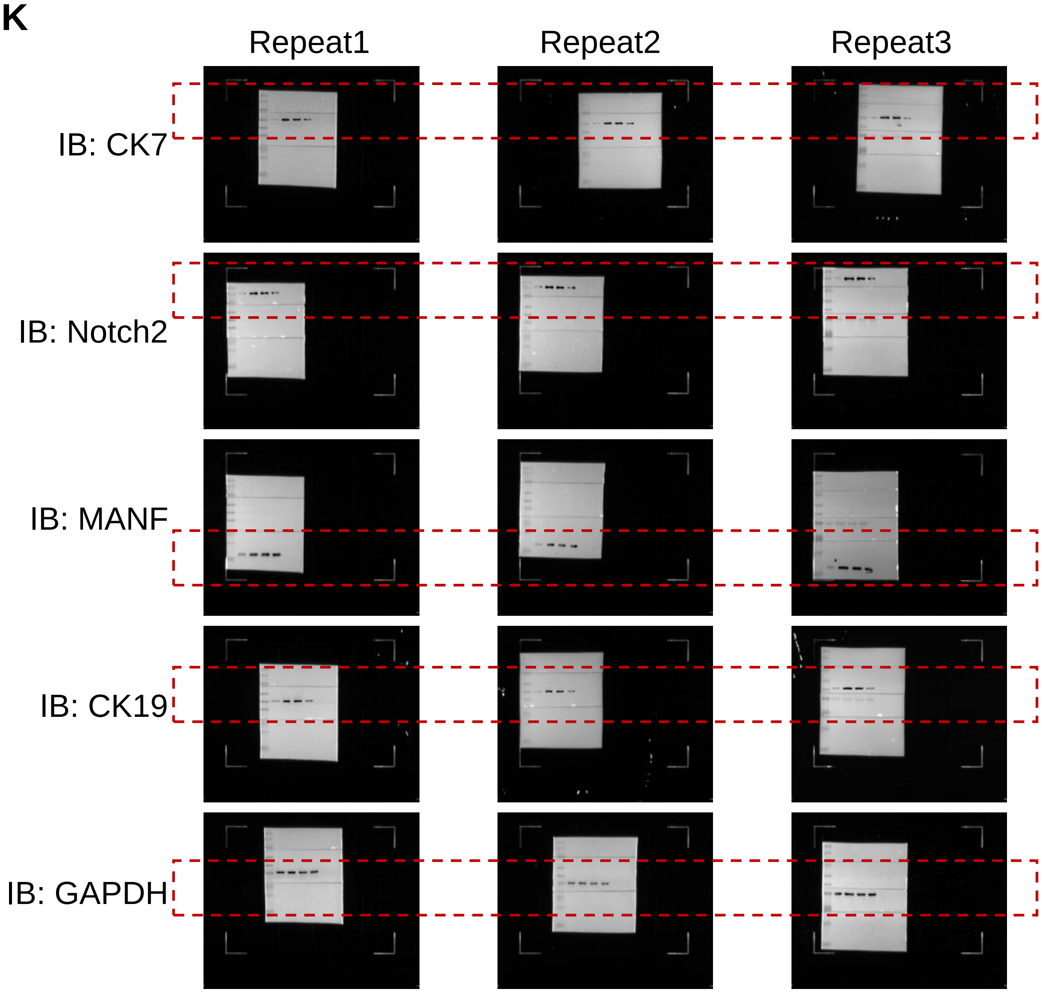
Repeat1
Repeat2
Repeat3
IB: CK7
IB: Notch2
IB: MANF
IB: CK19
IB: GAPDH

Fig 9L

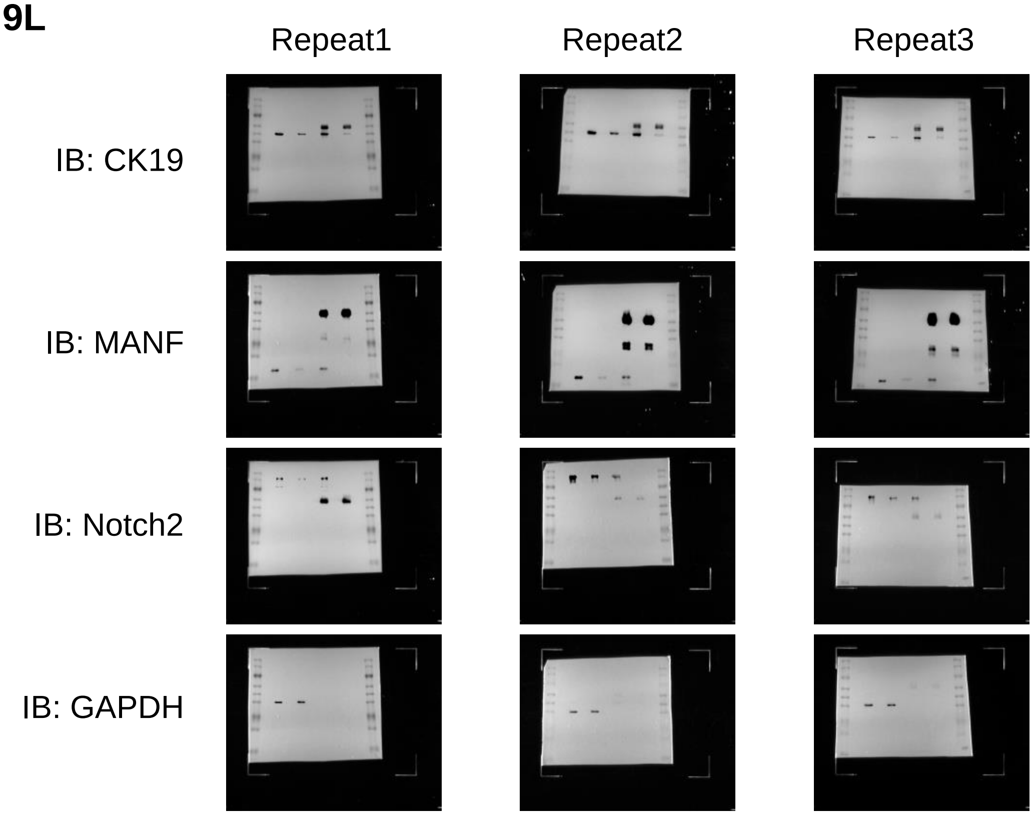
Repeat1
Repeat2
Repeat3
IB: CK19
IB: MANF
IB: Notch2
IB: GAPDH

SFig 4A

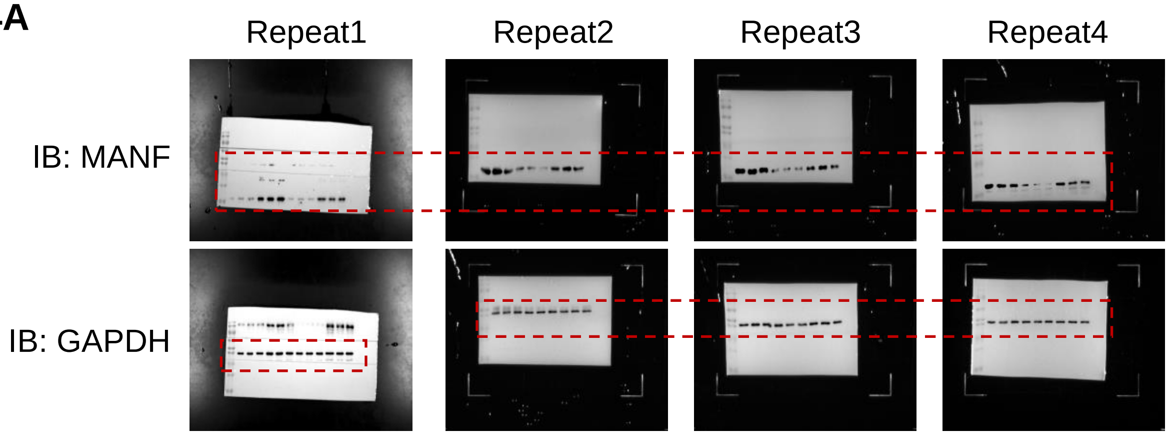

SFig 4C

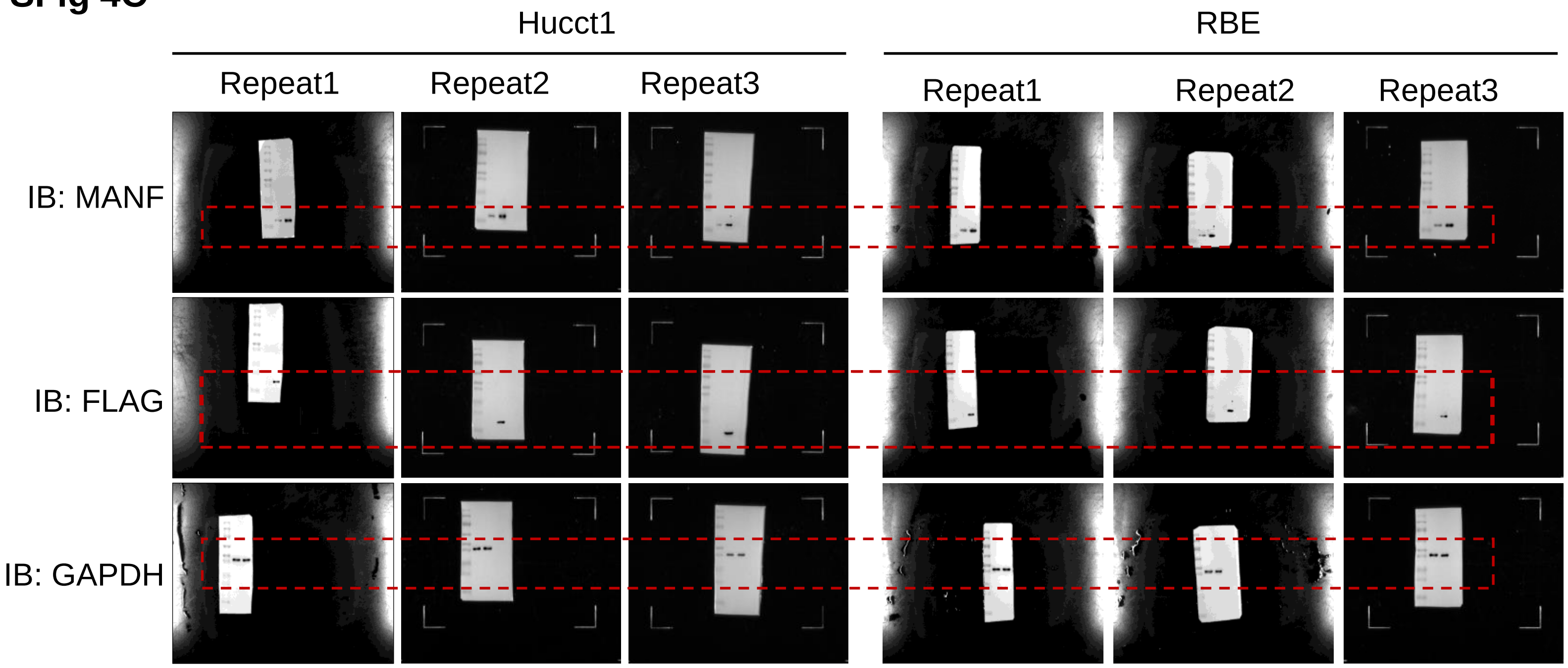

SFig 4E

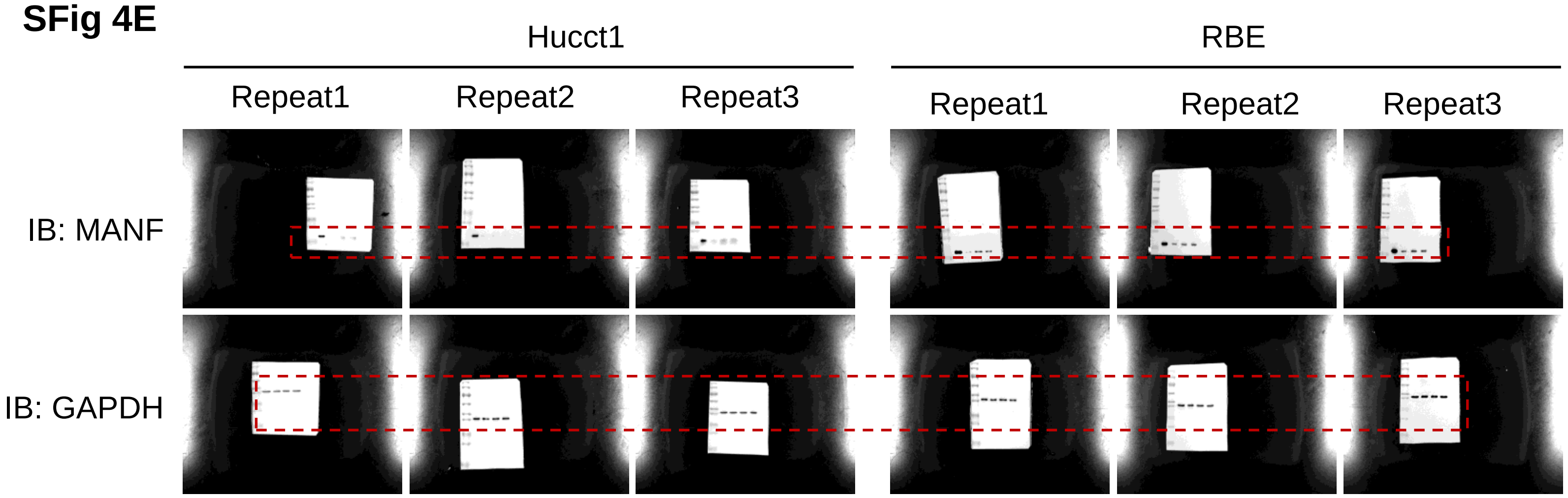

SFig 5A

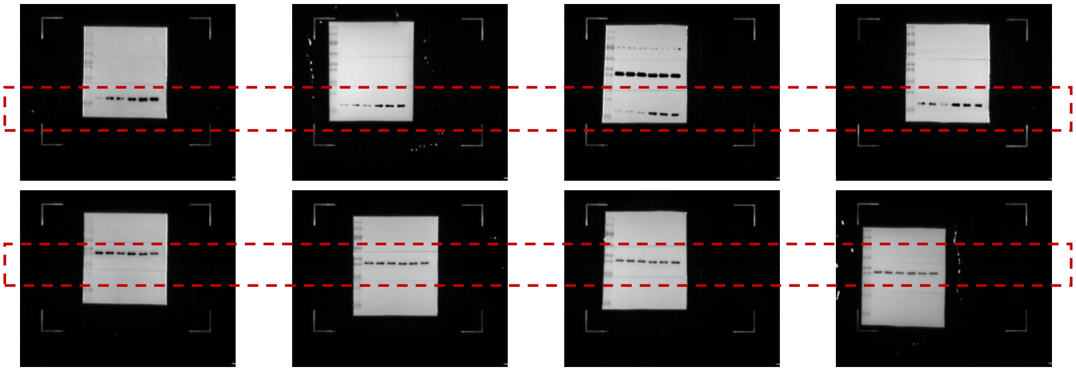

SFig 5B

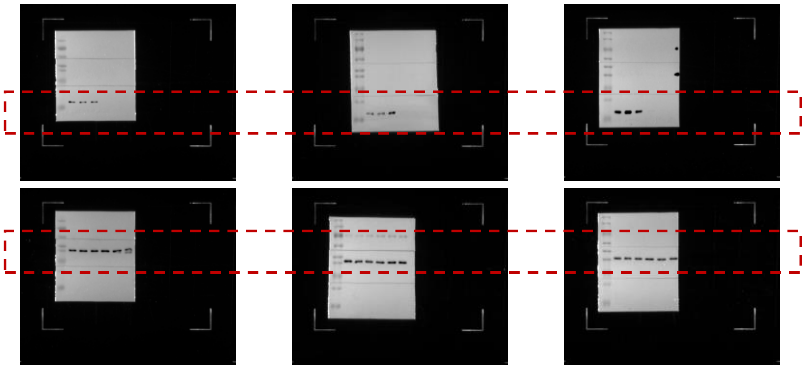

SFig 6M

SFig 6N

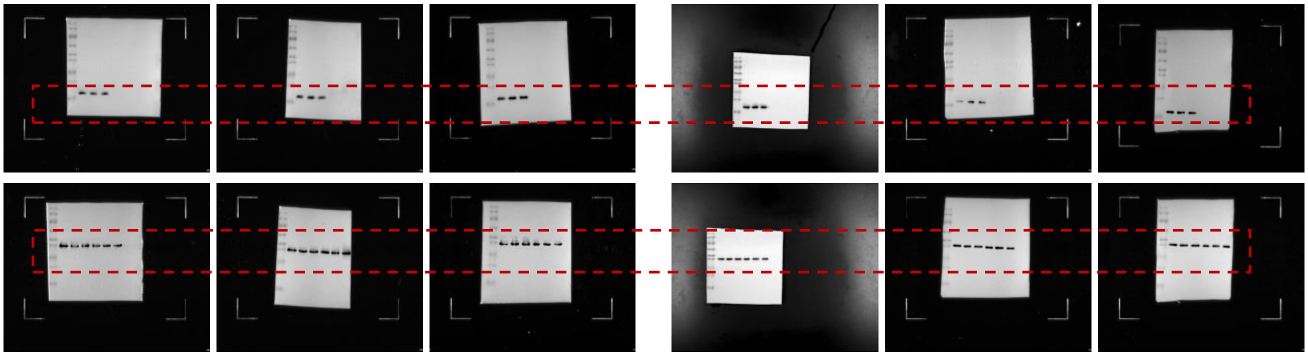

SFig 10C

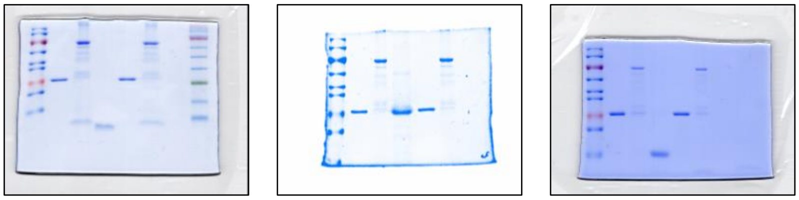

SFig 11A

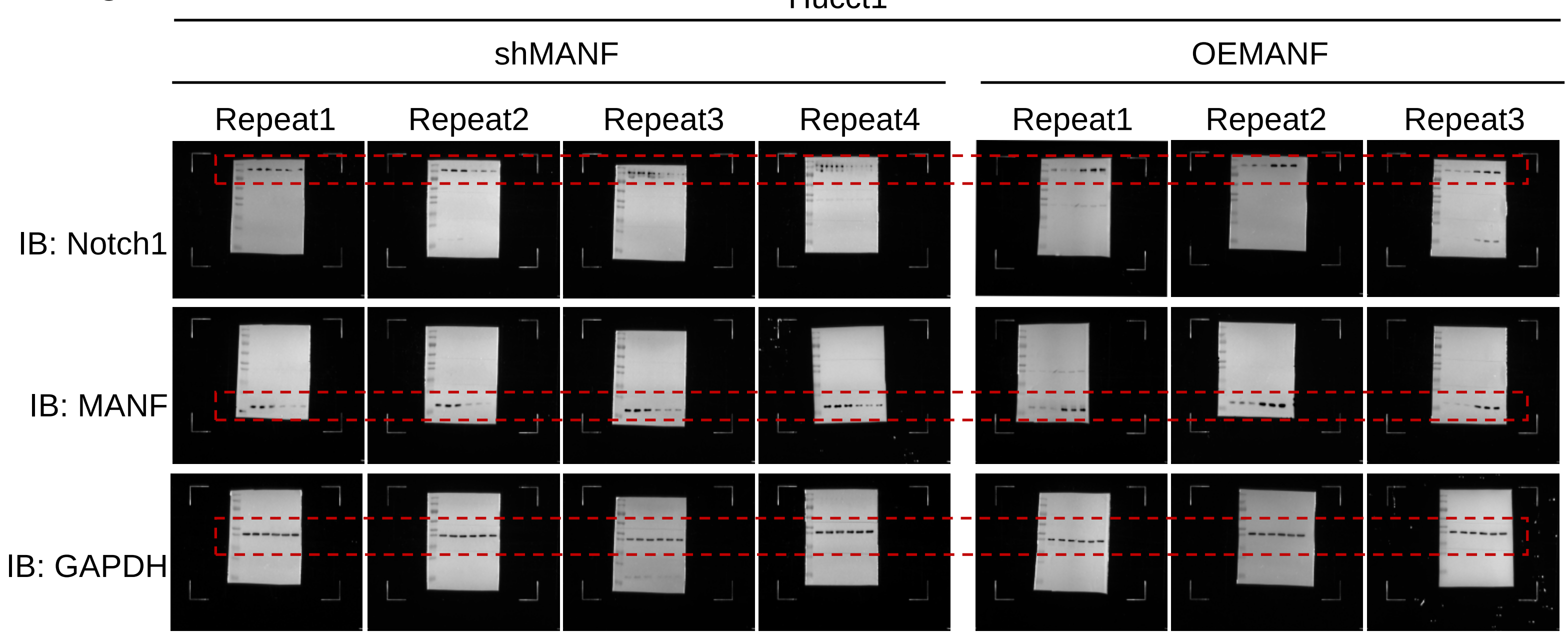

SFig 11B

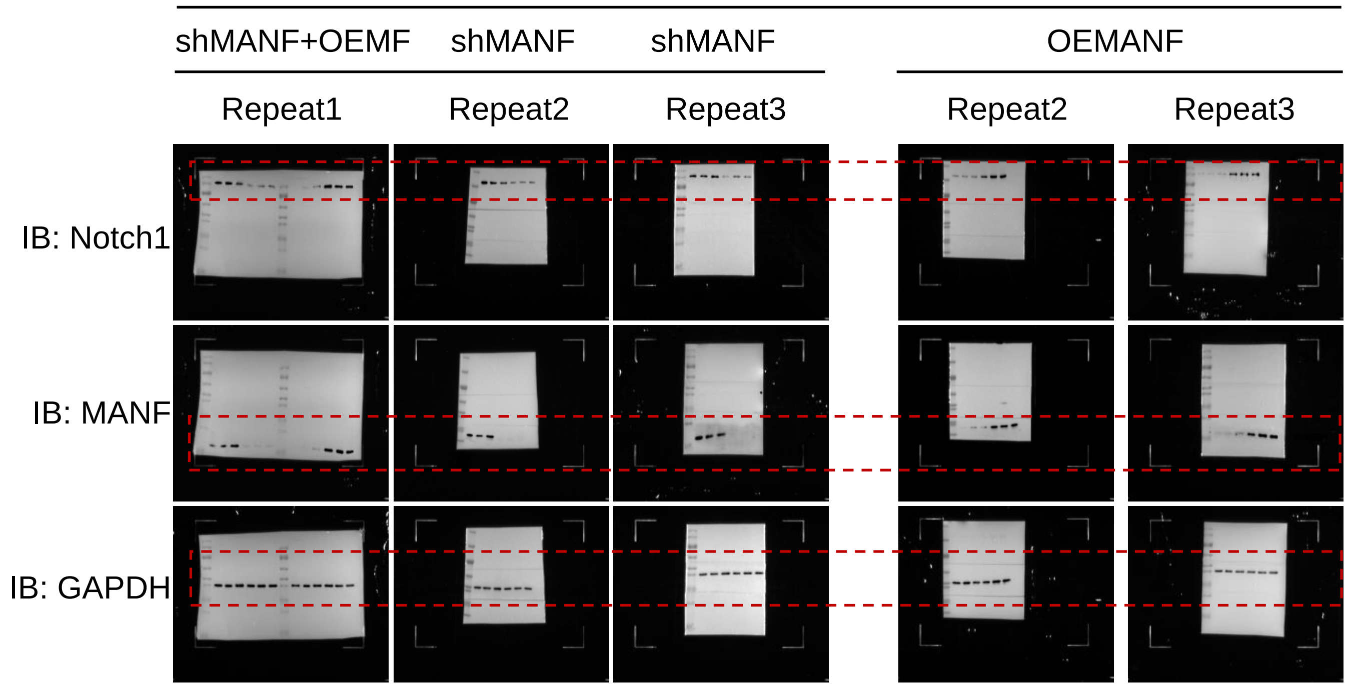

SFig 11C

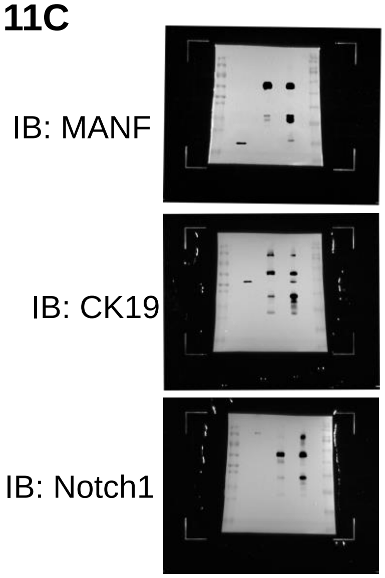

SFig 11D

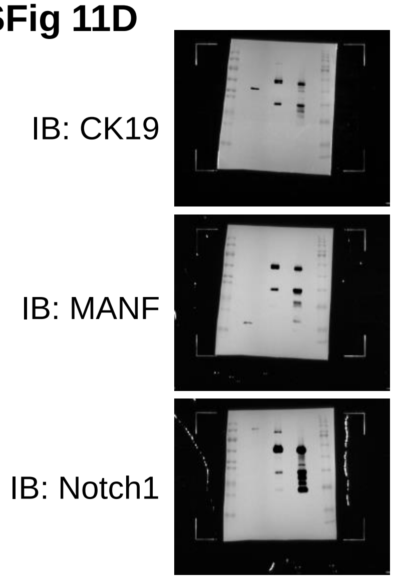

SFig 12A

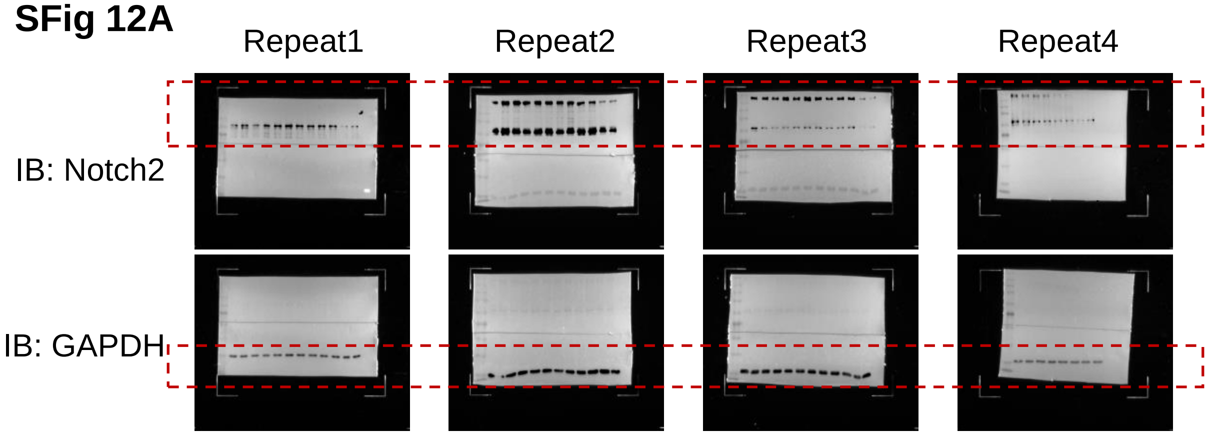

SFig 12B

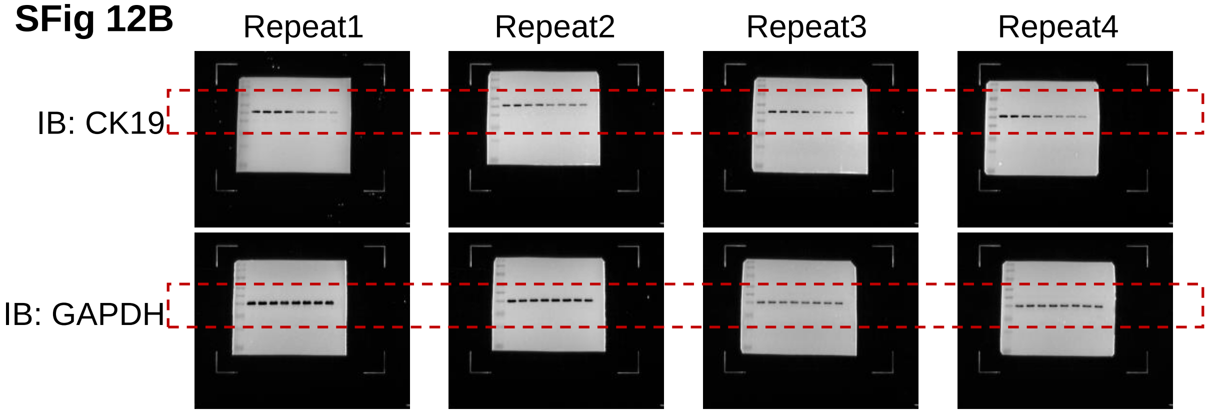

Supplement: Supplementary file 2 — Original Data Files [file 41418_2025_1460_MOESM2_ESM.pdf]
